# A Systematic Review on Fitness Testing in Adult Male Basketball Players: Tests Adopted, Characteristics Reported and Recommendations for Practice

**DOI:** 10.1007/s40279-021-01626-3

**Published:** 2022-02-04

**Authors:** Matthew Morrison, David T. Martin, Scott Talpey, Aaron T. Scanlan, Jace Delaney, Shona L. Halson, Jonathon Weakley

**Affiliations:** 1 School of Behavioural and Health Sciences, Australian Catholic University, Brisbane, QLD Australia; 2 School of Science, Psychology and Sport, Federation University Australia, Ballarat, VIC Australia; 3 Human Exercise and Training Laboratory, School of Health, Medical and Applied Sciences, Central Queensland University, Rockhampton, QLD Australia; 4 Sports Performance, Recovery, Injury and New Technologies (SPRINT) Research Centre, Australian Catholic University, Brisbane, QLD Australia; 5 Carnegie Applied Rugby Research (CARR) Centre, Institute of Sport, Physical Activity and Leisure, Leeds Beckett University, Leeds, UK

## Abstract

**Background:**

As basketball match-play requires players to possess a wide range of physical characteristics, many tests have been introduced in the literature to identify talent and quantify fitness in various samples of players. However, a synthesis of the literature to identify the most frequently used tests, outcome variables, and normative values for basketball-related physical characteristics in adult male basketball players is yet to be conducted.

**Objective:**

The primary objectives of this systematic review are to (1) identify tests and outcome variables used to assess physical characteristics in adult male basketball players across all competition levels, (2) report a summary of anthropometric, muscular power, linear speed, change-of-direction speed, agility, strength, anaerobic capacity, and aerobic capacity in adult male basketball players based on playing position and competition level, and (3) introduce a framework outlining recommended testing approaches to quantify physical characteristics in adult male basketball players.

**Methods:**

A systematic review of MEDLINE, PubMed, SPORTDiscus, Scopus, and Web of Science was performed following the Preferred Reporting Items for Systematic Reviews and Meta-Analyses guidelines to identify relevant studies. To be eligible for inclusion, studies were required to: (1) be original research articles; (2) be published in a peer-reviewed journal; (3) have full-text versions available in the English language; and (4) include the primary aim of reporting tests used and/or the physical characteristics of adult (i.e., ≥ 18 years of age) male basketball players. Additionally, data from the top 10 draft picks who participated in the National Basketball Association combined from 2011–12 to 2020–21 were extracted from the official league website to highlight the physical characteristics of elite 19- to 24-year-old basketball players.

**Results:**

A total of 1684 studies were identified, with 375 being duplicates. Consequently, the titles and abstracts of 1309 studies were screened and 231 studies were eligible for full-text review. The reference list of each study was searched, with a further 59 studies identified as eligible for review. After full-text screening, 137 studies identified tests, while 114 studies reported physical characteristics in adult male basketball players.

**Conclusions:**

Physical characteristics reported indicate a wide range of abilities are present across playing competitions. The tests and outcome variables reported in the literature highlight the multitude of tests currently being used. Because there are no accepted international standards for physical assessment of basketball players, establishing normative data is challenging. Therefore, future testing should involve repeatable protocols that are standardised and provide outcomes that can be monitored across time. Recommendations for testing batteries in adult male basketball players are provided so improved interpretation of data can occur.

**Clinical Trial Registration:**

This review was registered with the International Prospective Register of Systematic Reviews and allocated registration number CRD42020187151 on 28 April, 2020.

**Supplementary Information:**

The online version contains supplementary material available at 10.1007/s40279-021-01626-3.

## Key Points

**Table:** 

Success in basketball is predicated on players optimising multiple basketball-specific skills, which are influenced by many different physical characteristics. As a result, numerous tests have been introduced for the purposes of identifying talent and quantifying fitness across various samples of adult male players.
The wide range of tests and outcome variables reported in the literature illustrates the need to identify (a) physical characteristics that are most important for optimal match performance and (b) the most suitable tests and outcome variables for quantifying physical characteristics of interest.
Tests that are most suitable to identify talent may differ from tests that are most suitable for tracking changes in fitness and fatigue
Future research should focus on developing standardised testing batteries in conjunction with the International Basketball Federation and national governing bodies that contribute to meaningful normative data. A large international data set will facilitate an understanding of historical trends and allow basketball practitioners to become familiar with minimum and desirable fitness standards for their players.

## Introduction

Basketball has been reported by The Fédération Internationale de Basketball (FIBA) as the second most popular sport in the world [1]. The duration of a game varies depending on the governing body or federation, level of competition, as well as the age and sex of players [2]. However, the typical format for adult male matches are two 20-min halves (e.g., National Collegiate Athletic Association [NCAA]), four 10-min quarters (e.g., FIBA match-play), or four 12-min quarters (e.g., National Basketball Association [NBA]) [2]. Basketball is typically played on a wooden court with playing areas of 28.7 m × 15.2 m (NBA) or 28 m × 15 m (FIBA). Basketball teams consist of up to 12 players per team with five players competing for each team on the court at any one time during matches. The traditional five on-court playing positions include point guard, shooting guard, small forward, power forward, and centre, which are often categorised as backcourt (i.e. point guard and shooting guard) and frontcourt players (i.e., small forward, power forward, and centre) [3, 4].

The physical demands of a basketball game have been readily investigated [5–14]. Given the intermittent nature and varying positional demands involved in basketball match-play, a range of well-developed physical characteristics are thought to be required by basketball players [15–19]. During basketball matches, players are required to cover distances between 4400 and 7500 m, which consists predominantly of jogging, sprinting, jumping and changes in direction [12]. While frequently reaching speeds in excess of 7 m·s^−1^_,_ professional backcourt and frontcourt players have been reported to perform (mean ± standard deviation) 42 ± 6 and 56 ± 2 jumps per game, respectively [20]. Furthermore, players of all positions are required to execute shuffling movements at varied intensities while defending opposing players [12] and must be able to quickly identify and respond to the movements of opponents, challenging their agility, lateral movement and acceleration capabilities [21]. Although basketball is considered a non-collision sport, players will often block, push, and compete for possession with one another as they attempt to create and defend space on the court. The complex nature of basketball match-play clearly indicates the development of multiple physical characteristics can be advantageous to optimise match performance. However, it is important to be able to measure these physical characteristics independently of skill as physical capacities and skill often require different training stimuli to develop.

To assess the physical characteristics of basketball players, it is essential that tests are valid and reliable to ensure basketball practitioners can use the data to make informed decisions regarding training prescription, guiding return to play processes following injury, quantifying individual player progression, profiling and ranking players, and monitoring player performance and fatigue [22–26]. Researchers and practitioners often implement a diverse combination of tests that assess general physical characteristics (e.g., linear sprint speed) [4, 27, 28], as well as specialised tests that integrate sport-specific skills aimed to replicate certain basketball-specific demands (e.g., dribbling speed tests) [29–31]. However, the wide variety of tests and methods implemented can make it difficult to compare the physical characteristics of adult male basketball players within and between different competition levels. The array of available testing options makes it difficult to understand the physical characteristics required for successful performance in adult male basketball players. Therefore, to help support the quantification and comparison of physical characteristics in adult male basketball players, it is important to identify the most important and desirable characteristics for match performance and report the most common tests and outcome variables used to assess the physical characteristics.

We are unaware of any study that has provided a comprehensive analysis of tests and outcome variables used to assess the physical characteristics of adult male basketball players across all playing levels and positions. While Ziv and Lidor [32] reviewed the physical characteristics of professional male and female basketball players, over a decade has passed since this review was published and substantial growth in the basketball literature science has since occurred. Additionally, Mancha-Triguero et al. [33] reviewed tests used to assess the physical characteristics of high-level male and female players but the range of tests reported were limited with outcome data from each test not provided. Consequently, no review exists examining the tests used and the physical characteristics reported in combination in adult male basketball players from a range of competition levels. Given the world-wide popularity of basketball, it is prudent to review the tests used to quantify the physical characteristics of adult male basketball players across different competition levels. Due to the extensive evidence available on male basketball players, it is important to consolidate the current literature for basketball researchers and practitioners alike to develop a clear understanding of current practices in this population. A summary of basketball tests can support basketball practitioners when making decisions based on test results. Furthermore, larger samples of normative data aggregated across studies can lead to comprehensive profiling and benchmarking of important physical characteristics in adult male basketball players. Therefore, the purpose of this review is three-fold, (1) to identify tests and outcome variables used to assess physical characteristics in adult male basketball players across all competition levels, (2) to report a summary of anthropometric, muscular power, linear speed, change-of-direction speed, agility, strength, anaerobic capacity, and aerobic capacity in adult male basketball players based on playing position and competition level, and (3) to introduce a framework outlining recommended testing approaches to quantify physical characteristics in adult male basketball players.

## Methods

### Design and Search Strategy

A systematic review was conducted following the Preferred Reporting Items of Systematic Reviews and Meta-Analyses (PRISMA) statement [34]. This review was registered with PROSPERO (ID: CRD42020187151). The academic databases MEDLINE, PubMed, SPORTDiscus, Scopus, and Web of Science were searched from the earliest record until August 2020 to identify English-language, peer-reviewed, original research studies that investigated the tests used and/or physical characteristics of adult male basketball players. Studies were identified by searching key terms shown in Table 1. Search levels 1–4 were all linked by the Boolean operator ‘AND’. Search terms within each search level were joined with ‘OR’. When searching the PubMed and MEDLINE databases ‘young adults 19–24 years’ and ‘adults 19–44 years’ limiters were applied to the population age. No limiters were available to be used when searching Web of Science or SPORTDiscus. All search results were extracted and imported to reference manager software (EndNote X9; Thomson Reuters, Philadelphia, PA, USA).

**Table 1:** Search strategy used to identify articles

Search 1	Search 2	Search 3
(Male OR men)	(Adult OR senior)	Basketball
Search 4		
(Fitness testing OR physical characteristics OR Testing OR physical performance OR physical qualities OR physical profile OR anthropometric OR body height OR body weight OR skinfold OR body composition OR body fat OR power OR countermovement jump OR vertical jump OR broad jump OR muscular strength OR muscular endurance OR acceleration OR speed OR sprint OR running OR agility OR change of direction OR fitness OR physical fitness OR aerobic capacity OR repeated-sprint ability OR anaerobic capacity)		

### Assessment of Reporting Quality

The methodological quality of each study was assessed using a modified version of the Downs and Black checklist (Table 1 of the Electronic Supplementary Material [ESM]). This checklist has been used previously in systematic reviews related to sport science [35, 36] and is a valid method of assessing the quality of studies with observational study designs [37]. The modified version of the Downs and Black checklist was used because the included questions and criteria better align with the specific aims of this review compared with the traditional version of the checklist. The assessment included 12 questions (1–4, 6, 7, 10–12, 16, 18, 20) and was scored on a scale from ‘0’ (no, or unable to determine) to ‘1’ (yes) for each question. Scores were summed across questions for each study with a total score of ‘12’ reflecting the maximum score (highest quality) able to be attained.

### Study Selection

**Fig. 1:**
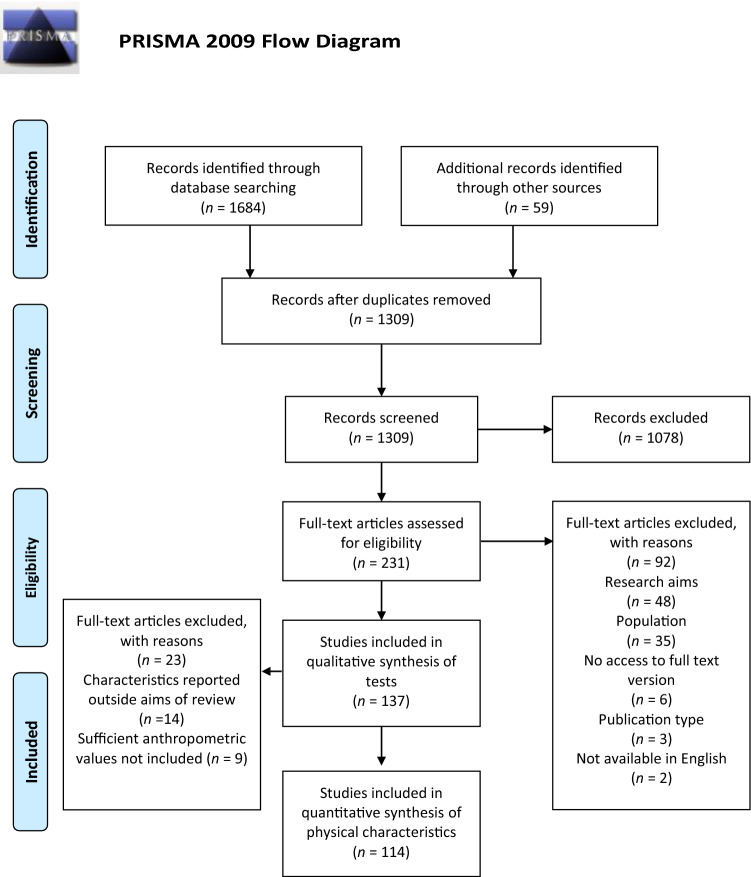
Flow of selection process of eligible studies for qualitative and quantitative synthesis

After duplicate studies were removed, two reviewers (MM and JW) independently screened all titles and abstracts against inclusion and exclusion criteria of the review. Studies deemed outside the scope of the review were removed. Any conflicts were settled by discussion between the reviewers with a third reviewer consulted for consensus if required. The full-text versions of the remaining studies were then reviewed for eligibility. To be eligible for inclusion, studies were required to: (1) be original research studies; (2) be published in a peer-reviewed journal; (3) have full-text versions available in English language; and (4) have the primary aim of reporting tests used and/or the physical characteristics of adult (i.e. ≥ 18 years of age) male basketball players. Studies were excluded from the review if they: (1) were systematic or narrative reviews; (2) were meta-analyses; (3) had the primary aim of investigating a nutritional supplement or ergogenic aid; (4) examined referees instead of players; (5) examined wheelchair players; or (6) examined players with a mean age under 18 years or competing in ‘junior’ competitions. The reference lists of the included studies were then manually reviewed for additional eligible studies. If further studies were identified, they were subjected to the same assessment previously described. Figure 1 outlines the selection process during the screening of studies. Data pertaining to the first aim of this review involved a qualitative synthesis of the available evidence, whereas a quantitative synthesis was used to address the second aim.

### Data Collection

Data extraction included study details (authors and publication year), all tests performed to quantify physical characteristics (i.e., height, body mass, wingspan, body fat percentage, muscular power, linear speed, change-of-direction speed, agility, strength, anaerobic capacity, and aerobic capacity), and the outcome variables derived from each test. If the methods of physical testing were not clearly outlined in the study, the tests were not included in the data extraction process. If the authors of the study did not administer the testing protocol as part of the study (e.g., they surveyed coaches for results [38]), the study was not included. If a test included a skill component (e.g., dribbling a basketball) or a series of basketball-specific movements (e.g., sprinting and then jumping), it was not included in the analysis.

After the tests were extracted, data relating to playing position and competition level were identified and reported. Competition levels were categorised as either amateur (club, volunteer, or recreational players), collegiate (university or collegiate players), representative (players selected to play in a representative team), semi-professional (some players are contracted or full time) or professional (all players on the team are contracted full-time athletes or competing in a country’s highest division or competition). Playing positions were reported as they were identified in the original text of each study. Additionally, outcome variables for anthropometric, muscular power, linear speed, change-of-direction speed, agility, strength, anaerobic capacity, and aerobic capacity tests were retrieved. For tests that had multiple outcome variables, after all data were collated, variables were counted, and the two most frequently used outcome variables were extracted. However, for linear speed and change-of-direction speed tests, only time was extracted because of the variability in other outcome variables reported across studies. Likewise, for assessments of strength, only one repetition maximum (1RM) performances were extracted from studies owing to the variability in other outcome variables. Data were extracted from each study using the raw values provided. In the case of an intervention study (e.g., the implementation of a resistance training programme [39]), baseline measurements were used. Furthermore, if multiple groups were included in a study, the control group was recorded to mitigate the bias of the intervention. To minimise any potential bias or confounding outcomes, studies that did not provide basic player information including age, height, body mass, and competition level were not included in the reporting of physical characteristics (the second aim of this review) but remained in the review to address the first aim. If data were presented using figures and raw data were not clearly available, the authors of the study were contacted to provide the raw values. If no response was received from the authors of a study, means and measures of distribution were extracted from figures in studies using WebPlotDigitizer v4.0 [40], which has been shown as a valid (*r* = 0.989, *p* < 0.001) and reliable (*r* = 0.997, *p* < 0.001) [41] tool for the extraction of raw values from figures. If a study reported variables using units in the Imperial system, they were converted to the Metric system to allow for clear comparison across studies.

To provide greater insight into the physical requirements of professional basketball players competing at the highest level, publicly available NBA Draft Combine data were downloaded from the league’s official website [42]. Data representing 100 players (10 players per year over 10 years) were synthesised and used as reference data to describe physical characteristics in this population. The mean, standard deviation, as well as minimum and maximum values for height (cm), body mass (kg), body fat percentage, wingspan (cm), Lane Agility Test time (s), Reactive Shuttle Test time (s), ¾ court sprint time (s), number of bench press repetitions at 84 kg (185 lb), vertical jump height (cm), and running vertical jump height (cm) were reported.

### Categorisation and Presentation of Findings

The included physical characteristics were chosen given their importance during basketball match-play [7, 9, 10, 20, 43–45]. The three most frequently used tests for each physical characteristic were selected to represent that characteristic. Anthropometric data pertaining to height, wingspan, mass, and body composition were reported. Muscular power was represented indirectly by three bilateral jumping tests: (1) the countermovement jump (CMJ), which represents the ability to use elastic energy that is generated during a preparatory countermovement, without the influence of the arms (i.e., hands placed on hips); (2) the vertical jump (VJ), which involves both a preparatory countermovement and arm swing; and (3) the squat jump (SJ), which represents the concentric only force expressed during a jump. Linear sprint performances over 5 m, 10 m, and 20 m were reported. Change-of-direction speed tests, which differ from assessments of agility because of their predetermined directional requirements and lack of a perceptual decision-making component [46], included were the Agility *T*-Test, Lane Agility Test, and Y-Shaped change-of-direction Agility Test. Agility tests, which require players to change their movement in response to a stimulus [46, 47], included the Reactive Y-Change-of-Direction Test, the Reactive Change-of-Direction Test, and the Reactive Agility Test. Strength was categorised as lower-body and upper-body strength, using the back squat and bench press, respectively. Only two tests were provided for strength characteristics because of the variability in the remaining tests across studies. Anaerobic capacity was reported using the Wingate Anaerobic Cycle Test (WAnT), full court shuttle run, and the Running-based Anaerobic Speed Test (RAST). Aerobic capacity was reported using tests that assessed maximal oxygen uptake (VO_2max_) or distance covered during a maximal running test. The three tests predominantly used to assess aerobic capacity were the Yo-Yo Intermittent Recovery Test Level 1 (Yo-Yo IRL1), Multi-Stage Fitness Test (MSFT), and incremental treadmill tests.

## Results

### Identification and Selection of Articles

The search of databases identified 1684 studies. A total of 375 duplicates were removed, resulting in 1309 studies to be screened by title and abstract. After screening, 231 studies were eligible for full-text review with a further 59 eligible studies identified in the reference lists during the full-text screening. After full-text screening a total of 137 studies were identified including tests and outcome variables while 114 studies reported physical characteristics in adult male basketball players. Inter-reviewer reliability was calculated using Cohen’s Kappa statistic (*Κ* = 0.85).

### Assessment of Reporting Quality

Reporting quality scores ranged from 6 to 11 for the 12 items assessed in the modified Downs and Black checklist, with a mean score of 9.47 ± 0.83 across the included studies (Table 1 of the ESM).

### Data Collection Methods

The tests and outcome variables used to assess the physical characteristics of adult male basketball players across all competition levels are displayed in Tables 3–10 of the ESM. Tests were categorised based on the characteristic they assessed; body composition, muscular power, linear speed, change-of-direction speed, agility, strength, anaerobic capacity, and aerobic capacity.

### Overview of Included Studies and Tests

A total of 134 tests and 394 outcome variables assessing physical characteristics in adult male basketball players across all competition levels were included in this review. Table 2 summarises tests used across included studies to represent each physical characteristic.

**Table 2:** Tests selected to report the physical characteristics of adult male basketball players in this review

Category	Test	Outcome variable	Citations
Muscular power	Countermovement jump	Peak power and jump height	46
	Squat jump	Peak power and jump height	20
	Vertical jump	Peak power and jump height	33
Linear speed	5-m sprint	Time	10
	10-m sprint	Time	18
	20-m sprint	Time	20
COD speed	Agility *T*-Test	Time	20
	Y-Shaped COD	Time	7
	Lane Agility Test	Time	4
Agility	Y-Shaped Agility Tests	Time, response time, and decision-making time	7
Strength	Bench press	1RM	17
	Back squat	1RM	7
Aerobic capacity	Yo-Yo IRL1	Estimated VO_2max_ and distance	12
	Multi-Stage Fitness Test	Estimated VO_2max_ and number of shuttles	10
	Incremental Treadmill Tests	VO_2max_	21
Anaerobic capacity	Full Court Shuttle Run	Time	5
	RAST	Peak power, mean power, and fatigue index	6
	Wingate Anaerobic Cycle Test	Peak power, mean power, and fatigue index	8

*1RM* one repetition maximum, *COD* change-of-direction, *RAST* Running-Based Anaerobic Speed Test, *VO*_*2max*_ maximum oxygen uptake, *Yo-Yo IRL1* Yo-Yo Intermittent Recovery Test Level 1

### NBA Draft Combine Data Extraction

Data pertaining to the physical characteristics of 100 players drafted into the NBA between the 2011–12 and 2020–21 seasons are presented in Table 3. The mean draft pick number of the players who participated in the NBA Draft Combine increased yearly from 9 ± 4 in 2011–12 to 35 ± 10 in 2020–21. Performance during the Reactive Shuttle Run was only available from the 2013–14 season. Bench press performance was not reported in the 2014–15, 2016–17, and 2020–21 seasons.

**Table 3:** Summary of National Basketball Association Draft Combine performance over the previous 10 years

Test	Mean ± SD	Minimum	Maximum
Height (cm)	197.7 ± 7.6	181.6	212.7
Wingspan (cm)	210 ± 8.6	187.3	231.8
Body mass (kg)	97.2 ± 10.5	77.1	126.4
Body fat %	6.7 ± 1.9	3.2	13.6
Lane Agility Test (s)	11.1 ± 0.4	10.3	12.2
Reactive Shuttle Run (s)	3.0 ± 0.2	2.3	3.7
¾ Court sprint (s)	3.3 ± 0.1	3.6	3.0
Bench press	8.8 ± 4.7	0.0	20.0
Vertical jump (cm)	77.4 ± 7.7	62.2	96.5
Running vertical jump (cm)	92.4 ± 7.9	74.9	110.5
Draft pick	16.6 ± 10.1	2	50

*SD* standard deviation. Bench press = number of completed bench press repetitions at 84 kg (185 lb)

### Anthropometric Characteristics

**Fig. 2:**
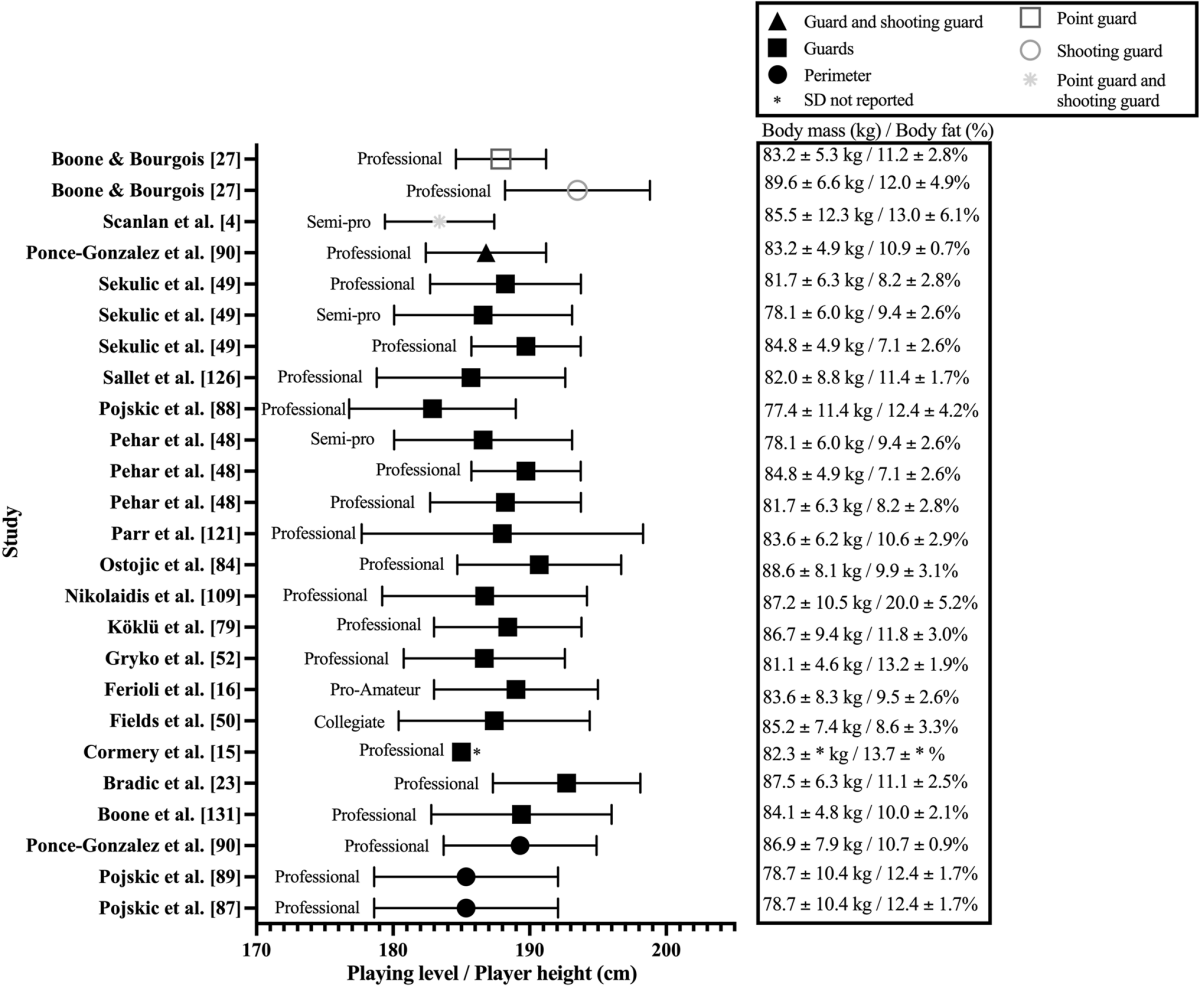
Height, mass, and body fat percentage of the guard playing position in adult male basketball players

**Fig. 3:**
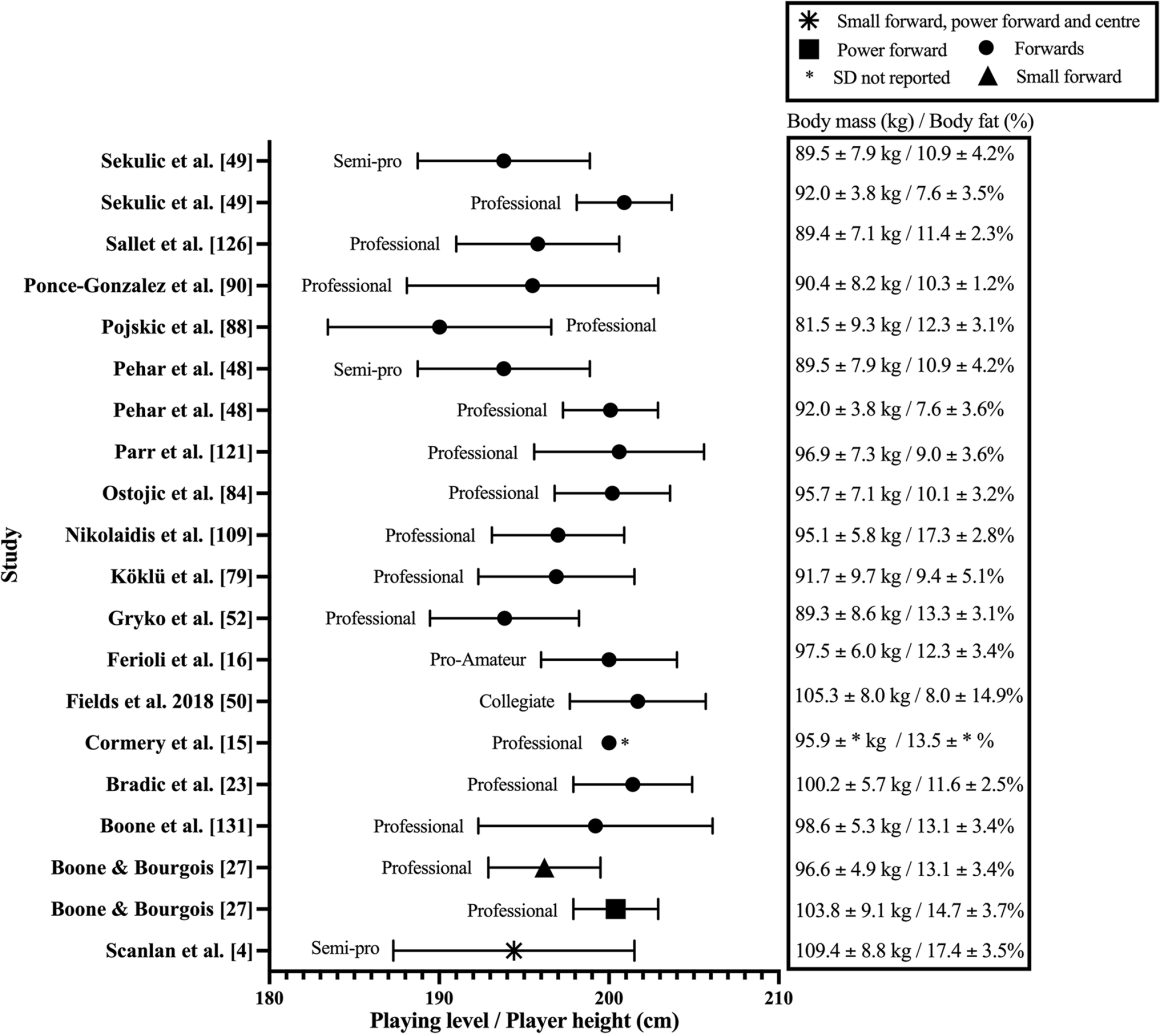
Height, mass, and body fat percentage of the forward playing position in adult male basketball players

**Fig. 4:**
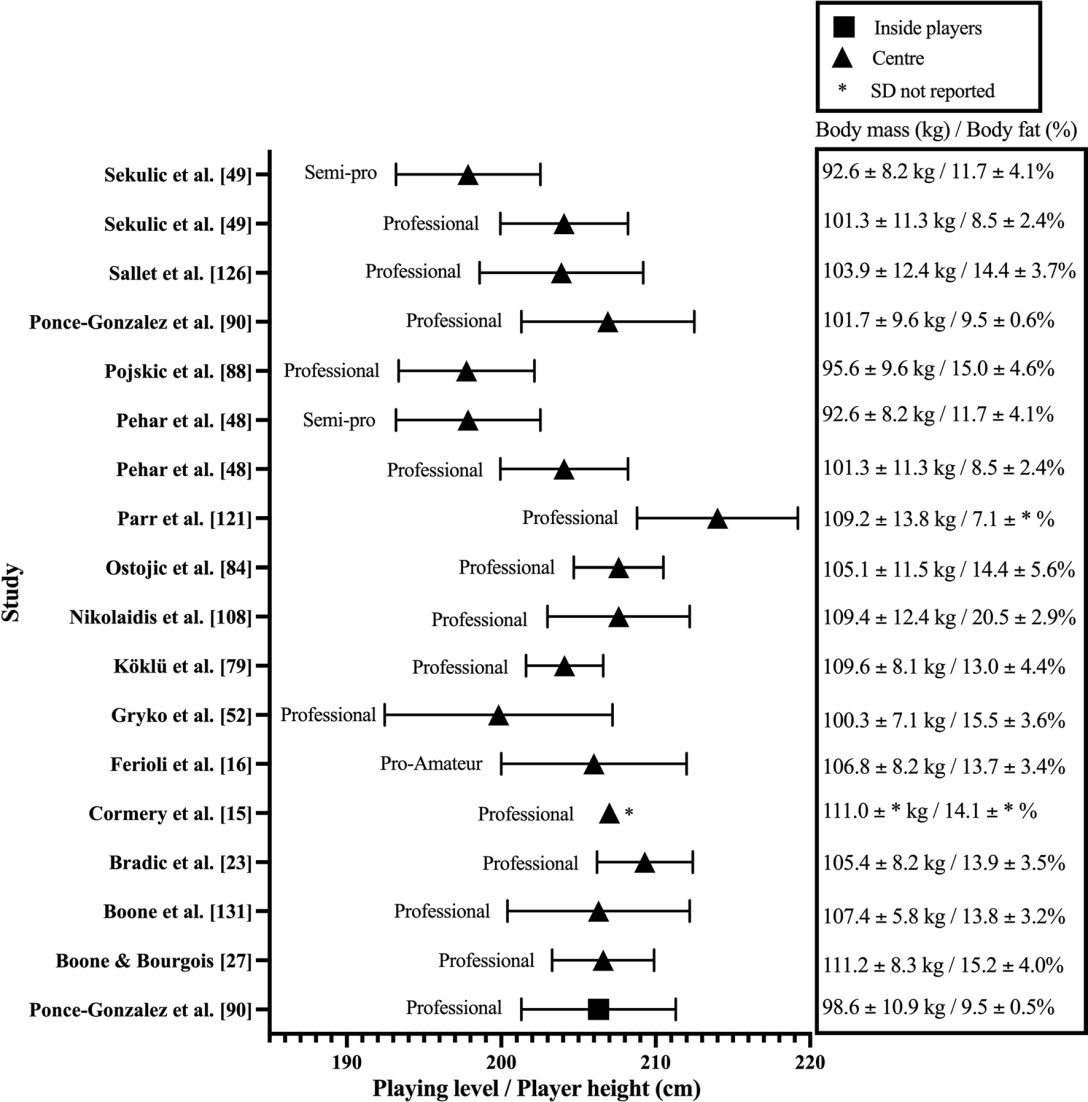
Height, mass, and body fat percentage of the centre playing position in adult male basketball players

Height and body mass were reported in 116 (85%) of the 137 studies included in this review. Anthropometric data (i.e., height, body mass and body fat percentage) were reported according to playing position (Figs. 2, 3, 4) or as a mean across the entire team (Table 2 of the ESM).

Mean height ranged between 177 and 214 cm across studies. The mean height of professional (183–202 cm) and collegiate (177–201 cm) players had similar ranges. Additionally, semi-professional (182–198 cm) and representative (182–197 cm) players had mean heights that were also comparable. Finally, the shortest players were observed at the amateur level with mean height range from 180 to 195 cm. When mean height was reported according to playing position, guards (183–193 cm [Fig. 2]) were consistently reported as being shorter than forwards (190–202 cm [Fig. 3]), with centres observed as the tallest players (198–214 cm [Fig. 4]). Positional mean heights at the professional level followed the same trend (guards: 183–193 cm, forwards: 190–201 cm, and centres 198–214 cm). Furthermore, three studies suggested the same positional trend was present in the mean height of semi-professional players (guards: 183–187 cm [4, 48, 49], forwards: 194 ± 5 cm [48, 49], forwards and centres: 194 ± 7 cm [4], and centres: 198 ± 5 cm [48, 49]). Only two studies (guards: 187 ± 7 cm [50], forwards: 202 ± 4 cm [50], backcourt: 188 ± 6 cm [3], and frontcourt: 201 ± 6 cm [3]) reported collegiate player height according to playing position, while no studies reported height in specific playing positions at the representative or amateur levels.

Mean body mass reported across studies ranged between 68 and 111 kg (Figs. 2, 3, 4 and Table 2 of the ESM). The range in mean body mass of players competing at various competition levels were: professional: 76–105 kg; semi-professional: 74–90 kg; representative: 76–100 kg; collegiate: 69–101 kg; and amateur: 68–94 kg. Observing mean body mass by playing position revealed guards (77–90 kg [Fig. 2]), were typically lightest, with forwards being heavier than guards (82–105 kg [Fig. 3]), and centres being the heaviest (93–111 kg [Fig. 4]). Professional guards had mean body masses between 77 and 90 kg, professional forwards between 82 and 100 kg, and centres between 96 and 111 kg. Three studies reported body mass by playing position at the semi-professional level (guards: 78.1 ± 6 kg [48, 49], 85.5 ± 12.3 kg [4], forwards: 89.5 ± 7.9 kg [48, 49], forwards and centres: 109.4 ± 8.8 kg [4], and centres: 92.6 ± 8.2 kg [48, 49]). Only two studies reported body mass by playing position at the collegiate level (guards: 85.2 ± 7.4 kg [50], forwards: 105.3 ± 8 kg [50], backcourt: 83.3 ± 8.1 kg [3], and frontcourt: 108.1 ± 9.9 kg [3]). No studies reported body mass by playing position at the representative or amateur levels.

Wingspan was reported in three studies [3, 51, 52] with a mean value range from 194 to 207 cm. One study [51] observed a wingspan of 200 ± 10 cm in a team of collegiate NCAA Division 2 players. A second study [3] reported a wingspan of 199 ± 10 cm across the team, with data also provided according to playing position (backcourt: 194 ± 10 cm and frontcourt: 205 ± 3 cm) in collegiate NCAA Division 1 players. Finally, the wingspans of guards (190 ± 5 cm), forwards (197 ± 6 cm), centres (207 ± 8 cm) and the entire team (198 ± 9 cm) were observed in players competing professionally in Poland [52].

Body composition was assessed in 68 (50%) of the 137 studies included in this review, with 14 different types of tests and nine outcome variables used (Table 3 of the ESM). Data pertaining to body composition by playing position are reported in Fig. 2 (guards), Fig. 3 (forwards) and Fig. 4 (centres), and mean team measurements are provided in Table 2 of the ESM. The most frequently implemented test and outcome variable used across studies were the sum of skinfolds at three sites (chest, abdomen, and thigh [16, 53–58], triceps, pectoral, and subscapular [59], and triceps, abdomen, and thigh [4, 21, 60]) and body fat percentage, respectively (Table 3 of the ESM).

Mean body composition ranged between 7 and 24% body fat across studies (Figs. 2, 3, 4 and Table 2 of the ESM). Mean body fat percentage across competition levels revealed professional players varied between 7 and 20%, while semi-professional (9–16%), collegiate (10–14%), and representative (8–14%) levels exhibited similar ranges in body fat percentage. Amateur players possessed varied mean body composition measurements of between 10 and 24% body fat. When mean body composition was reported according to playing position, guards (7–20% [Fig. 2]), forwards (8–17% [Fig. 3]), and centres (7–21% [Fig. 4]) demonstrated similar variance in body fat percentage. Professional guards (7–20%), forwards (8–17%), and centres (7–21%) also possessed similar levels of body fat. Semi-professional guard or backcourt (9–13%) and forward or frontcourt (11–17%) body fat percentages were reported in three studies [4, 48, 49], whereas centres (11.7 ± 4.1%) were only reported in two studies [48, 49]. Body fat percentages were reported for guard and forward positions at the collegiate level in one study [50] (Figs. 2, 3). No studies reported body fat percentage relative to playing position at the representative or amateur playing levels.

### Muscular Power

Muscular power was assessed predominantly using jump tests, with 80 (58%) of the 137 studies in this review employing 18 different jump tests (Table 4 of the ESM). The three most frequent jump tests adopted across studies were the CMJ (43 studies, 54% of studies assessing muscular power) [14, 16, 27, 48, 51, 54, 55, 58, 61–75,77–96], VJ (27 studies, 34% of studies assessing muscular power) [3, 14, 28, 39, 56, 59, 62, 66, 82, 90–92, 96–100, 102–111], and SJ (15 studies, 18% of studies assessing muscular power) [27, 58, 61, 63, 67, 72, 77–79, 81, 89, 90, 93, 95, 96]. Additional jump tests used across studies are reported in Table 4 of the ESM. The most commonly reported outcome variables were jump height and peak power (Table 4 of the ESM). Two throwing tests were also used in studies to assess muscular power including a seated basketball throw [68] with speed (km/hour) of the throw taken as the outcome variable, and a seated medicine ball throw (1 kg [87, 89] and unknown mass [56]) with horizontal displacement (m) of the ball used as the outcome variable. Additionally, muscular power variables were also recorded during tests predominantly implemented to assess strength. These tests are reported in Sect. 3.2.7 and include bench press and squat exercises (Table 8 of the ESM).

Jump performance variables reported across studies during the CMJ, VJ, and SJ are provided in Tables 4, 5, and 6. Mean CMJ height ranged between 34 and 77 cm, while mean peak power outputs ranged between 2441 and 6647 W (Table 4). In professional players, mean CMJ height and mean CMJ peak power ranged between 36 and 63 cm and between 3874 and 5468 W, respectively. Mean CMJ height (34–50 cm) and mean CMJ peak power (2441–5078 W) were lower in semi-professional players than professional players, while collegiate players had the greatest mean jump height (36–77 cm) and peak power output (4736–6647 W). Countermovement jump height was only reported in one study [65] at the representative level with CMJ peak power not reported. Countermovement jump height and CMJ peak power were reported at the amateur level in one study [16] (Table 4). Countermovement jump performance was only reported according to playing position at the professional level [14, 27, 48, 79, 84, 88, 90], the collegiate level [75] and as a combined group of players from amateur to professional levels [16]. Similar mean CMJ heights were evident between positions in professional players (guards: 38–60 cm, forwards: 36–58 cm, and centres: 36–57 cm), while mean absolute peak power was lowest in guards (3874–4510 W), then forwards (3930–5221 W), and greatest in centres (4536–5353 W).

**Table 4:** Jump height and peak power variables reported during the countermovement jump in adult male basketball players

Study	Playing position	Competition level	Category	Jump height (cm)	Peak power (W)
Alemdaroglu [61]	All	Turkish D1	Professional	34.9 ± 3.8	
Annino et al. [62]	All	Italian National Federal League L2	Professional	38.9 ± 3.6	
Aoki et al. [63]	All	Brazilian National League	Professional	38.1 ± 2.8	
Barrera-Domínguez et al. [64]	All	Spanish National Division	Professional	35.6 ± 4.8	
Ben Abdelkrim et al. [65]	All (U20) All	Tunisian National Team Tunisian National Team	Representative Professional	49.1 ± 5.9 49.7 ± 5.8	4656 ± 81 4665 ± 116
Boone and Bourgois [27]	Point guard Shooting guard Small forward Power forward Centre	Belgian D1 Belgian D1 Belgian D1 Belgian D1 Belgian D1	Professional Professional Professional Professional Professional	42.7 ± 3.8 41.3 ± 3.2 42.5 ± 3.8 42.4 ± 3.7 36.2 ± 4.1	4306 ± 373 4510 ± 322 4901 ± 387 5221 ± 364 5180 ± 451
Buśko et al. [66]	All	Warsaw Sports Club Polonia D2	Semi-professional	41.9 ± 4.0	2441 ± 440
Chaouachi et al. [67]	All	Tunisian National Team	Professional	61.9 ± 6.2	
Chen et al. [68]	All	Collegiate D1	Collegiate	45.6 ± 4.0	
Ciacci and Bartolomei [96]	All All	National Level National Level	Professional Professional	39.2 ± 5.7 41.9 ± 5.2	
Dawes and Spiteri [51]	All	NCAA D2	Collegiate	76.9 ± 7.5	
Dello Iacono et al. [69]	All	Professional Basketball Club UK	Professional	60.4 ± NR	
Ferioli et al. [54]	All All	Italian Serie A and Serie A2 Italian Serie B	Professional Semi-professional	50.3 ± 5.4 49.4 ± 5.4	5153 ± 593 4405 ± 667
Ferioli et al. [16]	All All All All Guard Forward Centre	Italian Serie A Italian Serie A2 Italian Serie B Italian Serie D Italian Serie A-D Italian Serie A-D Italian Serie A-D	Professional Professional Semi-professional Amateur Amateur – Pro Amateur – Pro Amateur – Pro	47.8 ± 5.7 49.2 ± 4.9 48.0 ± 6.1 51.8 ± 4.1 49.2 ± 4.9 48.6 ± 6.0 45.8 ± 6.0	5468 ± 820 5177 ± 629 4685 ± 723 4800 ± 536 4785 ± 678 5436 ± 738 5560 ± 682
Ferioli et al. [55]	All All All	Italian Serie A Italian Serie A2 Italian Serie B	Professional Professional Semi-professional	46.9 ± 4.4 50.9 ± 5.6 50.1 ± 4.8	5282 ± 582 5182 ± 745 4691 ± 624
Freitas et al. [70]	All	Spanish Liga EBA D4	Semi-professional	35.0 ± 7.0	5078 ± 437
Freitas et al. [71]	All	Spanish Liga EBA D4	Semi-professional	36.5 ± 7.2	4699 ± 781
Gomes et al. [72]	All	PSCB	Professional	39.3 ± 5.6	
Heishman et al. [74]	All	NCAA D1	Collegiate	58.3 ± 1.4	6374 ± 165
Heishman et al. [73]	All	NCAA D1	Collegiate	62.8 ± 1.5	6647 ± 171
Heishman et al. [75]	All Guards Frontcourt	NCAA D1 NCAA D1 NCAA D1	Collegiate Collegiate Collegiate	38.7 ± 6.4 42.6 ± 0.4 34.6 ± 0.4	
Jallai et al. [77]	All	Estonian 1^st^ League	Professional	43.2 ± 2.3	
Khlifa et al. [78]	All	Tunisian D1	Professional	45.2 ± 1.3^#^	
Köklü et al. [79]	All All All Guard Forward Centre	Turkish D1 and D2 Turkish D1 Turkish D2 Turkish D1 and D2 Turkish D1 and D2 Turkish D1 and D2	Professional Professional Professional Professional Professional Professional	38.3 ± 5.3 40.6 ± 4.7 36.0 ± 5.0 38.2 ± 5.8 40.1 ± 5.1 36.6 ± 4.7	
Laplaud et al. [80]	All	Professional	Professional	63.0 ± 9.0	
Maffiuletti et al. [81]	All	French Basketball Federation D2	Professional	51.0 ± 1.3	
Maggioni et al. [58]	All	Volunteer Players	Semi-professional	32.0 ± 4.9	
Mandic et al. [82]	All	National League Serbia	Professional	36.9 ± 3.7	
Miura et al. [83]	All	National Collegiate Japan	Collegiate	50.5 ± 5.4	
Ostojic et al. [84]	All Guard Forward Centre	First National League Serbia First National League Serbia First National League Serbia First National League Serbia	Professional Professional Professional Professional	57.4 ± 7.7 59.7 ± 9.6 57.8 ± 6.5 54.6 ± 6.9	
Pehar et al. [48]	All All Guard Forward Centre Guard Guard Forward Forward Centre Centre	Bosnia and Herzegovina D1 Bosnia and Herzegovina D2 Bosnia and Herzegovina D1 and D2 Bosnia and Herzegovina D1 and D2 Bosnia and Herzegovina D1 and D2 Bosnia and Herzegovina D1 Bosnia and Herzegovina D2 Bosnia and Herzegovina D1 Bosnia and Herzegovina D2 Bosnia and Herzegovina D1 Bosnia and Herzegovina D2	Professional Professional Professional Professional Professional Professional Professional Professional Professional Professional Professional	45.5 ± 5.6 45.3 ± 6.1 46.4 ± 6.0 45.5 ± 5.5 43.9 ± 5.5 46.9 ± 5.4 46.0 ± 6.6 46.5 ± 5.3 44.5 ± 5.9 43.7 ± 5.6 44.5 ± 5.6	
Pehar et al. [85]	All	Bosnia and Herzegovina D1	Professional	45.6 ± 5.5	
Pliauga et al. [86]	All	Lithuanian National Basketball League	Collegiate	47.8 ± 3.0	
Pojskić et al. [88]	Guard	Bosnian Premier League	Professional	40.4 ± 5.0	3874 ± 639
	Forward	Bosnian Premier League	Professional	37.6 ± 6.8	3930 ± 604
	Centre	Bosnian Premier League	Professional	36.0 ± 3.8	4536 ± 458
Pojskić et al. [87]	Perimeter	Bosnian Premier League	Professional	38.5 ± 3.5	
Pojskić et al. [89]	Perimeter	Bosnian and Herzegovina D1	Professional	38.5 ± 3.5	
Ponce-González et al. [90]	All Guard Forward Centre Perimeter Inside	Spanish Pro Liga ACB Spanish Pro Liga ACB Spanish Pro Liga ACB Spanish Pro Liga ACB Spanish Pro Liga ACB Spanish Pro Liga ACB	Professional Professional Professional Professional Professional Professional	36.8 ± 4.1 37.7 ± 3.8 35.6 ± 4.6 37.2 ± 4.9 37.4 ± 3.8 36.1 ± 4.9	4707 ± 676 4159 ± 218 4607 ± 606 5353 ± 538 4399 ± 521 5137 ± 672
Puente et al. [14]	All	National Spanish Basketball Federation	Professional	58 ± 4	
	Guard	National Spanish Basketball Federation	Professional Professional	58 ± 3 58 ± 5	
	Forward	National Spanish Basketball Federation			
	Centre	National Spanish Basketball Federation	Professional	57 ± 2	
Rodriguez-Rosell et al. [91]	All	Spanish Liga EBA D4	Semi-professional	34.8 ± 5.8	
Schiltz et al. [92]	All	European Cup D1	Professional	40.5 ± 5.7	
Shalfawi et al. [93]	All	Professional level Norway	Professional	52.0 ± 7.5	5167 ± 419
Stojanovic et al. [94]	All	Serbian Professional League	Professional	39.8 ± 5.1	
Xie et al. [95]	All All	NCAA D1 university team NCAA D1 club team	Collegiate Collegiate	71.9 ± 9.1 72.7 ± 4.3	

Data are presented as mean ± standard deviation

*D1* Division one competition, *D2* Division two competition, *D4* Division four competition, *Inside* power forward and centre positions, *L2* second league, *Liga ACB* Liga Endesa Asociación de Clubs de Baloncesto, *Liga EBA* Liga Española de Baloncesto Aficionado, *NCAA* National Collegiate Athletic Association, *NR* no data provided, *Perimeter* point guard, shooting guard and small forward positions, *PSBC* Paulista State Basketball Championships, *U20* players competing in an under 20 years of age competition, # indicates standard error of measurement

**Table 5:** Jump height and peak power variables reported during the vertical jump in adult male basketball players

Study	Playing position	Competition level	Category	Jump height (cm)	Peak power (W)
Annino et al. [62]	All	Italian National Federal League L2	Professional	45.9 ± 3.4	
Asadi et al. [97]	All	Provincial D1 Italy	Amateur	41.3 ± 3.4	
Balabinis et al. [98]	All	Undergraduate Teams Greece	Amateur	52.2 ± 2.2	
Balsalobre-Fernandez et al. [99]	All	Spanish Pro Liga ACB	Professional	45.6 ± 5.9	
Balsalobre-Fernandez et al. [100]	All	Spanish Pro Liga ACB	Professional	43.9 ± 7.3	4856 ± 601^WPD^
Buśko et al. [66]	All	Warsaw Sports Club Polonia D2	Semi-professional	52.5 ± 5.1	3591 ± 788
Ciacci and Bartolomei [96]	All All	National Level Italy National Level Italy	Professional Professional	44.6 ± 6.2 49.3 ± 5.7	
de Sousa Fortes et al. [59]	All	State Basketball Championship Brazil	Professional	42.0 ± 8.0	
Hoffman et al. [103]	All	NCAA D1	Collegiate	64.5 ± 9.7	
Hoffman et al. [102]	All	NCAA D1	Collegiate	63.4 ± 6.9	
Hoffman et al. [104]	All 88–89 All 89 –90 All 90–91 All 91–92	NCAA D1 NCAA D1 NCAA D1 NCAA D1	Collegiate Collegiate Collegiate Collegiate	68.1 ± 8.6 66.0 ± 6.9 72.6 ± 5.6 67.3 ± 6.0	
Hunter et al. [105]	All	U.S College	Collegiate	61.0 ± 7.4	
Kariyawasam et al. [56]	All	National Level Sri Lanka	Professional	47.9 ± 6.8	
Kipp et al. [106]	All	NCAA D1	Collegiate	62.4 ± 5.4	
Korkmaz and Karahan [107]	All All All	Turkish D1 Turkish D2 Turkish D3 (regional)	Professional Professional Semi-professional	48.2 ± 4.0 48.3 ± 3.0 45.5 ± 4.0	2347 ± 161 2215 ± 130 2121 ± 130
Lehnert et al. [108]	All	Czech First League	Professional	48.2 ± 4.6	
Lockie et al. [3]	All Backcourt Frontcourt	NCAA D1 NCAA D1 NCAA D1	Collegiate Collegiate Collegiate	77.9 ± 9.9 83.4 ± 8.4 69.9 ± 5.2	
Mandic et al. [82]	All	National League Serbia	Professional	43.3 ± 3.6	
Montgomery et al. [28]	All Guard Forward Centre	Australian State Level Australian State Level Australian State Level Australian State Level	Semi-professional Semi-professional Semi-professional Semi-professional	61.9 ± 14.6 61.3 ± 19.9 61.2 ± 7.5 65.3 ± 9.0	
Nikolaidis et al. [109]	All Guard Forward Centre	Italian First League Italian First League Italian First League Italian First League	Professional Professional Professional Professional	44.4 ± 6.8 45.1 ± 3.3 46.9 ± 7.8 39.0 ± 4.3	
Pliaugu et al. [39]	All	Lithuania National Basketball League	Collegiate	53.0 ± 9.5^WPD^	
Ponce-González et al. [90]	All Guard Forward Centre Perimeter Inside	Spanish Pro Liga ACB Spanish Pro Liga ACB Spanish Pro Liga ACB Spanish Pro Liga ACB Spanish Pro Liga ACB Spanish Pro Liga ACB	Professional Professional Professional Professional Professional Professional	44.2 ± 4.4 43.7 ± 1.8 43.9 ± 4.3 45.0 ± 7.0 44.4 ± 2.8 43.9 ± 6.5	5753 ± 932 4992 ± 546 5567 ± 606 6701 ± 709 5300 ± 663 6388 ± 931
Puente et al. [14]	All	National Spanish Basketball Federation	Professional	64 ± 4	
	Guard	National Spanish Basketball Federation	Professional	65 ± 3	
	Forward	National Spanish Basketball Federation	Professional	64 ± 6	
	Centre	National Spanish Basketball Federation	Professional	63 ± 4	
Rauch et al. [110]	All	NBA	Professional	68.7 ± 7.4	
Rodriguez-Rosell et al. [91]	All	Spanish Liga EBA D4	Semi-professional	40.5 ± 7.0	
Schiltz et al. [92]	All	Professional D1 European Cup	Professional	48.7 ± 5.3	
Townsend et al. [111]	All	Collegiate D1 United States	Collegiate	77.4 ± 6.4	

Data are presented as mean ± standard deviation

88–89 data collected from collegiate team during the 1988–90 season, 89–90 data collected from collegiate team during the 1989–90 season, 90–91 data collected from collegiate team during the 1990–91 season, 91–92 data collected from collegiate team during the 1991–2 season, *Backcourt players* guards, *D1* Division one competition, *D2* Division two competition, *D3* Division three competition, *D4* Division four competition, *Inside* power forward and centre positions, *Frontcourt players* forwards and centres, *L2* second league, *Liga ACB* Liga Endesa Asociación de Clubs de Baloncesto, *Liga EBA* Liga Española de Baloncesto Aficionado, *NBA* National Basketball Association, *NCAA* National Collegiate Athletic Association, *NR* no data provided, *Perimeter* point guard, shooting guard and small forward positions, ^WPD^ indicates data extracted by WebPlotDigitizer

**Table 6:** Jump height and power variables according to playing position and competition level during the squat jump in adult male basketball players

Study	Playing position	Competition level	Category	Jump height (cm)	Peak power (W)
Alemdaroglu [61]	All	Turkish D1	Professional	32.9 ± 3.8	
Aoki et al. [63]	All	Brazilian National League	Professional	34.8 ± 2.6	
Boone and Bourgois [27]	Point guard Shooting guard Small forward Power forward Centre	Belgian D1 Belgian D1 Belgian D1 Belgian D1 Belgian D1	Professional Professional Professional Professional Professional	41.0 ± 3.8 39.5 ± 3.6 40.2 ± 3.7 39.1 ± 4.2 35.7 ± 3.2	4203 ± 371 4402 ± 358 4761 ± 381 5021 ± 423 5149 ± 399
Chaouachi et al. [67]	All	Tunisian National Team	Professional	49.5 ± 4.8	
Ciacci and Bartolomei [96]	All All	National Level Italy National Level Italy	Professional Professional	36.2 ± 5.0 42.5 ± 5.0	
Gomes et al. [72]	All	PSBC	Professional	33.4 ± 5.2	
Jallai et al. [77]	All	Estonian 1st League	Professional	40.4 ± 2.0	24.23 ± 5.77^
Khlifa et al. [78]	All	Tunisian D1	Professional	38.6 ± 1.1^#^	
Köklü et al. [79]	All All All Guard Forward Centre	Turkish D1 and D2 Turkish D1 Turkish D2 Turkish D1 and D2 Turkish D1 and D2 Turkish D1 and D2	Professional Professional Professional Professional Professional Professional	36.2 ± 5.5 37.8 ± 5.7 34.7 ± 5.7 36.4 ± 5.7 37.7 ± 5.2 34.7 ± 5.4	
Maffiuletti et al. [81]	All	French Basketball Federation D2	Professional	44.1 ± 1.8	
Maggioni et al. [58]	All	Volunteer Players	Semi-professional	26.5 ± 3.8	
Pojskic et al. [89]	All	Bosnia and Herzegovina D1	Professional	31.1 ± 31.1	
Ponce-Gonzalez et al. [90]	All Guard Forward Centre Perimeter Inside	Spanish Pro Liga ACB Spanish Pro Liga ACB Spanish Pro Liga ACB Spanish Pro Liga ACB Spanish Pro Liga ACB Spanish Pro Liga ACB	Professional Professional Professional Professional Professional Professional	30.6 ± 5.5 30.1 ± 5.7 28.5 ± 3.2 33.2 ± 7.3 29.6 ± 4.6 31.9 ± 6.8	4242 ± 746 3639 ± 413 4034 ± 390 5054 ± 544 3872 ± 449 4761 ± 807
Shalfawi et al. [93]	All	Professional level Norway	Professional	43.1 ± 7.2	4609 ± 419
Xie et al. [95]	All All	NCAA D1 university team NCAA D1 club team	Collegiate Collegiate	57.8 ± 7.9 56.4 ± 3.7	

Data are presented as mean ± standard deviation

*D1* Division one competition, *D2* Division two competition, *Inside* power forward and centre positions, *Liga ACB* Liga Endesa Asociación de Clubs de Baloncesto, *NCAA* National Collegiate Athletic Association, *Perimeter* point guard, shooting guard and small forward positions, *PSBC* Paulista State Basketball Championships, ^ indicates reported in W⋅kg^−1^, # indicates standard error of measurement

Mean jump height measured during the VJ was the greatest across all jump tests, with a range from 39 to 83 cm, while mean VJ peak power ranged between 2121 and 6701 W (Table 5). Professional players recorded mean VJ heights between 39 and 69 cm and mean VJ peak power outputs of 2215–6701 W during the VJ. Semi-professional players reached mean VJ heights between 41 and 65 cm. However, only two studies [66, 107] reported peak power output (2121–3591 W) for semi-professional players during the VJ. Collegiate players recorded mean VJ heights between 44 and 83 cm, while no studies reported peak power output. No studies reported VJ performance in representative players. Two studies [97, 98] reported VJ height in amateur players (41–52 cm), while no studies reported peak power output in amateur players. Vertical jump height relative to playing position (guards: 44–65 cm, forwards: 44–64 cm, and centres: 39–63 cm) was only reported in professional players across three studies [14, 90, 109], one of which measured peak power [90] (Table 5). Positional VJ performance was only reported in one study in semi-professional [28] and collegiate [3] players (Table 5). No studies reported positional VJ performance in representative or amateur players.

Mean SJ height ranged between 27 and 58 cm, while mean SJ peak power outputs were only reported in professional players and ranged between 3639 and 5149 W (Table 6). Professional players reached mean SJ jump heights between 29 and 50 cm. Only one study [58] reported SJ height in semi-professional players, while peak power was not reported. Squat jump height in collegiate players was only reported in one study [95], while no studies reported SJ peak power output in collegiate players. No studies reported SJ height or peak power in representative or amateur players. Mean SJ height [27, 79, 90] (guards: 30–41 cm, forwards: 29–40 cm, and centres: 33–36 cm) and peak power [27, 90] (guards: 3639–4402 W, forwards: 4034–5021 W, and centres: 5054–5149 W) were only reported according to playing position at the professional level. Reliability statistics for each of the jump tests reported in Tables 4, 5 and 6 are provided in Table 13 of the ESM.

### Linear Sprint Speed

Linear sprint tests were conducted in 39 (28%) of the 137 studies included in this review (Table 5 of the ESM). The most frequently included linear sprint distances were 5 m (9 studies, 23% of studies assessing linear sprint speed) [4, 21, 27, 51, 58, 64, 65, 67, 112], 10 m (16 studies, 41% of studies assessing linear sprint speed) [21, 27, 60, 61, 64, 65, 67, 71, 79, 86, 91, 93, 95, 112, 113] and 20 m (18 studies, 46% of studies assessing linear sprint speed) [4, 21, 28–30, 39, 51, 58, 59, 72, 87, 89, 91, 93, 95, 107, 111, 114]. Time was the most common outcome variable and was used in every linear sprint test adopted across studies (Table 5 of the ESM).

Over 5 m, mean sprint times ranged from 0.80 to 1.51 s (Table 7). Professional players demonstrated mean 5-m sprint times between 0.82 and 1.51 s (Table 7). Only four studies [4, 21, 58, 112] reported 5-m sprint times at the semi-professional level (1.04–1.14 s). One study reported 5-m sprint time in representative [65] and collegiate [51] players (Table 7). No studies reported 5-m sprint times in amateur players. Mean sprint times over 10 m ranged from 1.47 to 2.34 s (Table 7). Professional players recorded mean 10-m sprint times between 1.47 and 2.34 s. Homogenous mean 10-m sprint times were reported in semi-professional players (1.77–1.90 s). Only one study [113] reported 10-m sprint time in amateur players (Table 7). Mean sprint times over 20 m ranged between 2.43 and 3.36 s (Table 7). Mean sprint times over 20 m were similar between professional (2.43–3.24 s) and semi-professional (2.80–3.24 s) players. One study [114] reported 20-m sprint times in representative players (Table 7). Collegiate players recorded mean 20-m sprint times ranging from 2.80 to 3.36 s. No studies reported 20-m sprint time in amateur players. Linear sprint performance according to playing position was reported at the professional [27] and semi-professional [4] levels across 5 m, professional [27, 79] and semi-professional [4] levels across 10 m, and professional [87, 89] and semi-professional [4, 28] levels across 20 m (Table 7). Reliability statistics for each of the linear sprint tests described in Table 7 are provided in Table 13 of the ESM.

**Table 7:** Sprint times recorded during 5-m, 10-m and 20-m linear sprints in adult male basketball players

Study	Playing position	Competition level	Category	5-m sprint time (s)	10-m sprint time (s)	20-m sprint time (s)
Alemdaroglu [61]	All	Turkish D1	Professional		1.86 ± 0.30	
Barrera-Domínguez et al. [64]	All	Spanish National Division	Professional	0.88 ± 0.04	1.47 ± 0.08	
Ben Abdelkrim et al. [65]	All (U20)	Tunisian National Team	Representative	1.00 ± 0.10		
	All	Tunisian National Team	Professional	1.04 ± 0.16	1.84 ± 0.10 1.88 ± 0.15	
Boone and Bourgois [27]	Point guard	Belgian D1	Professional	1.40 ± 0.03	2.16 ± 0.09	
	Shooting guard	Belgian D1	Professional	1.40 ± 0.09	2.19 ± 0.08	
	Small forward	Belgian D1	Professional	1.45 ± 0.09	2.23 ± 0.09	
	Power forward	Belgian D1	Professional	1.47 ± 0.08	2.25 ± 0.08	
	Centre	Belgian D1	Professional	1.51 ± 0.07	2.34 ± 0.11	
Chaouachi et al. [67]	All	Tunisian National Team	Professional	0.82 ± 0.05	1.70 ± 0.06	
Dawes and Spiteri [51]	All	NCAA D2	Collegiate	0.80 ± 0.04		2.80 ± 0.08
de Sousa Fortes et al. [59]	All	State Basketball Championship Brazil	Professional			2.43 ± 0.21
Delextrat and Cohen [114]	All All All	BUSA D1 and D2 BUSA D1 BUSA D2	Collegiate—Representative Representative Collegiate			3.33 ± 0.26 3.29 ± 0.12 3.36 ± 0.36
Freitas et al. [71]	All	Spanish Liga EBA D4	Semi-professional		1.91 ± 0.09	
Gomes et al. [72]	All	PSBC	Professional			3.24 ± 0.22
Köklü et al. [79]	All	Turkish D1 and D2	Professional		1.75 ± 0.08	
	All	Turkish D1	Professional		1.78 ± 0.80	
	All	Turkish D2	Professional		1.72 ± 0.80	
	Guards	Turkish D1 and D2	Professional		1.72 ± 0.07	
	Forward	Turkish D1 and D2	Professional		1.72 ± 0.07	
	Centre	Turkish D1 and D2	Professional		1.80 ± 0.08	
Korkmaz and Karahan [107]	All	Turkish D1	Professional			2.70 ± 0.14
	All	Turkish D2	Professional			2.80 ± 0.10
	All	Turkish D3	Semi-professional			2.80 ± 0.13
Lockie et al. [113]	All	Australian State level	Semi-professional		1.81 ± 0.09	
	All	Recreational Australia	Amateur		1.88 ± 0.07	
Maggioni et al. [58]	All	Volunteer Players	Semi-professional	1.04 ± 0.05	1.77 ± 0.04	3.10 ± 0.12
Montgomery et al. [28]	All	Australian State Level	Semi-professional			3.09 ± 0.10
	Guard	Australian State Level	Semi-professional			3.04 ± 0.07
	Forward	Australian State Level	Semi-professional			3.13 ± 0.13
	Centre	Australian State Level	Semi-professional			3.10 ± 0.09
Pliauga et al. [39]	All	Lithuanian National Basketball League	Collegiate			2.98 ± 0.32
Pliauga et al. [86]	All	Lithuanian National Basketball League	Collegiate		1.80 ± 0.03	
Pojskić et al. [87]	Perimeter	Bosnia and Herzegovina D1	Professional			3.14 ± 0.09
Pojskić et al. [89]	Perimeter	Bosnian Premier League	Professional			3.14 ± 0.09
Poole et al. [112]	All	Australian State level QBL	Semi-professional	1.14 ± 0.07	1.90 ± 0.10	
Ramirez-Campillo et al. [29]	All	SCCSBL	Collegiate			3.00 ± 0.11
Rodriguez-Rosell et al. [91]	All	Spanish Liga EBA D4	Semi-professional		1.84 ± 0.10	3.20 ± 0.16
Scanlan et al. [21]	All	Australian State Level	Semi-professional	1.07 ± 0.07	1.83 ± 0.10	3.16 ± 0.19
Scanlan et al. [4]	Backcourt	Australian State Level	Semi-professional	1.05 ± 0.03	1.78 ± 0.05	3.08 ± 0.12
	Frontcourt	Australian State Level	Semi-professional	1.10 ± 0.09	1.87 ± 0.13	
Scanlan et al. [30]	All	Australian State Level	Semi-professional			3.24 ± 0.22 2.97 ± 0.06
Shalfawi et al. [93]	All	Professional Level Norway	Professional		1.88 ± 0.21	3.20 ± 0.33
Townsend et al. [111]	All	Collegiate D1 United States	Collegiate			3.31 ± 0.15^1080^
Xie et al. [95]	All	NCAA D1 university and club teams	Collegiate		5.67 ± 0.17^ m/s^	7.94 ± 0.45^ m/s^

Data presented as mean ± standard deviation

*BUSA* British Universities Sports Association, *D1* Division one competition, *D2* Division two competition, *D3* Division three competition, *D4* Division four competition, *Liga EBA* Liga Española de Baloncesto Aficionado, *NCAA* National Collegiate Athletic Association, *Perimeter* point guard, shooting guard and small forward positions, *PSBC* Paulista State Basketball Championships, *SCCSBL* South Chilean College System Basketball League, *U20* players competing in an under 20 years of age competition, ^m/s^ indicates velocity in m^.^s^−1^, ^1080^ indicates performed using 1080 sprint with 1-kg resistance

### Change-of-Direction Speed

Change-of-direction speed was assessed in 38 (28%) of the 137 studies in this review, with 17 different tests used (Table 6 of the ESM). All tests used time as the primary outcome variable except the Multi-Stage Change of Direction Exercise Test, which used metabolic power, running speed, peak torque and fatigue index as outcome variables (Table 6 of the ESM). The Agility *T*-Test was the most frequently implemented change-of-direction speed test being used in 20 studies (53% of studies measuring change-of-direction speed) [49, 58, 61, 65, 67, 70–72, 79, 87, 89, 97, 102–104, 112, 114–117]. Mean Agility *T*-Test time ranged between 8.84 and 10.90 s across studies. Professional players demonstrated mean Agility *T*-Test times between 8.84 and 10.04 s, which were similar to collegiate players (8.92–9.78 s), and quicker than mean times reported in semi-professional players (9.52–10.90 s). Agility *T*-Test time was only reported in representative players (9.21–10.05 s) in two studies [65, 114] while only one study [97] observed amateur players (Table 8). Two studies [49, 79] reported Agility *T*-Test times relative to playing position (guard: 8.96–9.24 s, forward: 8.84–9.48 s, and centre: 9.73–10.04 s), only at the professional level. The Lane Agility Test was only used in three studies (8% of studies measuring change-of-direction speed) [3, 51, 111] and only assessing collegiate players (10.16–11.80 s), with one study [3] reporting results according to playing position (Table 8). Table 8 contains Agility *T*-Test, Lane Agility Test and Y-Shaped Change-of-Direction Speed Test outcomes reported in adult male basketball players. Reliability statistics for each of the change-of-direction tests described in Table 8 are provided in Table 13 of the ESM.

**Table 8:** COD speed test performance according to playing position and competition level in adult male basketball players

Study	Playing position	Competition level	Category	Test	Time (s)
Alembaroglu [61]	All	Turkish D1	Professional	Agility *T*-Test	9.25 ± 0.46
Asadi et al. [97]	All	Provincial D1 Italy	Amateur	Agility *T*-Test	12.00 ± 0.56
Barrera-Domínguez et al. [64]	All	Spanish National Division	Professional	Modified *T*-Test	6.49 ± 0.34
Ben Abdelkrim et al. [65]	All (U20)	Tunisian National Team	Representative	Agility *T*-Test	10.05 ± 0.44
	All	Tunisian National Team	Professional	Agility *T*-Test	9.99 ± 0.40
Chaouachi et al. [67]	All	Tunisian National Team	Professional	Agility *T*-Test	9.70 ± 0.20
Delextrat and Cohen [114]	All	BUSA D1 and D2	Collegiate – Representative	Agility *T*-Test	9.49 ± 0.56
	All	BUSA D1	Representative	Agility *T*-Test	9.21 ± 0.24
	All	BUSA D2	Collegiate	Agility *T*-Test	9.78 ± 0.59
Freitas et al. [70]	All	Spanish Liga EBA D4	Semi-professional	Agility *T*-Test	9.52 ± 0.63
Freitas et al. [71]	All	Spanish Liga EBA D4	Semi-professional	Agility *T*-Test	9.71 ± 0.67
Gomes et al. [72]	All	PSBC	Professional	Agility *T*-Test	9.28 ± 0.46
Hoffman et al. [102]	All	NCAA D1	Collegiate	Agility *T*-Test	8.92 ± 0.30
Hoffman et al. [103]	All	NCAA D1	Collegiate	Agility *T*-Test	9.18 ± 0.54
Hoffman et al. [104]	All 88–89	NCAA D1	Collegiate	Agility *T*-Test	9.11 ± 0.46
	All 89–90	NCAA D1	Collegiate	Agility *T*-Test	8.94 ± 0.34
	All 90–91	NCAA D1	Collegiate	Agility *T*-Test	9.00 ± 0.45
	All 91–92	NCAA D1	Collegiate	Agility *T*-Test	9.15 ± 0.41
Köklü et al. [79]	All	Turkish D1	Professional	Agility *T*-Test	9.49 ± 0.61
	All	Turkish D2	Professional	Agility *T*-Test	9.76 ± 0.57
	All	Turkish D1 and D2	Professional	Agility *T*-Test	9.61 ± 0.57
	Guard	Turkish D1 and D2	Professional	Agility *T*-Test	9.24 ± 0.56
	Forward	Turkish D1 and D2	Professional	Agility *T*-Test	9.48 ± 0.46
	Centre	Turkish D1 and D2	Professional	Agility *T*-Test	10.04 ± 0.35
Lehnert et al. [108]	All	Czech First League	Professional	Agility *T*-Test	9.35 ± 0.49
Maggioni et al. [58]	All	Volunteer Players	Semi-professional	Agility *T*-Test	9.8 ± 0.2
Pojskić et al. [89]	Perimeter	Bosnia and Herzegovina D1	Professional	Agility *T*-Test	10.48 ± 0.41
Poole et al. [112]	All	Australian State Level	Semi-professional	Agility *T*-Test	10.90 ± 0.51
Sekulic et al. [49]	Guard	Bosnia and Herzegovina D1 and D2	Professional	Agility *T*-Test	8.96 ± 0.37
	Forward	Bosnia and Herzegovina D1 and D2	Professional	Agility *T*-Test	8.84 ± 0.34
	Centre	Bosnia and Herzegovina D1 and D2	Professional	Agility *T*-Test	9.73 ± 0.50
	All	Bosnia and Herzegovina D1	Professional	Agility *T*-Test	9.02 ± 0.49
	All	Bosnia and Herzegovina D2	Professional	Agility *T*-Test	9.14 ± 0.43
Dawes and Spiteri [51]	All	NCAA D2	Collegiate	Lane Agility Test	11.24 ± 0.54
Lockie et al. [3]	All	NCAA D1	Collegiate	Lane Agility Test	10.42 ± 0.61
	Frontcourt	NCAA D1	Collegiate	Lane Agility Test	10.95 ± 0.78
	Backcourt	NCAA D1	Collegiate	Lane Agility Test	10.16 ± 0.33
Townsend et al. [111]	All	Collegiate D1 United States	Collegiate	Lane Agility Test	11.80 ± 0.90
Lockie et al. [113]	All	Semi-Professional	Semi-professional	COD Left foot start	1.88 ± 0.09
	All	Semi-Professional	Semi-professional	COD Right foot start	1.88 ± 0.14
	All	Recreational	Amateur	COD Left foot start	1.94 ± 0.12
	All	Recreational	Amateur	COD Right foot start	1.96 ± 0.14
Pehar et al. [85]	All	Bosnia and Herzegovina D1	Professional	Y-COD	1.68 ± 0.15
Scanlan et al. [60]	All	Australian State Level	Semi-professional	Y-CODST	1.64 ± 0.10
	All ^STARTERS^	Australian State Level	Semi-professional	Y-CODST	1.65 ± 0.11
	All ^NON−STARTERS^	Australian State Level	Semi-professional	Y-CODST	1.63 ± 0.10
Scanlan et al. [21]	All	Australian State Level	Semi-professional	Y-CODST	1.64 ± 0.10
Scanlan et al. [4]	Backcourt	Australian State Level	Semi-professional	Y-CODST	1.67 ± 0.10
	Frontcourt	Australian State Level	Semi-professional	Y-CODST	1.61 ± 0.11

Data presented as mean ± standard deviation

*88–89* data collected from collegiate team during the 1988–90 season, *89–90* data collected from collegiate team during the 1989–90 season, *90*–*91* ata collected from collegiate team during the 1990–91 season, *91–92* data collected from collegiate team during the 1991–2 season, *Backcourt players* guards, *BUSA* British Universities Sports Association, *COD* change-of-direction, *CODST* Change-of-Direction Speed Test, *D1* Division one competition, *D2* Division two competition, *D4* Division four competition, *Frontcourt players* forwards and centres, *Liga EBA* Liga Española de Baloncesto Aficionado, *NCAA* National Collegiate Athletic Association, *perimeter* point guard, shooting guard and small forward positions, *PSBC* Paulista State Basketball Championships, *U20* under 20 years of age competition

Modified *T*-Test requires players to sprint 5 m forward, shuffle 2.5 m laterally to the left, then shuffle 5 m to the right, shuffle 2.5 m to the left and then backpedal to the starting position

### Agility

Agility performance was reported in seven (5%) of the 137 studies included in this review (Table 7 of the ESM). Only three tests were used to assess agility including the Reactive Agility Test [4, 21, 60], Reactive Change-of-Direction Test [113, 118], and Reactive Y-Shaped Change-of-Direction Test [49, 85]. Time was the primary outcome variable reported across studies in all agility tests, with response time and decision-making time also reported in three studies [4, 21, 60]. Agility tests were performed slower compared to the pre-determined change-of-direction speed tests following the same design [4, 21, 49, 60, 113, 118]. The Reactive Agility Test was performed exclusively at the semi-professional level [4, 21, 60] and performance ranged between 2.00 and 2.18 s. Reactive COD Test performance ranged between 2.52 and 2.77 s at the semi-professional [113, 118] level. Only one study [113] reported Reactive COD performance at the amateur level (Table 9). The Reactive Y-COD test was only reported in two studies [48, 49] (Table 9). No studies measured the agility of players at collegiate or representative levels. Only one study [49] reported agility performance by playing position (Table 9). Reliability statistics for each of the agility tests described in Table 9 are provided in Table 13 of the ESM.

**Table 9:** Agility performance according to playing position and competition level in adult male basketball players

Study	Playing position	Competition level	Category	Test	Time (s)	Response time (ms)	Decision-making time (ms)
Jeffries et al. [118]	All	Australian State Level	Semi-professional	Reactive COD left leg start	2.77 ± 0.17		
	All	Australian State Level	Semi-professional	Reactive COD right leg start	2.75 ± 0.19		
Lockie et al. [113]	All	Australian State Level	Semi-professional	Reactive COD left leg start	2.52 ± 0.17		
	All	Australian State Level	Semi-professional	Reactive COD right leg start	2.53 ± 0.19		
	All	Australia Recreational Competition	Amateur	Reactive COD left leg start	2.67 ± 0.13		
	All	Australia Recreational Competition	Amateur	Reactive COD right leg start	2.70 ± 0.12		
Pehar et al. [85]	All	Bosnia and Herzegovina D1	Professional	Reactive Y-COD	1.99 ± 0.15		
Scanlan et al. [4]	Backcourt	Australian State Level	Semi-professional	Reactive Agility Test	2.1 ± 0.13	367 ± 140	128 ± 30
	Frontcourt	Australian State Level	Semi-professional	Reactive Agility Test	2.09 ± 0.17	368 ± 137	129 ± 42
Scanlan et al. [21]	All	Australian State Level	Semi-professional	Reactive Agility Test	2.09 ± 0.04	367 ± 132	128 ± 35
Scanlan et al. [60]	All	Australian State Level	Semi-professional	Reactive Agility Test	2.09 ± 0.14	367 ± 132	128 ± 35
	All ^STARTERS^	Australian State Level	Semi-professional	Reactive Agility Test	2.00 ± 0.12	308 ± 101	111 ± 27
	All ^NON−STARTERS^	Australian State Level	Semi-professional	Reactive Agility Test	2.18 ± 0.09	427 ± 141	146 ± 35
Sekulic et al. [49]	Guard	Bosnia and Herzegovina D1 and D2	Professional	Reactive Y-COD dominant leg start	1.94 ± 0.14		
	Forward	Bosnia and Herzegovina D1 and D2	Professional	Reactive Y-COD dominant leg start	1.95 ± 0.17		
	Centre	Bosnia and Herzegovina D1 and D2	Professional	Reactive Y-COD dominant leg start	2.04 ± 0.17		
	All	Bosnia and Herzegovina D1	Professional	Reactive Y-COD dominant leg start	1.93 ± 0.13		
	All	Bosnia and Herzegovina D2	Professional	Reactive Y-COD dominant leg start	2.03 ± 0.19		
	Guard	Bosnia and Herzegovina D1 and D2	Professional	Reactive Y-COD non-dominant leg start	2.08 ± 0.15		
	Forward	Bosnia and Herzegovina D1 and D2	Professional	Reactive Y-COD non-dominant leg start	2.10 ± 0.13		
	Centre	Bosnia and Herzegovina D1 and D2	Professional	Reactive Y-COD non-dominant leg start	2.15 ± 0.18		
	All	Bosnia and Herzegovina D1	Professional	Reactive Y-COD non-dominant leg start	2.06 ± 0.14		
	All	Bosnia and Herzegovina D2	Professional	Reactive Y-COD non-dominant leg start	2.16 ± 0.17		

Data presented as mean ± standard deviation

*Backcourt players* guards, *COD* change-of-direction, *D1* Division one competition, *D2* Division two competition, *Frontcourt players* forwards and centres, *Y-COD* Y-shaped Change-of-Direction, *Y-CODST* Y-Shaped Change-of-Direction Speed Test

### Strength

Strength testing was undertaken in 42 (31%) of the 137 studies in this review (Table 8 of the ESM). Repetition maximum outcome variables were most frequently gathered across studies, with 1RM and 3RM being the most used protocols (Table 8 of the ESM). Bench press performance, represented by 1RM were observed in 17 studies (40% of studies assessing strength) [51, 56, 65, 67, 70–72, 98, 99, 102–105, 114, 119–121], with mean loads lifted between 70 and 112 kg (Table 10). Professional players bench pressed 1RM loads between 70 and 112 kg (Table 10). Only two studies reported bench press 1RM each in semi-professional (76–86 kg) [70, 71] and representative (77–101 kg) [65, 114] players. Collegiate players bench pressed 1RM loads between 76 and 102 kg (Table 10). Only one study [98] reported bench press 1RM in amateur players and only one study [121] reported bench press 1RM by playing position, at the professional level (Table 10).

**Table 10:** Bench press and back squat 1RM results according to playing position and competition level in adult male basketball players

Study	Playing position	Competition level	Category	Bench press (kg)	Squat (kg)
Balabinis et al. [98]	All	Greek undergraduate-aged teams	Amateur	85.2 ± 0.4	
Balsalobre-Fernandez et al. [99]	All	Spanish Pro Liga ACB	Professional	101.9 ± 12.5^S/M^	
Ben Abdelkrim et al. [65]	All (U20)	Tunisian National Team	Representative	76.7 ± 8.9	183.3 ± 17.8
	All	Tunisian National Team	Professional	87.7 ± 14.3	201.5 ± 16.2
Cabarkapa et al. [124]	All	NCAA D1^(NBA)^	Collegiate		153.3 ± 26.2
	All	NCAA D1^(Pro)^	Collegiate		144.6 ± 23.8
Chaouachi et al. [67]	All	Tunisian National Team	Professional	79.0 ± 6.0	143.0 ± 13.4^a^
Dawes and Spiteri [51]	All	NCAA D2	Collegiate	96.2 ± 17.0^	134.4 ± 19.3^
de Sousa Fortes et al. [59]	All	State Basketball Championship Brazil	Professional		187.9 ± 22.9^S/M^
Delextrat and Cohen [114]	All	BUSA D1 and D2	Representative-Collegiate	91.9 ± 25.6	
	First Team	BUSA D1	Representative	101.3 ± 26.9	
	Second Team	BUSA D2	Collegiate	82.5 ± 24.0	
Freitas et al. [70]	All	Spanish Liga EBA D4	Semi-professional	85.8 ± 20.3^S/M,v^	157.4 ± 22^S/M,a^
Freitas et al. [71]	All	Spanish Liga EBA D4	Semi-professional	76.4 ± 14.2^S/M,v^	149.1 ± 23.0^S/M,a^
Gillam [119]	All	NCAA D2	Collegiate	76.3 ± 11.3	115.9 ± 18.0
Gomes et al. [72]	All	PSBC	Professional	105.9 ± 18.3	
Hoffman et al. [103]	All	NCAA D1	Collegiate	84.1 ± 12.2	119.4 ± 25.2
Hoffman et al. [102]	All	NCAA D1	Collegiate	87.4 ± 14.3	140.7 ± 21.0
Hoffman et al. [104]	All 88–89	NCAA D1	Collegiate	88.1 ± 14.5	143.4 ± 24.3
	All 89–90	NCAA D1	Collegiate	97.0 ± 19.2	145.9 ± 24.4
	All 90–91	NCAA D1	Collegiate	101.6 ± 20.2	155.9 ± 18.6
	All 91–92	NCAA D1	Collegiate	102.1 ± 19.1	
Hunter et al. [120]	All	Collegiate U.S	Collegiate	79.5 ± 11.8	
Hunter et al. [105]	All	NCAA D1A	Collegiate	91.4 ± 18.5	139.3 ± 7.4
Kariyawasam et al. [56]	All	National Level Sri Lanka	Professional	111.6 ± 64.1	
Parr et al. [121]	Guard	NBA	Professional	86.6 ± 14.9^ h^	
	Forward	NBA	Professional	101.1 ± 20.8^ h^	
	Centre	NBA	Professional	69.9 ± 0.0^ h^	

Data are presented as mean ± standard deviation

*1RM* one repetition maximum, *88–89* data collected from collegiate team during the 1988–90 season, *89–90* data collected from collegiate team during the 1989–90 season, *90–91* data collected from collegiate team during the 1990–91 season, *91–92* data collected from collegiate team during the 1991–92 season, *BUSA* British Universities Sports Association, *D1* Division one competition, *D2* Division two competition, *D4* Division four competition, *Liga ACB* Liga Endesa Asociación de Clubs de Baloncesto, *Liga EBA* Liga Española de Baloncesto Aficionado, *NBA* National Basketball Association, *NCAA* National Collegiate Athletic Association, *PSBC* Paulista State Basketball Championships, *U20* under 20 years of age competition, ^a^ indicates half squat, ^h^ indicates measured using hydraulic isokinetic bench press, ^(NBA)^ indicates collegiate player who went on to play in the NBA after college, ^(Pro)^ indicates collegiate player who went on to play professionally outside of the NBA after college, ^S/M^ indicates Smith Machine, ^V^ indicates 1RM estimated using velocity measures, ^ indicates 1RM calculated from 3RM testing

The squat exercise (i.e., front and back squat) was used in 16 studies (38% of studies assessing strength) [51, 59, 65, 67, 70, 71, 91, 98, 102–104, 111, 119, 122–124] to assess strength with 1RM and 3RM protocols most frequently used (Table 8 of the ESM). Mean back squat 1RM loads ranged between 116 and 202 kg across studies (Table 10). Professional players squatted greater mean 1RM loads (143–202 kg) than collegiate players (116–156 kg) (Table 10). Only two studies [70, 71] reported back squat 1RM loads in semi-professional players (149–157 kg), while one study [65] assessed back squat 1RM load in representative players. No studies reported back squat performance in amateur players or relative to playing position. All additional strength tests and outcome variables are reported in Table 8 of the ESM, while the bench press and squat outcome variables reported in individual studies are shown in Table 10. Reliability statistics for each of the strength tests described in Table 10 are provided in Table 13 of the ESM.

### Anaerobic Capacity

Anaerobic capacity was assessed in 35 studies (26%) of the 137 studies in this review, using 20 different tests (Table 9 of the ESM). The most frequently implemented tests were the WAnT (nine studies, 26% of studies assessing anaerobic capacity) [61, 98, 109, 114, 117, 125–128], the RAST (six studies, 18% of studies assessing anaerobic capacity) [87–89, 99, 100, 129], and the full court shuttle run (five studies, 14% of studies assessing anaerobic capacity) [28, 51, 58, 116, 125]. Peak power, mean power, fatigue index, and time were the most reported outcome variables (Table 11). Performance during the WAnT was reported in seven studies [61, 109, 117, 125–128] at the professional level (mean power: 683–823 W, peak power: 951–1085 W, and fatigue index: 43–60%). However, only one study reported WAnT performance each in representative [114] and collegiate [114] players, while two studies [98, 125] were observed at the amateur level (Table 11). No studies reported WAnT performance in semi-professional players. Two studies [109, 126] reported WAnT performance according to playing position (guards: peak power: 11–13 W/kg, fatigue index: 48–64%, forwards: peak power: 11–13 W/kg, fatigue index: 43–58%, centres: 10–11 W/kg, fatigue index: 44–56%). The RAST was only reported in professional players [87–89, 99, 100, 129], with mean peak power ranging between 761 and 957 W and mean power between 608 and 772 W. Two studies [88, 129] reported RAST performance according to playing position. Four studies used the full court shuttle run, with professional [125], semi-professional [28, 58], collegiate [51] and amateur [125] players assessed (Table 11). The full court shuttle run was only reported relative to playing position in one study [28] consisting of semi-professional players (Table 11). Reliability statistics for each of the anaerobic capacity tests described in Table 11 are provided in Table 13 of the ESM.

**Table 11:** Repeated sprint, running-based anaerobic sprint test and Wingate anaerobic cycle test performance according to playing position and competition level in adult male basketball players

Study	Playing position	Competition level	Category	Test	Variable	Outcome
Dawes and Spiteri [51]	All	NCAA D2	Collegiate	Full court shuttle run	Time (s)	27.8 ± 0.9
Fatouros et al. [125]	All	Greek D2 Competition	Professional	Full court shuttle run	Time (s)	27.4 ± 0.7
	All	Greek Recreational	Amateur	Full court shuttle run	Time (s)	29.2 ± 0.9
Maggioni et al. [58]	All	Volunteer Players	Semi-professional	Full court shuttle run	Time (s)	27.8 ± 0.8
Montgomery et al. [28]	All	Australian State Level	Semi-professional	Full court shuttle run	Time (s)	27.5 ± 1.2
	Guard	Australian State Level	Semi-professional	Full court shuttle run	Time (s)	26.9 ± 0.9
	Forward	Australian State Level	Semi-professional	Full court shuttle run	Time (s)	27.9 ± 1.4
	Centre	Australian State Level	Semi-professional	Full court shuttle run	Time (s)	27.6 ± 1.4
Pojskić et al. [87]	All	Bosnian Premier League	Professional	RAST	Maximal power (W)	761 ± 125
					Mean power (W)	620 ± 99.4
					Fatigue index (W/s)	8.2 ± 1.4
Pojskić et al. [88]	Guard	Bosnian Premier League	Professional	RAST	Maximal power (W)	773 ± 129
					Mean power (W)	635 ± 110
					Fatigue index (W/s)	8.1 ± 2.5
	Forward	Bosnian Premier League	Professional	RAST	Maximal power (W)	762 ± 123
					Mean power (W)	608 ± 89.6
					Fatigue index (W/s)	8.8 ± 2.7
	Centre	Bosnian Premier League	Professional	RAST	Maximal power (W)	858 ± 109
					Mean power (W)	713 ± 69.5
					Fatigue index (W/s)	10.5 ± 2.24
Pojskić et al. [89]	All	Bosnian and Herzegovina D1	Professional	RAST	Maximal power (W)	761 ± 125
					Mean power (W)	620 ± 99.4
					Fatigue index (W/s)	8.2 ± 1.4
Balsalobre-Fernandez et al. [99]	All	Spanish Pro Liga ACB	Professional	RAST	Peak power (W)	957 ± 194
					Mean power (W)	772 ± 126
					Fatigue index (%)	33.1 ± 8.0
Balsalobre-Fernandez et al. [100]	All	Spanish Pro Liga ACB	Professional	RAST	Peak power (W)	889 ± 230^WPD^
					Mean power (W)	737 ± 134^WPD^
					Fatigue index (%)	32.4 ± 7.2^WPD^
de Araujo et al. [129]	All	Elite National Team and League Brazil	Professional	RAST	Peak power (W)	901 ± 39.1
					Mean power (W)	701 ± 22.7
					Fatigue index (%)	41.5 ± 2.5
	Guard	Elite National Team and League Brazil	Professional	RAST	Peak power (W)	853 ± 59.0
					Mean power (W)	683 ± 49.0
					Fatigue index (%)	42.1 ± 4.5
	Forward	Elite National Team and League Brazil	Professional	RAST	Peak power (W)	923 ± 79.8
					Mean power (W)	700 ± 40.7
					Fatigue index (%)	40.6 ± 4.4
	Centre	Elite National Team and League Brazil	Professional	RAST	Peak power (W)	912 ± 42.0
					Mean power (W)	717 ± 26.4
					Fatigue index (%)	42.1 ± 4.3
Alemdaroglu [61]	All	Turkish D1	Professional	Wingate Anaerobic Cycle Test	Peak power (W) Mean power (W) Fatigue index (%)	955 ± 118 703 ± 79.3 54.7 ± 7.3
Balabinis et al. [98]	All	Undergraduate Teams Greece	Amateur	Wingate Anaerobic Cycle Test	Peak power (W)	841 ± 92.4
Delextrat and Cohen [114]	All	BUSA D1	Representative	Wingate Anaerobic Cycle Test	Peak power (W/kg)	10.2 ± 0.9*
					Mean power (W/kg)	8.2 ± 0.9*
					Fatigue index (%)	57.4 ± 14.9
	All	BUSA D3	Collegiate	Wingate Anaerobic Cycle Test	Peak power (W/kg)	10 ± 0.9*
					Mean power (W/kg)	7.8 ± 1.1*
					Fatigue index (%)	47.2 ± 15.1
Fatouras et al. [125]	All	Greek D2 Competition	Professional	Wingate Anaerobic Cycle Test	Peak power (W/kg)	11.2 ± 2.1
					Mean power (W/kg)	9.3 ± 0.9
	All	Greek Recreational	Amateur	Wingate Anaerobic Cycle Test	Peak power (W/kg)	9.7 ± 1.9
					Mean power (W/kg)	7.2 ± 2.1
Harbili [128]	All	Turkish National League D3	Professional	Wingate Anaerobic Cycle Test	Peak power (W)	951.6 ± 86.9
					Peak power (W/kg)	10.3 ± 1.4
					Mean power (W)	683.7 ± 40.5
					Mean power (W/kg)	7.4 ± 0.8
					Fatigue index (%)	59.9 ± 6.3
Nikolaidis et al. [109]	Guard	Italian First League	Professional	Wingate Anaerobic Cycle Test	Peak power (W)	992 ± 155
					Peak power (W/kg)	11.4 ± 1.1
					Mean power (W)	737 ± 78
					Mean power (W/kg)	8.5 ± 0.7
					Fatigue index (%)	47.5 ± 6.6
	Forward	Italian First League	Professional	Wingate Anaerobic Cycle Test	Peak power (W)	1052 ± 93
					Peak power (W/kg)	11.1 ± 1.1
					Mean power (W)	823 ± 94
					Mean power (W/kg)	8.7 ± 1.1
					Fatigue index (%)	42.9 ± 4.7
	Centre	Italian First League	Professional	Wingate Anaerobic Cycle Test	Peak power (W)	1085 ± 93
					Peak power (W/kg)	10 ± 1.1
					Mean power (W)	807 ± 91
					Mean power (W/kg)	7.4 ± 0.8
					Fatigue index (%)	44.4 ± 5.5
Popadic Gacesa et al. [127]	All	Elite Serbian Players	Professional	Wingate Anaerobic Cycle Test	Peak power (W) Peak power (W/kg) Mean power (W) Mean power (W/kg)	1001 ± 150 10.7 ± 1.7 669 ± 77.1 7.2 ± 1
Sallet et al. [126]	All	French D1 and D2	Professional	Wingate Anaerobic Cycle Test	Peak power (W/kg)	12.2 ± 2.7
					Fatigue index (%)	58.9 ± 13.5
	Guards	French D1 and D2	Professional	Wingate Anaerobic Cycle Test	Peak power (W/kg) Fatigue index (%)	13.1 ± 1.7
						63.8 ± 14.7
	Forwards	French D1 and D2	Professional	Wingate Anaerobic Cycle Test	Peak power (W/kg)	12.7 ± 3.5
					Fatigue index (%)	58.1 ± 9.3
	Centres	French D1 and D2	Professional	Wingate Anaerobic Cycle Test	Peak power (W/kg)	11.1 ± 2.1
					Fatigue index (%)	56.3 ± 15.7
Soslu et al. [117]	All	Turkish Professional Competition	Professional	Wingate Anaerobic Cycle Test	Peak power (W) Peak power (W/kg) Mean power (W) Mean power (W/kg)	849 ± 128 12.4 ± 3.0 681 ± 86 8.7 ± 1.2

Data are presented as mean ± standard deviation

*BUSA* British Universities Sports Association, *D1* Division one competition, *D2* Division two competition, *D3* Division three competition, *Liga ACB* Liga Endesa Asociación de Clubs de Baloncesto, *NCAA* National Collegiate Athletic Association, *RAST* Running-based Anaerobic Sprint Test, ^WPD^ indicates data retrieved using WebPlotDigitizer

### Aerobic Capacity

Aerobic capacity was assessed in 57 (42%) of the 137 studies included in this review, with 14 different tests used (Table 10 of the ESM). Incremental treadmill tests (17 studies, 30% of studies assessing aerobic capacity), which involved the Bruce [105, 120, 130] and various incremental running protocols [27, 57–59, 90, 94, 121, 125, 126, 131–135], as well as the Yo-Yo IRL1 (14 studies, 25% of studies assessing aerobic capacity) [4, 16, 21, 28, 55, 58, 60, 63, 65, 67, 72, 136–138] and MSFT (eight studies, 14% of studies assessing aerobic capacity) [51, 61, 79, 84, 87–89, 107] were the most frequently used tests. The most common outcome variable reported from aerobic testing was maximum oxygen uptake (VO_2max_) [Table 10 of the ESM]. However, if during a maximal test the criteria for VO_2max_ was not achieved, VO_2peak_ was reported as the outcome variable [27, 131, 134].

Mean aerobic capacity during incremental treadmill tests ranged from 42 to 61 mL/kg/min across studies (Table 12). The mean aerobic capacity of professional players ranged between 42 and 61 mL/kg/min. Only one study [135] assessed aerobic capacity in semi-professional players using an incremental treadmill test, while no studies assessed representative players. Mean aerobic capacity in collegiate players ranged between 50 and 58 mL/kg/min. Aerobic capacity in amateur players were only assessed in two studies [125, 135] using an incremental treadmill test. When observing aerobic capacity according to playing position using incremental treadmill tests, only professional player data were apparent [27, 90, 121, 126, 131] with mean aerobic capacity in guards ranging between 50 and 58 mL/kg/min, in forwards between 46 and 58 mL/kg/min and in centres between 42 and 58 mL/kg/min.

**Table 12:** Maximum oxygen uptake and distance variables during aerobic capacity tests according to playing position and competition level in adult male basketball players

Study	Playing position	Competition level	Category	Test	VO_2_max (ml/kg/min)	Distance (m)
Bolonchuk et al. [130]	All	U.S. Collegiate	Collegiate	Incremental Treadmill Test	53.8 ± 4.5	
Boone and Bourgois [27]	Point guard	Belgian D1	Professional	Incremental Treadmill Test	57.4 ± 4.8^peak^	
	Shooting guard	Belgian D1	Professional	Incremental Treadmill Test	55.3 ± 3.6^peak^	
	Small forward	Belgian D1	Professional	Incremental Treadmill Test	52.9 ± 5.6^peak^	
	Power forward	Belgian D1	Professional	Incremental Treadmill Test	50.4 ± 5.2^peak^	
	Centre	Belgian D1	Professional	Incremental Treadmill Test	50.9 ± 5.2^peak^	
Boone et al. [131]	Guard	Belgian D1	Professional	Incremental Treadmill Test	60.3 ± 3.7^peak^	
	Forward	Belgian D1	Professional	Incremental Treadmill Test	56.4 ± 4.1^peak^	
	Centre	Belgian D1	Professional	Incremental Treadmill Test	52.7 ± 3.4^peak^	
Chatzinikolaou et al. [132]	All	National Division Greece	Professional	Incremental Treadmill Test	54.5 ± 2.9	
de Sousa Fortes et al. [59]	All	State Basketball Championship Brazil	Professional	Incremental Treadmill Test	52.6 ± 6.1	
Dragonea et al. [133]	All	Greek A1 and A2 Leagues	Professional	Incremental Treadmill Test	51.6 ± 3.8	
Fatouros et al. [125]	All	Greek D2	Professional	Incremental Treadmill Test	53.4 ± 8.5	
	All	Greek Recreational Competition	Amateur	Incremental Treadmill Test	47.6 ± 6.9	
Hunter et al. [120]	All	U.S College	Collegiate	Incremental Treadmill Test	49.7 ± 8.5	
Hunter et al. [105]	All	NCAA D1A	Collegiate	Incremental Treadmill Test	50.0 ± 7.7	
Maggioni et al. [58]	All	Volunteer players	Semi-professional	Incremental Treadmill Test	54.2 ± 4.1	
McInnes et al. [134]	All	NBL	Professional	Incremental Treadmill Test	60.7 ± 8.6^peak^	
Metaxas et al. [135]	All	Greek National League D1	Professional	Incremental Treadmill Test	51.3 ± 4.1	
	All	Greek National League D2	Professional	Incremental Treadmill Test	50.4 ± 5.4	
	All	Greek National League D3	Semi-professional	Incremental Treadmill Test	47.8 ± 5.3	
	All	Greek National League D4	Amateur	Incremental Treadmill Test	49.1 ± 5.6	
Narazaki et al. [57]	All	NCAA D1	Collegiate	Incremental Treadmill Test	57.5 ± 8.2	
Parr et al. [121]	Guard	NBA	Professional	Incremental Treadmill Test	50.0 ± 5.4	
	Forward	NBA	Professional	Incremental Treadmill Test	45.9 ± 4.3	
	Centre	NBA	Professional	Incremental Treadmill Test	41.9 ± 4.9	
Ponce-González et al. [90]	All	Spanish Pro Liga ACB	Professional	Incremental Treadmill Test	57.7 ± 5.5	
	Guard	Spanish Pro Liga ACB	Professional	Incremental Treadmill Test	58.0 ± 5.0	
	Forward	Spanish Pro Liga ACB	Professional	Incremental Treadmill Test	57.5 ± 4.6	
	Centre	Spanish Pro Liga ACB	Professional	Incremental Treadmill Test	57.5 ± 8.7	
	Perimeter	Spanish Pro Liga ACB	Professional	Incremental Treadmill Test	57.0 ± 4.3	
	Inside	Spanish Pro Liga ACB	Professional	Incremental Treadmill Test	58.7 ± 7.5	
Sallet et al. [126]	All	French League D1 and D2	Professional	Incremental Treadmill Test	54.9 ± 7.2	
	Guard	French League D1 and D2	Professional	Incremental Treadmill Test	57.5 ± 9.2	
	Forward	French League D1 and D2	Professional	Incremental Treadmill Test	55.2 ± 6.5	
	Centre	French League D1 and D2	Professional	Incremental Treadmill Test	52.9 ± 6.2	
	All	French League D1	Professional	Incremental Treadmill Test	53.7 ± 6.7	
	All	French League D2	Professional	Incremental Treadmill Test	56.5 ± 7.7	
Stojanovic et al. [94]	All	Serbian Professional League	Professional	Incremental Treadmill Test	51.9 ± 4.1	
Aoki et al. [63]	All	Brazilian National League	Professional	Yo-Yo IRL1		1120 ± 413
Ben Abdelkrim et al. [65]	All (U20)	Tunisian National Team	Representative	Yo-Yo IRL1	55.4 ± 4.6	
	All	Tunisian National Team	Professional	Yo-Yo IRL1	59.9 ± 5.3	
Chaouachi et al. [67]	All	Tunisian National Team	Professional	Yo-Yo IRL1	59.1 ± 6.2	2389 ± 616
Ferioli et al. [136]	All All	Italian Serie A and Serie A2 Italian Serie B	Professional Semi-professional	Yo-Yo IRL1 Yo-Yo IRL1		1669 ± 357 1708 ± 444
Ferioli et al. [16]	All	Italian Serie A2	Professional	Yo-Yo IRL1		2135 ± 356
	All	Italian Serie B	Semi-professional	Yo-Yo IRL1		2265 ± 578
	All	Italian Serie D	Amateur	Yo-Yo IRL1		1671 ± 370
	Guard	Italian Serie A-D	Amateur – Professional	Yo-Yo IRL1		2447 ± 427
	Forward	Italian Serie A-D	Amateur – Professional	Yo-Yo IRL1		2078 ± 350
	Centre	Italian Serie A-D	Amateur – Professional	Yo-Yo IRL1		1853 ± 524
Ferioli et al. [55]	All	Italian Serie A2	Professional	Yo-Yo IRL1		1765 ± 324
	All	Italian Serie B	Semi-professional	Yo-Yo IRL1		1610 ± 330
Gomes et al. [72]	All	PSBC	Professional	Yo-Yo IRL1	46.7 ± 2.8	
Maggioni et al. [58]	All	Volunteer Players	Semi-professional	Yo-Yo IRL1		1445 ± 420
Montgomery et al. [28]	All	Australian State Level	Semi-professional	Yo-Yo IRL1		1592 ± 629
	Guard	Australian State Level	Semi-professional	Yo-Yo IRL1		1807 ± 701
	Forward	Australian State Level	Semi-professional	Yo-Yo IRL1		1372 ± 537
	Centre	Australian State Level	Semi-professional	Yo-Yo IRL1		1500 ± 528
Scanlan et al. [137]	All	Australian State Level	Semi-professional	Yo-Yo IRL1		1283 ± 362
	All	Australian Recreational Competition	Amateur	Yo-Yo IRL1		636 ± 297
Scanlan et al. [4]	Backcourt	Australian State Level	Semi-professional	Yo-Yo IRL1	51.9 ± 4.8	
	Frontcourt	Australian State Level	Semi-professional	Yo-Yo IRL1	47.1 ± 5.0	
Scanlan et al. [21]	All	Australian State Level	Semi-professional	Yo-Yo IRL1	49.5 ± 5.3	
Scanlan et al. [60]	Starters	Australian State Level	Semi-professional	Yo-Yo IRL1	48.4 ± 6.6	
	Non-starters	Australian State Level	Semi-professional	Yo-Yo IRL1	50.6 ± 3.9	
Scanlan et al. [138]	All	Australian Regional and State level	Semi-professional	Yo-Yo IRL1		996 ± 464
Alemdaroğlu [61]	All	Turkish D1	Professional	Multi-Stage Fitness Test	50.6 ± 6.7	
Dawes and Spiteri [51]	All	NCAA D2	Collegiate	Multi-Stage Fitness Test	41.8 ± 3.5	66 ± 10^SHUT^
Köklü et al. [79]	All	Turkish D1	Professional	Multi-Stage Fitness Test	42.5 ± 8.6	
	All	Turkish D2	Professional	Multi-Stage Fitness Test	44.5 ± 7.0	
	Guard	Turkish D1 and D2	Professional	Multi-Stage Fitness Test	45.4 ± 8.3	
	Forward	Turkish D1 and D2	Professional	Multi-Stage Fitness Test	43.3 ± 7.2	
	Centre	Turkish D1 and D2	Professional	Multi-Stage Fitness Test	42.1 ± 8.1	
Korkmaz and Karahan [107]	All	Turkish D1	Professional	Multi-Stage Fitness Test	55.6 ± 2.6	
	All	Turkish D2	Professional	Multi-Stage Fitness Test	57.2 ± 2.8	
	All	Turkish D3	Semi-professional	Multi-Stage Fitness Test	50.5 ± 4.9	
Ostojic et al. [84]	All	First National League Serbia	Professional	Multi-Stage Fitness Test	49.8 ± 4.9	
	Guard	First National League Serbia	Professional	Multi-Stage Fitness Test	52.5 ± 4.8	
	Forward	First National League Serbia	Professional	Multi-Stage Fitness Test	50.7 ± 2.3	
	Centre	First National League Serbia	Professional	Multi-Stage Fitness Test	46.3 ± 4.9	
Pojskić et al. [88]	Guard	Bosnian Premier League	Professional	Multi-Stage Fitness Test	64.4 ± 7.1	106 ± 14^SHUT^
	Forward	Bosnian Premier League	Professional	Multi-Stage Fitness Test	62.4 ± 6.1	102 ± 12^SHUT^
	Centre	Bosnian Premier League	Professional	Multi-Stage Fitness Test	57.9 ± 7.2	93 ± 15^SHUT^
Pojskić et al. [89]	Perimeter	Bosnia and Herzegovina D1	Professional	Multi-Stage Fitness Test	63.7 ± 6.8	
Pojskić et al. [87]	Perimeter	Bosnian Premier League	Professional	Multi-Stage Fitness Test	63.7 ± 6.8	

Data are presented as mean ± standard deviation

*Backcourt players* guards, *D1* Division one competition, *D2* Division two competition, *D3* Division three competition, *D4* Division four competition, *Frontcourt players* forwards and centres, *Inside* power forward and centre positions, *Liga ACB* Liga Endesa Asociación de Clubs de Baloncesto, *NBA* National Basketball Association, *NBL* Australian National Basketball League, *NCAA* National Collegiate Athletic Association, *Perimeter* point guard, shooting guard and small forward positions, *PSBC* Paulista State Basketball Championships, *U20* players competing in an under 20 years of age competition, *Yo-Yo IRL1* Yo-Yo Intermittent Recovery Test Level 1, ^peak^ indicates *V*O_2peak_, ^SHUT^ indicates number of shuttles completed

Estimated VO_2max_ from the MSFT ranged from 42 to 64 ml/kg/min, while the number of shuttles completed were between 66 and 106 across studies (Table 12). The VO_2max_ of professional players ranged from 42 to 64 mL/kg/min using the MSFT. Only one study reported VO_2max_ during the MSFT each in semi-professional [107] and collegiate [61] players (Table 12). No studies reported MSFT in representative or amateur players. Mean estimated VO_2max_ relative to playing position using the MSFT was only reported in professional players [79, 84, 88] with guards (45–64 mL/kg/min), forwards (43–62 mL/kg/min) and centres (42–58 mL/kg/min) showing similar data.

Mean estimated VO_2max_ derived during the Yo-Yo IRL1 ranged between 47 and 60 mL/kg/min, while mean distances reached were between 636 and 2447 m across studies (Table 12). The VO_2max_ of professional players using the Yo-Yo IRL1 (47–60 mL/kg/min) was only reported in three studies [65, 67, 72], while the mean distance covered ranged between 1120 and 2389 m. The VO_2max_ of semi-professional players using the Yo-Yo IRL1 (48–52 mL/kg/min) was reported in three studies [4, 21, 60], while mean distances covered ranged from 996 to 2265 m. VO_2max_ was only observed in one study using the Yo-Yo IRL1 in representative players [65] (Table 12). No studies used the Yo-Yo IRL1 to estimate VO_2max_ in collegiate players, while two studies [16, 137] reported distance using the Yo-Yo IRL1 in amateur players (636–1671 m). Yo-Yo IRL1 performance relative to playing position was only reported in semi-professional players using estimated VO_2max_ [4] and distance [28], and in one study [16], which reported the mean across amateur, semi-professional, and professional players (Table 12). Reliability statistics for each of the aerobic capacity tests described in Table 12 are provided in Table 13 of the ESM.

## Discussion

The aims of this systematic review were to (1) identify tests and outcome variables used to assess physical characteristics in adult male basketball players across all competition levels, (2) report a summary of anthropometric, muscular power, linear speed, change-of-direction speed, agility, strength, anaerobic capacity, and aerobic capacity in adult male basketball players based on playing position and competition level, and (3) introduce a framework outlining recommended testing approaches to quantify physical characteristics in adult male basketball players. As expected, the number of tests and outcome variables reported reveal extensive variability in how the physical characteristics of adult male basketball players are tested. An indirect finding of this review was the wide range of approaches and variability in procedures and calculations of basic outputs (e.g., jump height measured through flight time vs take-off velocity). Additionally, the validity and reliability statistics of commonly used tests were often not reported. Thus, it is difficult to draw firm conclusions about the physical characteristics of basketball players. The wide-ranging physical performances observed were likely influenced by the choice of test and methodology employed by researchers. These issues have made it difficult to establish consensus based on the basketball literature. To improve the overall understanding of the physical characteristics required to excel at different competition levels in adult male basketball, researchers and practitioners should consider: (1) the validity, reliability and standardisation of tests being employed; (2) the appropriateness and specificity of the tests being implemented; and (3) the ability of testing information to discern players of different playing positions and competition levels. It is important to ensure outcome variables gathered are valid and reliable in order to detect meaningful changes over time. As each test may have an inherent level of variability or ‘noise’, discerning changes that are practically relevant is critical. Particularly when using data to quantify player progression, or to use performance data gathered during a test to guide rehabilitation, or when monitoring performance and fatigue. Furthermore, it is advised that researchers attempt to align with practitioners to continue to develop standardised testing batteries (e.g., NBA draft combine) that optimally support the profiling of adult male basketball players.

### Tests and Outcome Variables

Anthropometric values of height and body mass were reported in all 114 studies eligible to address the second aim of this review. Body composition was primarily measured using low-cost, easy to implement tests, such as sum of skinfold measurements. Furthermore, muscular power was most commonly measured indirectly with a combination of three bilateral jumps (i.e., CMJ, CJ and SJ) that provide insight into varying speed-strength jump qualities [78, 139]. Linear sprint performances were primarily reported over distances of 5, 10, and 20 m. Of those three distances, 5 m, which is reflective of an athlete’s ability to accelerate and is similar to movements frequently encountered during match-play, was the least reported. The Agility *T*-Test was the most commonly used test to assess change-of-direction speed, potentially because of the ease of implementation and inclusion of basketball-specific lateral movements. However, in recent years, collegiate-level players from the NCAA were observed using the Lane Agility Test to assess change-of-direction speed [3, 51]. This trend may be attributed to researchers and practitioners implementing tests that align with the testing protocols adopted by the NBA Draft Combine. Agility tests were the least reported category of test in the literature, and despite all studies assessing agility using a similar Y-Shape design the distances covered and stimuli used varied. Strength tests primarily incorporated bench press and back squat 1RM protocols. The frequency of strength testing observations was less than jump, linear sprint, change-of-direction speed, anaerobic capacity, and aerobic capacity tests, which may be due to varying levels of resistance training competency, biomechanical constraints introduced by the typically larger anthropometric values of basketball players, and the residual fatigue often accumulated by maximal strength testing. The anaerobic capacity of basketball players was tested predominantly with repeated sprint protocols varying in distance or a resisted cycling WAnT test. Aerobic capacity was most frequently assessed using running incremental treadmill tests or estimated using the Yo-Yo IRL1 and the MSFT. The incremental treadmill test was primarily reported at the professional level, which may suggest the resources required to undertake the incremental treadmill test may not be feasible to obtain and implement across all competition levels.

The wide variety of testing approaches reported across studies make it challenging to establish normative data or identify minimum thresholds for physical characteristics in adult male basketball players. Researchers and practitioners are encouraged to diligently consider the tests they select when assessing physical characteristics in players, as each test and testing methodology has an inherent level of accuracy and reproducibility [140]. For example, when considering the methodology of jumping tests, a range of technology with varying levels of accuracy have been used to assess jump height, including force platforms [16, 61, 71, 110], three-dimensional cameras [110], contact mats [62, 83, 96], Vertec [107], and chalk marks on a wall [103, 104]. The nuances associated with the various methodologies could influence results and need to be considered when comparing results between studies [140]. As consensus is reached with choice of test and methodology employed to measure key physical characteristics in adult male basketball players, it will become easier to monitor players and develop meaningful normative physical standards. In this regard, researchers and practitioners are encouraged to collaborate with FIBA and national governing bodies to develop standardised testing guidelines for broad application to basketball teams.

Basketball practitioners now have an abundance of published tests and methodologies to consider prior to assessing players. Additionally, there are outcome variables provided in multiple tests that represent different physical abilities. For example, jump height during a SJ provides insight into the ability to express concentric-only force, whereas jump height during a CMJ reflects the ability to use elastic energy that is generated during the countermovement [141]. Further, jump height from a running vertical jump is a measure of jumping performance specific to most common game situations [142]. Interpreting test results may be further complicated by multiple methods being available to calculate the same outcome variable. For instance, modified reactive strength index is commonly calculated as jump height divided by contraction time, yet can be calculated using jump height determined via flight time or impulse momentum [101]. This example highlights a major concern as variables such as flight time may be manipulated by a change in movement strategy by players (e.g., tucking legs on descent) subsequently altering results. Therefore, there are many aspects of current test selection that can be improved so all findings can contribute to establishing meaningful normative reference data.

Considering the wide variety of testing options adopted by basketball researchers and practitioners, relevance to the sport must be maintained and tests that do not directly transfer to basketball match-play should be carefully considered prior to implementation. When researchers and practitioners are selecting a test, they are encouraged to determine whether the bioenergetic and biomechanical components of a test are relevant and applicable to the needs of adult male basketball players. Furthermore, it is recommended that basketball researchers and practitioners consult with each other so that testing approaches can continue to be refined.

Basketball practitioners have several considerations and constraints at the organisational and team level that may influence selecting an appropriate testing battery. The accessibility of resources, technology, the expertise of staff on hand, availability of players, and influence of additional stakeholders can all influence the testing procedures adopted. Once tests are selected, an additional consideration when testing is the motivation of players. It is important to ensure players are executing testing with maximal intent when maximal efforts are required to avoid reporting submaximal performances. Providing the appropriate environment for testing is important as athletes are often tested for varying purposes (e.g., team selection, pre-season assessment) and at times, may lack incentive to give maximal effort. Basketball practitioners are encouraged to emphasise the importance of maximal effort during testing, as players who are fitter may appear as more resilient, effective and desirable for coaches. Despite these constraints, basketball practitioners are encouraged to select tests that are able to provide data that can allow for the monitoring of player progression, ranking or differentiation of players, and appropriate prescription of subsequent training. Additionally, basketball researchers and practitioners may wish to report test outcomes using ratios or indices (e.g., speed for height, agility to height ratio) that account for individual anthropometry as this may be beneficial when attempting to compare players who play across multiple positions. By scaling outcomes gathered from physical tests, performance may be able to be normalised and account for differences in player body size in sports where stature and mass are wide ranging [143].

### Physical Characteristics

#### Anthropometry

Taller players with longer wingspans may have the ability to rebound the ball at greater heights, take up more space when defending an opponent, and more effectively contest shots. For a given speed of movement, skill set, and fitness level, basketball inherently favours taller players as reflected by anthropometry measures being the most frequently measured physical characteristics across studies in this review. When competition levels were grouped by mean team height, higher level players such as those competing professionally were taller than amateur players. However, the range of mean height observed across studies was similar for professional, semi-professional, representative, and collegiate adult male players. When height was reported relative to playing position, a clear trend emerged across competition levels with guards identified as the shortest players, forwards being taller than guards, and centres being the tallest players. Height and wingspan measured at the NBA Draft Combine have been identified as predictors of future playing performance in the NBA [144]. However, in many basketball studies, wingspan was scarcely reported [3, 51, 52]. To better understand the interaction of player height, wingspan and performance, basketball researchers and practitioners should measure and report wingspan alongside height and body mass as part of a standardised anthropometrical assessment.

Body mass followed a similar trend to height, with broad ranges observed at most competition levels. Mean body mass in semi-professional players exhibited the smallest range across studies of any competition level, possibly owing to the large representation in this review of players from Australian state-level competitions [4, 21, 28, 30, 60, 112, 113, 118, 137, 138, 145]. Furthermore, the body mass of players at higher competition levels tended to be heavier than players competing at lower levels likely because of players at higher levels possessing greater lean body mass and height. Positional differences in body mass were noted, with guards being lightest, forwards being heavier than guards, and centres heaviest [27, 49, 129]. This positional trend in body mass was apparent in players at the professional level; however, because of the lack of evidence available, positional differences in body mass at the semi-professional, representative, collegiate, and amateur levels are not yet clear.

#### Body Composition

Body composition was shown to differ between competition levels in adult male basketball players. In this regard, semi-professional, representative, and collegiate players typically possessed a lower proportion of body fat than amateur players. Data retrieved from the NBA Draft Combine showed elite players drafted into the NBA typically exhibit very low levels of body fat. This finding may be because of higher level players having more availability for training and greater access to performance resources (e.g., dietitians and strength and conditioning coaches) or it may be the case that selectors for the NBA prefer lean players, as how lean a player is may have the potential to influence their match-play and ability to perform basketball skills. However, the comparison of body fat percentages using skinfold assessments across studies in this review must be made with caution as the anatomical landmarks used were not always consistent. For example, when the sum of three skinfolds was used to calculate body fat percentage, they were taken from the chest, abdomen, and thigh [53, 56, 57], the triceps, abdomen, and thigh [4, 21, 60], and the triceps, chest, and subscapular [59] in different studies. A further consideration regarding body composition is often different techniques and equations can be used, leading to differences in body fat estimates. These variations in methodology may influence the results presented. When mean body fat percentage was reported according to playing position, similar ranges were observed between guards (7–20%), forwards (8–17%), and centres (7–21%). However, the range of body fat of each position was noticeably influenced by one study [109], and when data from this study were excluded, values decreased across playing positions (guards: 7–14%, forwards: 8–15%, and centres: 7–16%). These ranges may be a more appropriate summation of the body fat percentages in adult male players identified in this review and suggest body composition may be similar across playing positions.

A further consideration for basketball researchers and practitioners when interpreting or comparing results is the time of testing. For example, players may be assessed at the commencement of pre-season and record considerably different results compared with the middle of season, when they are likely insuperior physical condition. When measuring anthropometry, it is recommended basketball practitioners initiate their testing batteries with measures of height, body mass, body composition, and wingspan as these measurements may change after the initiation of other tests (e.g., fluid loss from an aerobic capacity test). Throughout this review, the phase of the basketball season and where testing occurred was often not reported. To improve future basketball research, it is strongly encouraged that when the testing occurs is clearly stated when reporting the physical characteristics of basketball athletes. Furthermore, when using sum of skinfolds to measure body composition, the specific sites should be clearly identified and used repeatedly over time for consistent measurements. The use of an International Society for the Advancement of Kinanthropometry-certified anthropometrist may also be of benefit for the accuracy and reproducibility of measurements [146]. It is recommended to use the sum of eight skinfold sites, measured at the biceps, triceps, subscapular, iliac crest, supraspinale, abdominal, anterior thigh and medial calf, in line with the recommended protocols outlined by the International Society for the Advancement of Kinanthropometry due to the efficiency of testing, ability to detect meaningful change over time, and standardisation of measurement [147].

#### Muscular Power

Well-developed muscular power is favourable to meet the physical demands of basketball match-play [16, 104, 114, 148]. Jump variables were used most frequently to indirectly assess muscular power characteristics, which may be due to the range of tasks specific to basketball involving various forms of jumps (e.g., rebounding, contesting a shot) and the high frequency of jumping executed throughout matches [12, 149, 150]. The greatest jump heights reported in the literature were from the VJ test in collegiate players (77 ± 6 cm) [111] and players who were assessed at the NBA Draft Combine (77 ± 8 cm). Collegiate players (44–83 cm) tended to record higher VJ performances than professional players (36–63 cm), and semi-professional players (35–50 cm), while insufficient studies were observed at the representative and amateur levels to draw conclusions. No differences in VJ height were observed between playing positions at the professional level with insufficient studies available across other playing levels to make conclusions regarding positional differences. Considering there were discrepancies between professional, semi-professional, and collegiate players observed, it is important to consider that different testing methods (e.g., three vs five jumps, mean jump height vs greatest jump height) may have been used across studies. Therefore, the influence of the different methods used to quantify jump height on the quality of the reported data is not known and requires further investigation.

Mean CMJ height ranged from 35 to 77 cm across studies in adult male basketball players. Multiple studies reported jump height and peak power of professional, semi-professional, and collegiate players. Findings demonstrated heterogenous outcomes for jump height and peak power output both within (e.g., professional vs professional) and between (e.g., professional vs collegiate) competition levels. The lack of research at the representative and amateur playing levels limited the ability to draw conclusions regarding CMJ performance for players competing at these levels. The variation in CMJ height and peak power output observed in professional, semi-professional, and collegiate players may be reflective of the different testing methodologies used across studies combined with the varying abilities of players assessed across different competition levels. When mean CMJ height was reported according to playing position irrespective of competition level, similar ranges were observed (guards: 38–60 cm, forwards: 36–58 cm, and centres: 36–57 cm). From a methodological perspective, when conducting a CMJ on a force platform, basketball practitioners are able to record and track variables that are sensitive to changes over time (e.g., relative power output [151]), as well as monitor acute changes in jump performance (e.g., height) or strategy (e.g., flight time to contraction time ratio [152]). These varied options for interpretation may be of particular benefit when assessing and monitoring player fatigue and readiness across the season [153].

The SJ was also implemented to assess adult male basketball players throughout the literature but only in professional and collegiate players. Insufficient data were reported at the semi-professional, representative, collegiate, and amateur competition levels to draw conclusions. While professional player SJ height ranged (29–50 cm) across studies. Positional differences in SJ height were reported only at the professional level, with no clear differences apparent and centres exhibiting the least variability (guards: 30–41 cm, forwards: 29–40 cm, centres: 33–36 cm). The jump height attained during the SJ was consistently lower than the CMJ and VJ. These differences are due to the concentric-only force expression of the SJ and the inability to use elastic energy generated during the preparatory countermovement evident in the CMJ and VJ [150]. However, when the SJ and CMJ tests are used together, the combination of outcome variables (e.g., jump height) can be used as a diagnostic tool, allowing basketball practitioners to evaluate the ability of players to use their stretch–shortening cycle while jumping [90]. However, identifying the reliability of these diagnostic variables such as the eccentric utilisation ratio, which can be calculated as CMJ height divided by SJ height (peak power may also be used), requires more research in basketball players.

The high frequency of jumps performed during basketball games has been well established [10, 12]. However, the quantity of different jump types (e.g., stationary jump vs running vertical jump, unilateral vs bilateral take-off), the number of maximal and submaximal jumps, and whether there are differences in the frequency of different jumps between playing positions or competition levels is unknown. This gap in the literature is a limitation when designing training programmes to enhance jump performance in basketball players, as the exact type of jumping demands imposed on players are not fully understood. While it is clear different jump strategies exist, it is important to recognise they are underpinned by different speed-strength qualities (e.g., reactive strength, concentric-only speed strength) [139]. Consequently, to holistically assess jumping ability in players, multiple tests may be required. A battery of tests that target a range of force production strategies such as SJ for concentric only force production, CMJ for long-slow stretch shortening cycle force production, and drop or repeated jumps for short-fast stretch shortening cycle production warrant consideration. Additionally, assessing jump performance from varying approaches and take-off strategies may provide further insight into jumping ability. Reporting arm reach during a jump, or the combination of jump height and wingspan may provide novel insight into the maximal ability of players to secure a rebound or tip the ball to advantage during match-play. The combination of multiple jump tests may enable individual jump-profiles to be developed and allow training programmes to target the unique deficiencies of each player. However, it must be acknowledged that implementing an extensive jump battery may not be practical. Therefore, basketball practitioners may wish to select the most appropriate tests from the provided recommendations that best suits their needs.

Basketball players are frequently required to execute repeated jumps to challenge opposition shots and contest rebounds during match-play. While stationary bilateral jumps provide valuable information regarding the vertical jump ability of players, often multiple jumps are required in quick succession (e.g., multiple jumps while contesting a rebound). Therefore, an assessment of the speed-strength quality that underpins repeated jumping is warranted during testing. The reactive strength index represents reactive jump ability and has traditionally been assessed using drop jumps [154] or repeated jump protocols [96, 155] in male basketball players. However, we propose a novel bilateral hopping test protocol [156, 157] to measure both reactive strength and leg stiffness. While we are unaware of any basketball research that incorporates the bilateral hopping test, reactive strength index and leg stiffness are important qualities in basketball players because of the need to perform repeated jumps during training and matches. Furthermore, the bilateral hopping test has been shown to demonstrate greater between-day reliability compared with other repeated jump tests in adolescent rugby union players (e.g., five repeated jumps in place [157]). Additionally, the bilateral hopping test can be efficiently completed through a single test (compared with drop jump profiles that require multiple jumps) and can be standardised with the use of a metronome to ensure consistency.

#### Linear Sprint Performance

The game demands of basketball require well-developed linear sprint and acceleration capacities [12, 61, 65, 158–160]. Throughout the literature, heterogenous linear sprint distances have been used to assess adult male basketball players. When observing the three most commonly used distances, often insufficient data were observed to draw firm conclusions. Of the studies that reported linear sprint performance across distances up to 20 m, only three [4, 21, 58] also reported 5-m and 10-m splits. Over 10 m, the limited available evidence [4, 27, 79] suggests guards (1.72–2.19 s) and forwards (1.72–2.25 s) possess similar linear sprint speed, and are faster than centres (1.80–2.34 s). Considering the match demands of basketball [7, 11, 12], researchers and practitioners are strongly encouraged to capture split times at the 5-m and 10-m marks during a 20-m linear sprint test. The additional data captured at 5 m and 10 m reflect distances that are encountered during match-play [7, 112] and may provide further insight into the acceleratory ability of players. Furthermore, basketball researchers and practitioners would benefit by reporting sprint times relative to playing position to help establish meaningful position-specific normative data that can assist with determining minimum thresholds for playing standards for adult male basketball players. Additionally, reporting sprint times relative to height may provide novel insight into the sprint capabilities of players and may be appropriate for categorising players who play across multiple positions throughout a match.

#### Change-of-Direction Speed

The ability to rapidly change direction within the confines of the court is important for basketball performance [20, 65, 161, 162]. Change-of-direction speed was most commonly assessed using the Agility *T*-Test. Observations between competition levels suggest change-of-direction speeds are similar between professional (8.84–10.04 s) and collegiate (8.92–9.78 s) players, with slower times evident in semi-professional players (9.52–10.90 s). There were insufficient data to draw conclusions regarding positional differences in Agility *T*-Test performance. Further research is recommended to explore whether differences in change-of-direction speed are apparent between playing positions at other competition levels. However, the Agility *T*-Test has been scrutinised as it has been shown to favour specific physical characteristics such as 10-m linear sprint speed (*r* =  − 0.92) and shuffling speed to the right (*r* =  − 0.75) in semi-professional male basketball players [112]. A further concern of the Agility *T*-Test is the distances covered are not reflective of match requirements in basketball [12, 112]. Consequently, a proposed modified Agility *T*-Test, where the distances are shortened to better reflect match demands players encounter has been suggested as an alternative option to assess change-of-direction speed in basketball players [148]. Nevertheless, this test has only been reported in one study examining adult male basketball players [64], and the validity and utility of a modified Agility *T*-Test as a measure of change-of-direction speed in adult male basketball players is not yet known and warrants further research.

Alternative change-of-direction speed tests such as the Y-Shaped Change-of-Direction Speed Test have also been used to assess adult male basketball players, but only in semi-professional players. Consequently, firm conclusions regarding the efficacy of the Y-Shaped Change-of-Direction Speed Test to discriminate between playing positions and competition levels is unclear. Moreover, a concern of the Y-Shaped Change-of Direction Speed Test is the lack of lateral movements, which are regularly performed in basketball match-play. In recent years, the Lane Agility Test has been used to assess change-of-direction speed at the collegiate level in adult male basketball players [3, 51, 111]. Similar to the Agility *T*-Test, the requirements of the Lane Agility Test are not reflective of most movement tasks commonly required during basketball match-play (i.e., no cognitive or perceptual elements present). Nonetheless, the Lane Agility Test consists of pre-determined periods of accelerating, lateral shuffling, and backwards running, all of which are typical movements in basketball. Mean Lane Agility Test performance was similar between collegiate players (10.4–11.8 s) and data captured at the NBA Draft Combine (10.3–12.2 s). Considering the collegiate playing pathway is a common route to playing professionally in the NBA, basketball researchers and practitioners may benefit from implementing the Lane Agility Test to their testing batteries to familiarise their players with the demands of the NBA Draft Combine if their players intend on entering the NBA draft. However, further exploration to identify the ability of the Lane Agility Test to discriminate change-of-direction speed between playing positions and competition levels is required. These findings suggest change-of-direction speed alone may not yet provide basketball researchers and practitioners with sufficient information to confidently evaluate and discriminate between adult male players competing at different levels.

#### Agility

Basketball match-play requires players to interpret stimuli and rapidly execute an appropriate movement response [20, 149], highlighting the need for a perceptual element to be present when assessing agility [47]. However, physical and technical components such as lower-body strength and movement strategy also contribute to agility performance [46, 47]. The introduction of a decision-making constraint has indicated some agility tests are better able to discriminate between competition levels (semi-professional vs amateur [113]) and playing roles (starters vs non-starters [60]) compared with pre-planned change-of-direction tests in adult male basketball players. Consequently, the perceptual component present during an agility test may be of greater importance in discriminating between players at different competition levels than pre-planned change-of-direction speed. However, it is important to acknowledge that change-of-direction speed and agility are independent skills [46]. Considering the limited amount of research investigating agility in basketball players, further research is needed to explore potential differences between playing positions and competition levels in adult male basketball players and to develop an efficacious and ecologically valid agility test.

Finally, if implementing an agility test in basketball players, the type of stimuli being used should be considered. In football codes, a sport-specific stimulus has been shown to be an important component when assessing agility [163] with players competing at higher levels often performing better than players competing at lower levels in Australian rules football [164, 165] and rugby league [166, 167]. Throughout the literature, timing light systems [113, 118], light-up cone systems [49, 85], and humans who initiate movement [4, 21, 60] were the stimuli identified in agility tests used to assess adult male basketball players. Across studies included in this review, basketball researchers and practitioners emphasised accuracy during trials (i.e., the participant made the correct decision) as ‘important’ [4, 60] as identifying and executing the appropriate movement strategy during match-play may lead to better outcomes (e.g., anticipating the opponents movement and drawing an offensive foul) than if the incorrect decision was made (e.g., being called for a blocking foul due to being out of position rather than successfully drawing an offensive foul). However, the accuracy of the attempts were not always reported [4, 21, 49, 60, 85, 113, 118]. Often if players made the incorrect decision or anticipated the required movement rather than responding to the stimuli during a trial, the attempt was discarded, not included in the data reported, and repeated [113, 118]. Therefore, it is recommended basketball researchers and practitioners report the outcome of agility trials (i.e., successful or unsuccessful) in the future as this may provide greater insight to the decision-making ability of players. The development of an outcome-based assessment of attacking and defending agility in basketball may provide a more comprehensive assessment of agility in adult male basketball players.

#### Strength

Muscular strength is an important quality in basketball players [94, 124, 168–170]. The limited data pertaining to lower-body strength across competition levels suggest professional players (back squat 1RM: 143–202 kg) possess greater lower-body strength than collegiate players (back squat 1RM: 116–156 kg). In collegiate players, lower-body strength has been related to career obtainment, with stronger players reaching higher competition levels than their weaker peers [124]. Additionally, back squat 1RM has been shown to have a strong correlation with playing time in NCAA division 1 (*r* = 0.64) and division 2 (*r* = 0.74) competitions [51, 104]. The limited evidence available inhibits the ability to discern if lower-body strength can discriminate between playing positions in adult male basketball players. Consequently, the current positional demands and minimum physical thresholds of lower-body strength required by each playing position and competition level are unknown in adult male basketball. To address these gaps, basketball researchers and practitioners are encouraged to report strength testing results according to playing position to elucidate any positional differences that may be present.

Upper-body strength is often required in basketball matches when players compete to create and defend space. However, it is important to acknowledge that in order for isolated upper-body strength to transfer to match-play, contributions from force production components of the lower body may be required. The range of 1RM loads lifted during the bench press were similar between professional (70–112 kg) and collegiate (76–102 kg) players, with insufficient data reported for other competition levels. The lack of upper-body strength data observed for players competing at lower competition levels could be attributed to strength testing not being prioritised by basketball researchers and practitioners due to the fatigue induced from testing and level of exercise competency required of players. A possible solution for basketball researchers and practitioners to gather strength data from their players is to use a linear position transducer (LPT) during resistance training sessions to measure kinetic and kinematic outputs [36, 171]. The use of an LPT during strength training can provide valid and reliable performance data [172], which is able to be tracked over time to monitor player progression (e.g., changes in bar speed at a specified load) [171, 173], and used to predict maximal strength whilst inducing minimal fatigue [171, 174, 175]. Only one study [121] reported bench press 1RM relative to playing position in adult male basketball players. Consequently, further research is required to confidently establish the upper-body strength characteristics of each playing position.

The evidence collated in this review indicate that strength testing in adult male basketball players requires further research to fully understand the minimum strength standards required of each playing position and competition level. Nonetheless, it is recommended basketball researchers and practitioners continue testing maximal upper-body and lower-body strength to monitor changes in strength across time. Additionally, the assessment of maximal strength will allow for accurate prescription of resistance training loads (e.g., %1RM). Furthermore, the combination of dynamic and isometric strength tests may allow basketball researchers and practitioners to profile temporal and absolute force production ability in players. While there are numerous isometric tests available to assess force production characteristics, such as the isometric squat and isometric mid-thigh pull (IMTP), there is little published evidence exploring their utility and efficacy in basketball. The IMTP is an isometric test proposed to measure strength and force production characteristics of basketball players, owing to the ease of use and minimal fatigue induced in players [148]. The IMTP may also provide basketball researchers and practitioners with an option to test players who do not have the competency to undertake maximal dynamic strength testing. Furthermore, highly sensitive variables (e.g., early-phase force development across time bands) measured during the IMTP may be used to monitor player fatigue [176]. Therefore, a strength profile comprising the IMTP, bench press, and back squat may allow basketball researchers and practitioners to develop baseline levels of strength that support training prescription and are also able to guide return to play from injury.

#### Anaerobic Capacity

Well-developed anaerobic capacity allows basketball players to repeatedly perform high-intensity movements that are typically separated by brief rest periods during matches [17, 88, 177]. Assessment of anaerobic capacity involved either running or resisted cycling tests. Regarding running tests, full court shuttle run performance was homogenous (27.4–27.8 s) across professional [125], semi-professional [28], and collegiate [51] adult male basketball players. Additional data are needed to determine if positional differences in the full court shuttle run exist and if the test is able to confidently discriminate between competition levels. The RAST was also used to assess anaerobic capacity exclusively in professional players, prohibiting the ability to compare performance between competition levels. Insufficient studies were observed to draw conclusions regarding positional differences in RAST performance in adult male basketball players. Subsequently, further research is required to elucidate any differences in RAST performance by playing position or competition level.

Regarding cycling tests, the WAnT cycle test was primarily used to assess anaerobic capacity and only reported in professional players. This isolated use of the WAnT cycle test in professional teams may be due to higher level organisations having greater access to specialised equipment and expertise to reliably implement this test. Additionally, the time associated with testing players using the WAnT cycle test may be impractical at lower competition levels. Insufficient data were available to draw conclusions regarding positional performance during the WAnT. While the data gathered from the WAnT cycle test provide valuable insight regarding the anaerobic capacity of basketball players, the time and resources required to implement the WAnT are considerable. Furthermore, the transfer of cycling anaerobic power to relevant sustained high-intensity movement patterns in basketball are not known. Therefore, basketball researchers and practitioners are encouraged to continue assessing the anaerobic capacity of adult male basketball players using tests that are accessible and appropriate to their needs. Additionally, reporting outcome variables indicative of anaerobic capacity relative to playing position using data in absolute terms and relative to body mass is recommended.

#### Aerobic Capacity

Basketball players require well-developed aerobic capacities to tolerate the intermittent bouts of varying intensity encountered during matches [129, 155, 178–181]. Players with a high aerobic capacity are better able to tolerate multiple high-intensity sprints and have improved fatigue resistance [182]. Throughout the literature, mean estimated and measured VO_2max_ ranged from 42 to 64 mL/kg/min across studies in adult male basketball players. This variation in results may be attributed to different tests being adopted (e.g., MSFT vs Yo-Yo IRL1 vs incremental treadmill test) and the inherent levels of error when calculating VO_2max_ during each test [183].

The use of incremental treadmill tests was evident at professional (50–61 mL/kg/min) and collegiate (50–58 mL/kg/min) levels and revealed similar well-developed mean aerobic capacity across both competition levels. Insufficient data were observed at the semi-professional, representative, and amateur levels to draw conclusions regarding the incremental treadmill test. Positional comparisons indicated guards possess the greatest mean aerobic capacity across studies in professional players (50–60 mL/kg/min), then forwards (46–58 mL/kg/min), followed by centres (42–58 mL/kg/min). The positional differences in aerobic capacity may be attributed to the unique match roles required of each position. The frequent use of incremental treadmill tests to assess VO_2max_ in professional and collegiate players may be attributed to basketball practitioners at these levels having greater access to resources such as laboratory-based physiological testing equipment than lower levels. Furthermore, professional and collegiate players may have a greater availability for testing throughout the year compared with other competition levels (e.g., semi-professional players may have competing demands such as supplementary jobs).

In a practical setting, the ability to test players efficiently is an important consideration for basketball researchers and practitioners, and the ability to test multiple athletes simultaneously is often advantageous. A variety of running-based tests that are able to assess multiple players at once were identified in the literature. The two most commonly used tests were the Yo-Yo IRL1 and the MSFT, with the Yo-Yo IRL1 used most frequently at the professional and semi-professional levels. In contrast, the MSFT was used mainly in professional players. Positional differences in VO_2max_ attained during the MSFT reflect a similar trend to aerobic capacity from incremental treadmill tests, with professional guards recording the greatest estimated VO_2max_ (45–64 mL/kg/min), then forwards (43–62 mL/kg/min), and centres (42–58 mL/kg/min). However, insufficient data were available to compare MSFT performance across competition levels and to identify differences in VO_2max_ between playing positions using the Yo-Yo IRL1. Therefore, further research is required to contribute meaningful data for the development of normative standards regarding the aerobic capacity requirements according to playing position and competition level in adult male basketball players.

## Limitations

While this review presents a contemporary and comprehensive analysis of basketball tests and reveals the physical characteristics of adult male basketball players, there are limitations that should be considered. First, this review excluded tests involving a basketball-specific skill component (e.g., dribbling or shooting) and tests that assessed several physical characteristics simultaneously (e.g., Basketball Exercise Simulation Test [137, 138]). While these tests may offer novel insight regarding basketball-related fitness, they are often assessing multiple physical characteristics at the same time, and therefore were not considered in this review. Second, the interaction between physical characteristics, psychological influences, technical abilities, tactical abilities, and the competitive environment in relation to basketball performance was not investigated. Thus, discriminating between competition levels or selecting players principally based on their physical characteristics is cautioned, as enhanced physical characteristics alone do not guarantee that a player will be successful. Furthermore, it is important to acknowledge that varying levels of inherent natural ability, underpinned largely by genetic components exist. However, there are elements of fitness such as fatigue resistance and muscular endurance that are able to be enhanced by appropriate training. Finally, due to the heterogeneity of testing methods reported in the literature, we were unable to perform a meta-analysis of physical characteristics across playing positions and competition levels.

## Practical Recommendations and Considerations for Testing Physical Characteristics

**Fig. 5:**
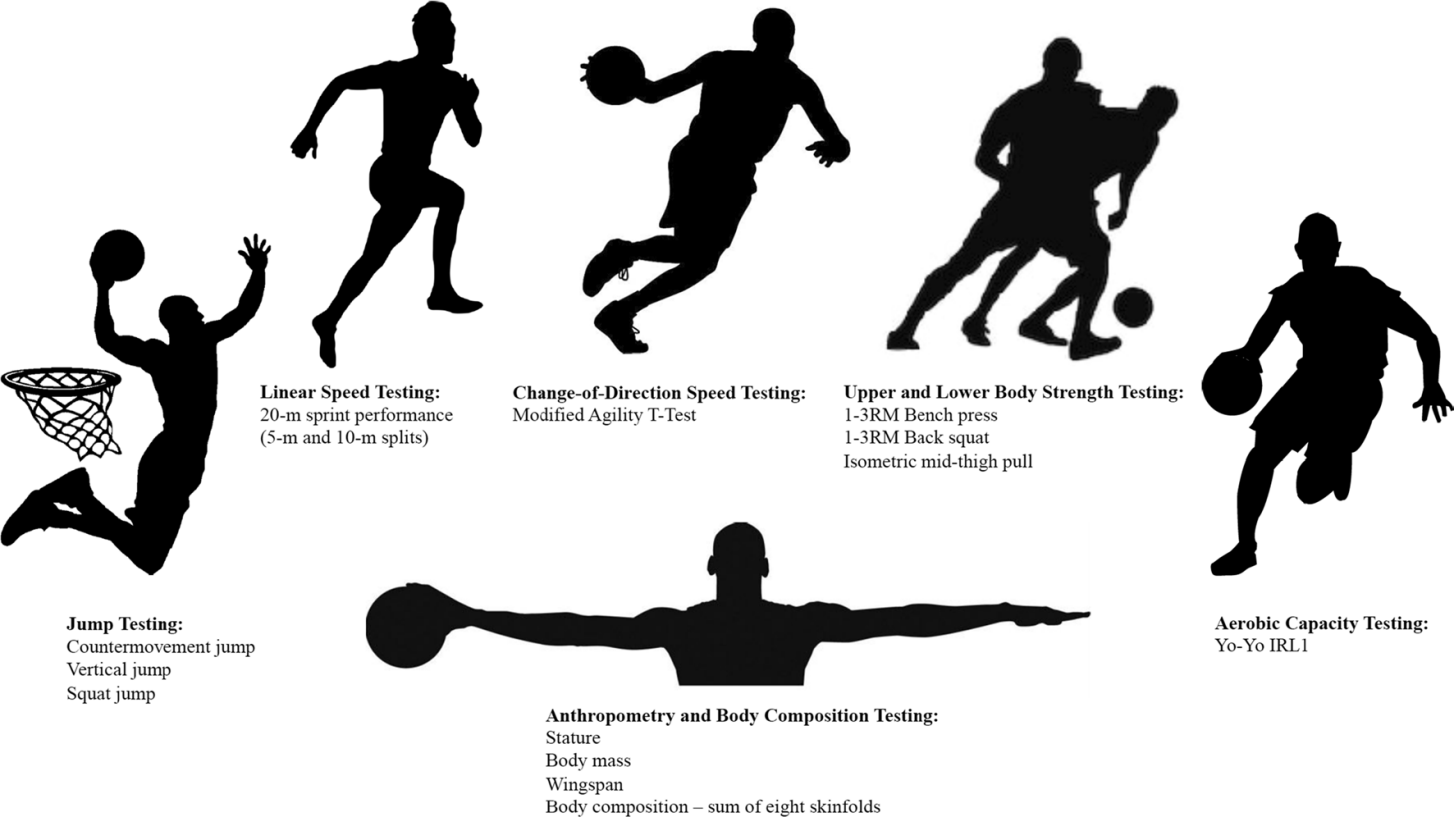
Recommendations for testing the physical characteristics of adult male basketball players.

With the wide range of tests and outcome variables available to basketball researchers and practitioners, developing a testing battery that is both valid and reliable but also informative and efficient can be challenging and at times contentious. Establishing a universal standardised test battery is further complicated by constraints such as resource availability, access to players, and competition or travel schedules that may interfere with scheduling testing sessions. While acknowledging these challenges, testing recommendations and outcome variables for each physical characteristic have been provided (Fig. 5 and Table 13). The proposed battery aims to allow for the standardisation of testing and the implementation of a reproducible protocol that can be used to inform subsequent training practice. Furthermore, the recommended tests are selected based on their efficiency (i.e., ability to test multiple players simultaneously or in succession) and the ability to use variables from multiple tests to infer additional qualities (e.g., sprint momentum, eccentric utilisation ratio). Finally, the testing battery is aimed to be applicable for the real-world assessment of adult male basketball players while drawing upon the scientific literature. It should be noted that this battery is not an exhaustive list of tests and outcome variables, and basketball researchers and practitioners are recommended to add or remove tests and output variables as they see fit provided their decisions are guided by logic, rationale, and data.

**Table 13:** Recommended tests and outcome variables to assess the physical characteristics of adult male basketball players

Characteristic	Test	Outcome variable	Technical note	Citation count
Anthropometry	Height (cm)		Measuring anthropometry at the beginning of the testing battery is recommended while players are in a rested state	114
	Body mass (kg)			114
	Wingspan (cm)			3
Body composition	Eight-site skinfolds	Sum of eight skinfolds (mm)	High inter-tester variability exists. To gather reliable results, an experienced tester should conduct this test. Preferably, the same practitioner is recommended to administer this test on different occasions. An estimation equation specific to the population is recommended. Sites used should be reported and used consistently	2
Muscular power	Countermovement jump	Jump height (cm) Mean concentric relative power (W/kg)	To reduce error introduced with movement strategies, it is recommended that height is calculated using impulse from a force platform. Relative mean power can be used as an auxiliary to support interpretation of jump data	46
	Vertical jump	Jump height (cm) Mean concentric relative power (W/kg)	See above	33
	Squat jump	Jump height (cm) Mean concentric relative power (W/kg)	Must carefully monitor force–time output to ensure no preparatory countermovement is used. Squat jump height can be coupled with countermovement jump height to support monitoring and prescription through calculation of the ‘eccentric utilisation ratio’	19
Linear speed	20-m sprint	Time intervals (s) Momentum (kg⋅m/s)	Performance time measured at 5-m, 10-m and 20-m intervals. Acceleration over each interval should be calculated. Starting position must be standardised (e.g. 50 cm from first interval)	20
Change-of-direction speed	Modified agility *T*-test	Time (s)	Total distance covered is 20 m, with no more than 5 m covered before requiring a change of direction. Player must remain facing forwards for the duration of the test	1
Strength	1–3RM Back squat	Absolute load (kg) Relative strength Load-velocity profile	Players must be competent with technique to safely implement test. Squat depth must be standardised. Additionally, during sub-maximal efforts, a linear position transducer should be used to develop individualised load-velocity profiles. Dependent upon athlete technical proficiency, an isometric mid-thigh pull may be an alternate or supplementary test	8
	1–3RM Bench press	Absolute load (kg) Relative strength Load-velocity profile	Players must be competent with the technique to safely implement test. Bench press range of motion must be standardised. Load-velocity profiles should be developed from sub-maximal loads using a linear position transducer	14
Aerobic capacity	Yo-Yo IRL1	Finishing level Estimated VO_2max_ (mL/kg/min)	Yo-Yo IRL1 must be completed on the same flooring (e.g. court) and in similar environmental conditions (e.g. consistent temperature)	12

*RM* repetition maximum, *VO*_*2max*_ maximum oxygen uptake, *Yo-Yo IRL1* Yo-Yo Intermittent Recovery Test Level 1

It is undeniable that anthropometry is an important consideration for basketball players [10, 32, 33, 184–192]. Thus, the measurement of height, body mass, and wingspan are strongly recommended at the beginning of testing. Following these assessments, the sum of eight skinfolds, reported in mm, is suggested because of the low associated cost, relative ease of implementation, and the ability to gather reliable results provided tester competency as outlined by Kasper et al. [147]. With the high prevalence of jumping during basketball match-play [12, 20], a range of jumping tests have been recommended, including the CMJ, VJ, SJ, and the bilateral hopping test, with each jump test assessing different characteristics. For measures of linear sprint speed and change-of-direction speed, 20-m linear sprints and a modified Agility *T*-Test are recommended, respectively. Because of the relatively short length of a basketball court (~ 28 m), a 20-m sprint with 5-m and 10-m split times also recorded may be more appropriate to assess linear sprint performance compared with tests across longer distances that have been adopted in some studies (e.g., 40 m [56, 93]). The modified Agility *T*-Test is recommended as the distances covered during the test are limited to 5 m in any direction, before requiring a change of direction. The shorter distances are more reflective of match demands compared with the traditional Agility *T*-Test [112]. To assess agility, further research is required to develop an agility test that is repeatable, practical to implement, and reflects the demands of basketball. The Y-shaped Agility test may provide basketball practitioners with insight into the agility of players, although the movement demands are not specific to basketball match-play. Therefore, a definitive agility test for use in adult male basketball players cannot be recommended at this stage.

For measures of strength, 1–3RM during the back squat and bench press are suggested given their ability to provide data that can be monitored over time, inform training prescription, and provide baseline strength measurements for return to play protocols following injury. While the need to produce force is undoubtedly important in the production of power, it may also help support players in maintaining or securing an advantageous position during matches (e.g., backing down an opponent in the low post). It is also recommended that during strength testing, as players are building towards their maximal effort, the velocity of submaximal loads are monitored with an LPT [36]. Use of linear position transducers will support the development of load-velocity profiles in players, which can be used to assess changes in force production with submaximal loads, enhance training prescription, and monitor fatigue [171]. It should also be noted that an IMTP could be a feasible alternative if movement proficiency and competency during common resistance training exercises is lacking. Because of the variability amongst anaerobic tests in the literature, no definitive anaerobic capacity test has been recommended. However, if researchers and practitioners wish to assess anaerobic capacity, an ecologically valid and reliable test should be considered. Finally, the use of the Yo-Yo IRL1 has been suggested to assess aerobic capacity due to its validity, reliability, and feasibility [193, 194]. It is acknowledged that the gold standard gas analysis may provide improved accuracy in measuring VO_2max_; however, because of the constraints commonly associated with this testing (e.g., predominantly in laboratory settings, time, cost, non-basketball-specific movement patterns in test protocols), an efficient on-court solution has been recommended that has been repeatedly used in the literature to assess several samples of adult male basketball players [28, 65, 67, 72, 137].

## Conclusions

This review collates all tests and outcome variables used to assess the physical characteristics of adult male basketball players in the literature to date. The number of tests and outcome variables identified confirm that a gold-standard testing battery for assessing the physical characteristics of basketball players does not exist. While it appears basketball practitioners are prioritising the assessment of specific physical characteristics (i.e., anthropometrics, muscular power, linear speed, change-of-direction speed, agility, strength, anaerobic capacity, and anaerobic capacity), the methods of assessment often vary in regard to technology used (e.g., force platform vs jump mat), variables reported (e.g., mean jump height from multiple attempts *vs.* peak jump height) and test protocols implemented (e.g., number of jumps permitted during a jump test). Further, the varying levels of inherent validity and reliability across the spectrum of tests reported make the establishment of normative data challenging and the comparison of physical characteristics across studies difficult to make in basketball players. To develop meaningful normative data, basketball practitioners must develop standardised testing protocols that are reproducible and reflective of match demands. Developing league-wide and federation-wide testing batteries would allow for the longitudinal assessment of players in large cohorts and the establishment of minimum physical standards for playing positions and competition levels.

## Supplementary Information

Below is the link to the electronic supplementary material.

Supplementary file1 (DOCX 114 kb)

## Declarations

### Availability of data and material

All data and materials reported in this systematic review are from peer-reviewed publications and publicly available data from official organisation websites (https://www.nba.com/stats/draft/combine/). All of the extracted data are included in the manuscript and supplementary files.
