# Supplementary material for: A Systematic Review on Fitness Testing in Adult Male Basketball Players: Tests Adopted, Characteristics Reported and Recommendations for Practice
Source: Sports Med. 2022 Feb 4;52(7):1491–532. doi: 10.1007/s40279-021-01626-3 (PMC9213321; doi:10.1007/s40279-021-01626-3)
Supplement: Supplementary file 1 — Supplementary file1 (DOCX 114 kb) [file 40279_2021_1626_MOESM1_ESM.docx]

**A Systematic Review on Fitness Testing in Adult Male Basketball Players: Tests Adopted, Characteristics Reported and Recommendations for Practice**

*Running Head: Testing methods and physical characteristics of male basketball players*

Matthew Morrison^1^, David T. Martin^1^, Scott Talpey^2^, Aaron T. Scanlan^3^, Jace Delaney, Shona L. Halson^1,4^, Jonathon Weakley^1,4,5^

1. School of Behavioural and Health Sciences, Australian Catholic University, Brisbane, Australia
2. School of Science, Psychology and Sport, Federation University Australia, Ballarat, Australia
3. Human Exercise and Training Laboratory, School of Health, Medical and Applied Sciences, Central Queensland University, Rockhampton, Australia
4. Sports Performance, Recovery, Injury and New Technologies (SPRINT) Research Centre, Australian Catholic University, Brisbane, QLD, Australia
5. Carnegie Applied Rugby Research (CARR) Centre, Institute of Sport, Physical Activity and Leisure, Leeds Beckett University, Leeds, UK

**Corresponding author:**

Building 211.1.16

School of Behavioural and Health Sciences

Australian Catholic University

Brisbane, Queensland, Australia

Email: Jonathon.weakley@acu.edu.au

| **Supplementary Table 1.** Downs and Black assessment of included studies reporting quality | | | | | | | | | | | | | |
| --- | --- | --- | --- | --- | --- | --- | --- | --- | --- | --- | --- | --- | --- |
| **Study** | **Question number** | | | | | | | | | | | | **Total score** |
|  | 1 | 2 | 3 | 4 | 6 | 7 | 10 | 11 | 12 | 16 | 18 | 20 |  |
| Ahmed [31] | 1 | 1 | 1 | 1 | 1 | 1 | 1 | 0 | 0 | 1 | 1 | 1 | 10 |
| Alejandro et al. [176] | 1 | 1 | 1 | 1 | 1 | 1 | 0 | 0 | 0 | 1 | 0 | 1 | 8 |
| Alemdaroğlu [60] | 1 | 1 | 1 | 1 | 1 | 1 | 0 | 0 | 0 | 1 | 1 | 1 | 9 |
| Angyan et al. [43] | 1 | 1 | 1 | 1 | 1 | 1 | 1 | 0 | 0 | 1 | 1 | 1 | 10 |
| Annino et al. [61] | 1 | 1 | 1 | 1 | 1 | 1 | 1 | 0 | 0 | 1 | 1 | 1 | 10 |
| Aoki et al. [62] | 1 | 1 | 1 | 1 | 1 | 1 | 1 | 0 | 0 | 1 | 1 | 1 | 10 |
| Asadi et al. [96] | 1 | 1 | 1 | 1 | 1 | 1 | 0 | 0 | 0 | 1 | 1 | 1 | 9 |
| Balabinis et al. [97] | 1 | 1 | 1 | 1 | 1 | 1 | 0 | 0 | 0 | 1 | 1 | 1 | 9 |
| Balsalobre-Fernandez et al. [99] | 1 | 1 | 1 | 1 | 1 | 1 | 1 | 0 | 0 | 1 | 1 | 1 | 10 |
| Balsalobre-Fernandez et al. [98] | 1 | 1 | 1 | 1 | 1 | 1 | 1 | 0 | 0 | 1 | 1 | 1 | 10 |
| Barrera-Domínguez et al. [63] | 1 | 1 | 1 | 1 | 1 | 1 | 1 | 0 | 0 | 1 | 1 | 1 | 10 |
| Ben Abdelkrim et al. [64] | 1 | 1 | 1 | 1 | 1 | 1 | 0 | 0 | 0 | 1 | 1 | 1 | 9 |
| Bolonchuk et al. [128] | 1 | 1 | 0 | 1 | 1 | 1 | 1 | 0 | 0 | 1 | 1 | 1 | 9 |
| Boone & Bourgois [27] | 1 | 1 | 1 | 1 | 1 | 1 | 1 | 0 | 0 | 1 | 1 | 1 | 10 |
| Boone et al. [129] | 1 | 1 | 1 | 1 | 1 | 1 | 1 | 0 | 0 | 1 | 1 | 1 | 10 |
| Bradic et al. [23] | 1 | 1 | 1 | 1 | 1 | 1 | 1 | 0 | 0 | 1 | 1 | 1 | 10 |
| Buśko [24] | 1 | 1 | 1 | 1 | 1 | 1 | 0 | 0 | 0 | 1 | 1 | 1 | 9 |
| Buśko et al. [65] | 1 | 1 | 1 | 1 | 1 | 1 | 0 | 0 | 0 | 1 | 1 | 1 | 9 |
| Cabarkapa et al. [123] | 1 | 1 | 1 | 1 | 1 | 1 | 0 | 0 | 0 | 1 | 1 | 1 | 9 |
| Castagna et al. [151] | 1 | 1 | 1 | 1 | 1 | 1 | 1 | 0 | 0 | 1 | 1 | 1 | 10 |
| Caterisano et al. [53] | 1 | 1 | 0 | 1 | 1 | 1 | 0 | 0 | 0 | 1 | 1 | 1 | 8 |
| Chaouachi et al. [66] | 1 | 1 | 1 | 1 | 1 | 1 | 1 | 0 | 0 | 1 | 1 | 1 | 10 |
| Chatzinikolaou et al. [130] | 1 | 1 | 1 | 1 | 1 | 1 | 0 | 0 | 0 | 1 | 1 | 1 | 9 |
| Chen et al. [67] | 1 | 1 | 1 | 1 | 1 | 1 | 0 | 0 | 0 | 1 | 1 | 1 | 9 |
| Ciacci & Bartolomei [95] | 1 | 1 | 1 | 1 | 1 | 1 | 0 | 0 | 0 | 1 | 0 | 1 | 8 |
| Cormery et al. [15] | 1 | 1 | 1 | 1 | 1 | 1 | 1 | 0 | 0 | 1 | 1 | 1 | 10 |
| Dawes & Spiteri [51] | 1 | 1 | 0 | 1 | 1 | 1 | 1 | 0 | 0 | 1 | 1 | 1 | 9 |
| de Araujo et al. [127] | 1 | 1 | 1 | 1 | 1 | 1 | 0 | 0 | 0 | 1 | 1 | 1 | 9 |
| de Sousa Fortes et al. [58] | 1 | 1 | 1 | 1 | 1 | 1 | 1 | 0 | 0 | 1 | 1 | 1 | 10 |
| Delextrat & Cohen [113] | 1 | 1 | 1 | 1 | 1 | 1 | 0 | 0 | 0 | 1 | 1 | 1 | 9 |
| Delextrat et al. [169] | 1 | 1 | 1 | 1 | 1 | 1 | 1 | 0 | 0 | 1 | 1 | 1 | 10 |
| Dello Iacono et al. [68] | 1 | 1 | 1 | 1 | 1 | 1 | 1 | 0 | 0 | 1 | 1 | 1 | 10 |
| Dragonea et al. [131] | 1 | 1 | 1 | 1 | 1 | 1 | 1 | 0 | 0 | 1 | 1 | 1 | 10 |
| Erkmen et al. [17] | 1 | 1 | 1 | 1 | 1 | 1 | 1 | 0 | 0 | 1 | 1 | 1 | 10 |
| Fatouros et al. [124] | 1 | 1 | 1 | 1 | 1 | 1 | 0 | 0 | 0 | 1 | 1 | 1 | 9 |
| Ferioli et al. [54] | 1 | 1 | 1 | 1 | 1 | 1 | 1 | 0 | 0 | 1 | 1 | 1 | 10 |
| Ferioli et al. [134] | 1 | 1 | 1 | 1 | 1 | 1 | 1 | 0 | 0 | 1 | 1 | 1 | 10 |
| Ferioli et al. [16] | 1 | 1 | 1 | 1 | 1 | 1 | 1 | 0 | 0 | 1 | 1 | 1 | 10 |
| Ferioli et al. [157] | 1 | 1 | 1 | 1 | 1 | 1 | 1 | 0 | 0 | 1 | 1 | 1 | 10 |
| Ferioli et al. [55] | 1 | 1 | 1 | 1 | 1 | 1 | 1 | 0 | 0 | 1 | 1 | 1 | 10 |
| Freitas et al. [69] | 1 | 1 | 1 | 1 | 1 | 1 | 0 | 0 | 0 | 1 | 1 | 1 | 9 |
| Freitas et al. [70] | 1 | 1 | 1 | 1 | 1 | 1 | 1 | 0 | 0 | 1 | 1 | 1 | 10 |
| Fields et al. [50] | 1 | 1 | 1 | 1 | 1 | 1 | 1 | 0 | 0 | 1 | 1 | 1 | 10 |
| Fujii et al. [154] | 1 | 1 | 0 | 1 | 1 | 1 | 1 | 0 | 0 | 1 | 1 | 1 | 9 |
| Gaurav et al. [177] | 1 | 1 | 0 | 1 | 1 | 1 | 1 | 0 | 0 | 1 | 0 | 1 | 8 |
| Gerodimos et al. [178] | 1 | 1 | 1 | 1 | 1 | 1 | 0 | 0 | 0 | 1 | 1 | 1 | 9 |
| Gerodimos et al. [140] | 1 | 1 | 1 | 1 | 1 | 1 | 0 | 0 | 0 | 1 | 1 | 1 | 9 |
| Gillam [118] | 1 | 1 | 1 | 1 | 0 | 1 | 0 | 0 | 0 | 1 | 1 | 1 | 8 |
| Gocentas et al. [18] | 1 | 1 | 1 | 1 | 1 | 1 | 1 | 0 | 0 | 1 | 1 | 1 | 10 |
| Gocentas et al. [19] | 1 | 1 | 1 | 1 | 1 | 1 | 1 | 0 | 0 | 1 | 1 | 1 | 10 |
| Gomes et al. [71] | 1 | 1 | 1 | 1 | 1 | 1 | 1 | 0 | 0 | 1 | 1 | 1 | 10 |
| Gonzalez et al. [122] | 1 | 1 | 1 | 1 | 1 | 0 | 1 | 0 | 0 | 1 | 1 | 1 | 9 |
| Gryko et al. [52] | 1 | 1 | 1 | 1 | 1 | 1 | 1 | 0 | 0 | 1 | 1 | 1 | 10 |
| Hadzic et al. [25] | 1 | 1 | 1 | 1 | 1 | 1 | 1 | 0 | 0 | 1 | 1 | 1 | 10 |
| Harbili [128] | 1 | 1 | 1 | 1 | 1 | 1 | 1 | 0 | 0 | 1 | 1 | 1 | 10 |
| Haugen et al. [155] | 1 | 1 | 0 | 1 | 1 | 1 | 1 | 0 | 0 | 1 | 1 | 1 | 9 |
| Heishman et al. [72] | 1 | 1 | 1 | 1 | 1 | 1 | 1 | 0 | 0 | 1 | 1 | 1 | 10 |
| Heishman et al. [73] | 1 | 1 | 1 | 1 | 1 | 1 | 1 | 0 | 0 | 1 | 1 | 1 | 10 |
| Heishman et al. [74] | 1 | 1 | 1 | 1 | 1 | 1 | 1 | 0 | 0 | 1 | 1 | 1 | 10 |
| Hoffman et al. [102] | 1 | 1 | 1 | 1 | 1 | 1 | 0 | 0 | 0 | 1 | 1 | 0 | 8 |
| Hoffman et al. [101] | 1 | 1 | 1 | 1 | 1 | 1 | 0 | 0 | 0 | 1 | 1 | 1 | 9 |
| Hoffman et al. [103] | 1 | 1 | 0 | 1 | 1 | 1 | 1 | 0 | 0 | 1 | 1 | 1 | 9 |
| Hunter & Hilyer [119] | 1 | 1 | 1 | 1 | 1 | 1 | 1 | 0 | 0 | 1 | 1 | 0 | 9 |
| Hunter et al. [104] | 1 | 1 | 0 | 1 | 1 | 1 | 1 | 0 | 0 | 1 | 0 | 1 | 8 |
| Ilie & Cristian [164] | 1 | 1 | 0 | 1 | 1 | 0 | 0 | 0 | 0 | 1 | 0 | 1 | 6 |
| Ivanovic & Dopsaj [165] | 1 | 1 | 1 | 1 | 1 | 1 | 1 | 0 | 0 | 1 | 1 | 1 | 10 |
| Jallai et al. [76] | 1 | 0 | 1 | 1 | 1 | 1 | 1 | 0 | 0 | 1 | 1 | 1 | 9 |
| Jeffries et al. [117] | 1 | 1 | 1 | 1 | 1 | 1 | 1 | 0 | 0 | 1 | 1 | 1 | 10 |
| Kariyawasam et al. [56] | 1 | 1 | 1 | 1 | 1 | 1 | 1 | 0 | 0 | 1 | 1 | 1 | 10 |
| Khlifa et al. [77] | 1 | 1 | 1 | 1 | 1 | 1 | 0 | 0 | 0 | 1 | 1 | 1 | 9 |
| Kipp et al. [105] | 1 | 1 | 1 | 1 | 1 | 1 | 1 | 0 | 0 | 1 | 1 | 1 | 10 |
| Köklü et al. [78] | 1 | 1 | 1 | 1 | 1 | 1 | 0 | 0 | 0 | 1 | 1 | 1 | 9 |
| Korkmaz & Karahan [106] | 1 | 1 | 1 | 1 | 1 | 1 | 1 | 0 | 0 | 1 | 1 | 1 | 10 |
| Laplaud et al. [79] | 1 | 1 | 1 | 1 | 1 | 1 | 1 | 0 | 0 | 1 | 1 | 1 | 10 |
| Lehnert et al. [107] | 1 | 1 | 1 | 1 | 1 | 1 | 1 | 0 | 0 | 1 | 1 | 1 | 10 |
| Lockie et al. [3] | 1 | 1 | 1 | 1 | 1 | 1 | 1 | 0 | 0 | 1 | 1 | 1 | 10 |
| Lockie et al. [112] | 1 | 1 | 1 | 1 | 1 | 1 | 1 | 0 | 0 | 1 | 1 | 1 | 10 |
| Lysenko [172] | 0 | 1 | 0 | 1 | 1 | 1 | 0 | 0 | 0 | 1 | 0 | 1 | 6 |
| Maffiuletti et al. [80] | 1 | 1 | 1 | 1 | 1 | 1 | 1 | 0 | 0 | 1 | 1 | 1 | 10 |
| Maggioni et al. [58] | 1 | 1 | 1 | 1 | 1 | 1 | 1 | 0 | 0 | 1 | 1 | 1 | 10 |
| Mandic et al. [81] | 1 | 1 | 1 | 1 | 1 | 1 | 1 | 0 | 0 | 1 | 1 | 1 | 10 |
| Mangine et al. [26] | 1 | 1 | 1 | 1 | 1 | 1 | 1 | 0 | 0 | 1 | 1 | 1 | 10 |
| Markwick et al. [150] | 1 | 1 | 1 | 1 | 1 | 1 | 1 | 0 | 0 | 1 | 1 | 1 | 10 |
| Masanovic et al. [179] | 1 | 1 | 1 | 1 | 1 | 1 | 1 | 0 | 0 | 1 | 1 | 1 | 10 |
| Mathur et al. [180] | 1 | 1 | 1 | 1 | 1 | 1 | 0 | 0 | 0 | 1 | 1 | 1 | 9 |
| McInnes et al. [132] | 1 | 1 | 1 | 1 | 1 | 1 | 1 | 0 | 0 | 1 | 1 | 1 | 10 |
| Metaxas et al. [133] | 1 | 1 | 1 | 1 | 1 | 1 | 0 | 0 | 0 | 1 | 1 | 1 | 9 |
| Mikolajec et al. [156] | 1 | 1 | 0 | 1 | 1 | 0 | 1 | 0 | 0 | 1 | 1 | 1 | 8 |
| Milanović et al. [45] | 1 | 1 | 0 | 1 | 1 | 1 | 1 | 0 | 0 | 1 | 1 | 1 | 9 |
| Mitić et al. [114] | 1 | 1 | 0 | 0 | 1 | 0 | 1 | 0 | 0 | 1 | 1 | 1 | 7 |
| Miura et al. [82] | 1 | 1 | 1 | 1 | 1 | 1 | 1 | 0 | 0 | 1 | 1 | 1 | 10 |
| Montgomery et al. [28] | 1 | 1 | 1 | 1 | 1 | 1 | 1 | 0 | 0 | 1 | 1 | 1 | 10 |
| Muratovic et al. [181] | 1 | 1 | 1 | 1 | 1 | 1 | 1 | 0 | 0 | 1 | 1 | 1 | 10 |
| Myles et al. [115] | 1 | 1 | 1 | 1 | 1 | 1 | 1 | 0 | 0 | 1 | 1 | 1 | 10 |
| Narazaki et al. [57] | 1 | 1 | 1 | 1 | 1 | 1 | 0 | 0 | 0 | 1 | 1 | 1 | 9 |
| Nikolaidis et al. [108] | 1 | 1 | 1 | 1 | 1 | 1 | 1 | 0 | 0 | 1 | 1 | 1 | 10 |
| Omorczyk et al. [121] | 1 | 1 | 1 | 0 | 1 | 1 | 1 | 0 | 0 | 1 | 1 | 1 | 9 |
| Ostojic et al. [83] | 1 | 1 | 1 | 1 | 1 | 1 | 0 | 0 | 0 | 1 | 1 | 1 | 9 |
| Papanikolaou et al. [170] | 1 | 1 | 1 | 1 | 1 | 1 | 1 | 0 | 0 | 1 | 1 | 1 | 10 |
| Parr et al. [120] | 0 | 1 | 1 | 0 | 1 | 1 | 0 | 0 | 0 | 1 | 0 | 1 | 6 |
| Pehar et al. [48] | 1 | 1 | 1 | 1 | 1 | 1 | 1 | 0 | 0 | 1 | 1 | 1 | 10 |
| Pehar et al. [84] | 1 | 1 | 1 | 1 | 1 | 1 | 1 | 0 | 0 | 1 | 1 | 1 | 10 |
| Petway et al. [44] | 1 | 1 | 1 | 1 | 1 | 1 | 1 | 0 | 0 | 1 | 1 | 1 | 10 |
| Pliauga et al. [85] | 1 | 1 | 1 | 1 | 1 | 1 | 1 | 0 | 0 | 1 | 1 | 1 | 10 |
| Pliauga et al. [39] | 1 | 1 | 1 | 1 | 1 | 1 | 1 | 0 | 0 | 1 | 1 | 1 | 10 |
| Pojskić et al. [86] | 1 | 1 | 1 | 1 | 1 | 1 | 1 | 0 | 0 | 0 | 1 | 1 | 9 |
| Pojskić et al. [87] | 1 | 1 | 1 | 1 | 1 | 1 | 1 | 0 | 0 | 1 | 1 | 1 | 10 |
| Pojskić et al. [88] | 1 | 1 | 1 | 1 | 1 | 1 | 1 | 0 | 0 | 0 | 1 | 1 | 9 |
| Ponce-Gonzalez et al. [89] | 1 | 1 | 1 | 1 | 1 | 1 | 1 | 0 | 0 | 1 | 1 | 1 | 10 |
| Poole et al. [111] | 1 | 1 | 1 | 1 | 1 | 1 | 1 | 0 | 0 | 1 | 1 | 1 | 10 |
| Popadic Gacesa et al. [126] | 1 | 1 | 1 | 1 | 1 | 1 | 0 | 0 | 0 | 1 | 1 | 1 | 9 |
| Popovic et al. [182] | 1 | 1 | 1 | 1 | 1 | 1 | 1 | 0 | 0 | 1 | 1 | 1 | 10 |
| Puente et al. [14] | 1 | 1 | 1 | 1 | 1 | 1 | 0 | 0 | 0 | 1 | 1 | 1 | 9 |
| Ramirez-Campillo et al. [29] | 1 | 1 | 1 | 1 | 1 | 1 | 1 | 0 | 0 | 1 | 1 | 1 | 10 |
| Rauch et al. [109] | 1 | 1 | 1 | 1 | 1 | 1 | 1 | 0 | 0 | 1 | 1 | 1 | 10 |
| Rodriguez-Rosell et al. [90] | 1 | 1 | 1 | 1 | 1 | 1 | 1 | 0 | 0 | 1 | 1 | 1 | 10 |
| Sallet et al. [125] | 1 | 1 | 1 | 1 | 1 | 1 | 1 | 0 | 0 | 1 | 1 | 1 | 10 |
| Sanfilippo et al. [183] | 1 | 1 | 1 | 1 | 1 | 1 | 1 | 0 | 0 | 1 | 1 | 1 | 10 |
| Scanlan et al. [136] | 1 | 1 | 1 | 1 | 1 | 1 | 1 | 0 | 0 | 1 | 1 | 1 | 10 |
| Scanlan et al. [135] | 1 | 1 | 1 | 1 | 1 | 1 | 1 | 0 | 0 | 1 | 1 | 1 | 10 |
| Scanlan et al. [21] | 1 | 1 | 1 | 1 | 1 | 1 | 1 | 0 | 0 | 1 | 1 | 1 | 10 |
| Scanlan et al. [4] | 1 | 1 | 1 | 1 | 1 | 1 | 1 | 0 | 0 | 1 | 1 | 1 | 10 |
| Scanlan et al. [59] | 1 | 1 | 1 | 1 | 1 | 1 | 1 | 0 | 0 | 1 | 1 | 1 | 10 |
| Scanlan et al. [30] | 1 | 1 | 1 | 1 | 1 | 1 | 1 | 0 | 0 | 1 | 1 | 1 | 10 |
| Schiltz et al. [91] | 1 | 1 | 1 | 1 | 1 | 1 | 1 | 0 | 0 | 1 | 1 | 1 | 10 |
| Sekulic et al. [49] | 1 | 1 | 1 | 1 | 1 | 1 | 1 | 0 | 0 | 1 | 1 | 1 | 10 |
| Shalfawi et al. [92] | 1 | 1 | 1 | 1 | 1 | 1 | 1 | 0 | 0 | 1 | 1 | 1 | 10 |
| Soslu et al. [116] | 1 | 1 | 1 | 1 | 1 | 1 | 0 | 0 | 0 | 1 | 1 | 1 | 9 |
| Spenst et al. [184] | 1 | 1 | 1 | 1 | 1 | 1 | 1 | 0 | 0 | 1 | 1 | 1 | 10 |
| Stojanovic et al. [93] | 1 | 1 | 1 | 1 | 1 | 1 | 0 | 0 | 0 | 1 | 1 | 1 | 9 |
| Tavino et al. [173] | 1 | 1 | 0 | 1 | 1 | 1 | 0 | 0 | 0 | 1 | 1 | 1 | 8 |
| Theoharopoulos et al. [166] | 1 | 1 | 1 | 1 | 1 | 1 | 1 | 0 | 0 | 1 | 1 | 1 | 10 |
| Tomkinson et al. [142] | 1 | 1 | 1 | 1 | 1 | 1 | 1 | 0 | 0 | 1 | 1 | 1 | 10 |
| Townsend et al. [110] | 1 | 1 | 1 | 1 | 1 | 1 | 0 | 0 | 0 | 1 | 1 | 1 | 9 |
| Van Gelder & Bartz [158] | 1 | 1 | 0 | 1 | 1 | 1 | 1 | 0 | 0 | 1 | 1 | 1 | 9 |
| Vaquera et al. [171] | 1 | 1 | 1 | 1 | 1 | 1 | 1 | 0 | 0 | 1 | 1 | 1 | 10 |
| Xie et al. [94] | 1 | 1 | 1 | 1 | 1 | 1 | 1 | 0 | 0 | 1 | 1 | 1 | 10 |
|  | | | | | | | | | | | | | |

| **Supplementary Table 2**. Anthropometry variables according to playing position in adult male basketball players. | | | | | | |
| --- | --- | --- | --- | --- | --- | --- |
| **Study** | **Playing position** | **Competition level** | **Category** | **Height (cm)** | **Body Mass (kg)** | **Body fat %** |
| Alejandro et al. [184] | All | Spanish Liga ACB | Professional | 195.3 ± 2.7 | 98 ± 3.5 | 13.0 ± NR |
|  | All | Spanish LEB League | Professional | 198.3 ± 2.1 | 96.4 ± 2.4 | 10.5 ± NR |
|  | All | Spanish Liga EBA | Semi-professional | 193.5 ± 2.0 | 89.7 ± 2.9 | 10.9 ± NR |
|  | All | Spanish U20 National Team | Representative | 196.8 ± 1.9 | 93.4 ± 3.0 | 9.8 ± NR |
| Alemdaroğlu [61] | All | Turkish D1 | Professional | 194.8 ± 5.7 | 92.3 ± 9.8 | 10.1 ± 5.1 |
| Barrera-Domínguez et al. [64] | All | Spanish National Division | Professional | 187.6 ± 6.6 | 84.7 ± 11.9 | 11.4 ± 5.1 |
| Ben Abdelkrim et al. [65] | All | Tunisian National Team | Professional | 199.2 ± 7.3 | 91.4 ± 8.3 | 10.2 ± 2.4 |
| Bradic et al. [23] | All | Europe or Bosnia and Herzegovina | Professional | 201.5 ± 8.0 | 98.9 ± 9.9 | 12.5 ± 3.3 |
| Castagna et al. [155] | All | Regional Italian Club Competition | Amateur | 192.3 ± 8.9 | 84.4 ± 14.1 | 10.0 ± 1.2 |
| Chaouachi et al. [67] | All | Tunisian National Team | Professional | 195.6 ± 8.3 | 94.2 ± 10.2 | 14.0 ± 3.7 |
| Chatzinikolaou et al. [132] | All | Greek National Division | Professional | 196 ± 5.1 | 92.9 ± 7.8 | 9.8 ± 2.9 |
| de Sousa Fortes et al. [59] | All | State Basketball Championships Brazil | Professional | 197 ± 9 | 92.5 ± 9.2 | 19.7 ± 7.5 |
| Delextrat & Cohen [114] | All | BUSA D1 | Representative | 192.4 ± 9.4 | 90.6 ± 8.1 | 12.0 ± 5.0 |
|  | All | BUSA D2 | Collegiate | 187.2 ± 6.0 | 86.0 ± 11.9 | 12.5 ± 4.7 |
| Delextrat et al. [177] | All | English National Basketball League D2 | Semi-professional | 191.3 ± 5.8 | 88.0 ± 10.3 | 12.3 ± 4.6 |
| Dragonea et al. [133] | All | Greek A1 and A2 Leagues | Professional | 191.0 ± 2.0 | 92.9 ± 2.5 | 12.5 ± 1.1 |
| Fatouros et al. [125] | All | Greek Recreational | Amateur | 191.0 ± 0.1 | 87.9 ± 8.1 | 11.2 ± 2.1 |
| Ferioli et al. [16] | All | Italian Serie A | Professional | 198 ± 9 | 96.0 ± 11.1 | 11.2 ± 3.1 |
|  | All | Italian Serie A2 | Professional | 197 ± 8 | 92.7 ± 11.6 | 11.4 ± 3.6 |
|  | All | Italian Serie B | Semi-professional | 193 ± 8 | 90.5 ± 12.8 | 11.5 ± 3.9 |
|  | All | Italian Serie D | Amateur | 187 ± 8 | 80.0 ± 10.2 | 11.5 ± 4.3 |
| Ferioli et al. [54] | All | Italian Serie A and A2 | Professional | 197 ± 10 | 93.7 ± 13.0 | 10.9 ± 3.3 |
|  | All | Italian Serie B | Semi-professional | 188 ± 8 | 81.8 ± 10.3 | 10.5 ± 4.0 |
| Ferioli et al. [55] | All | Italian Serie A | Professional | 202 ± 9 | 99.3 ± 11.4 | 13.3 ± 4.1 |
|  | All | Italian Serie A2 | Professional | 198 ± 8 | 92.7 ± 12.7 | 10.5 ± 3.5 |
|  | All | Italian Serie B | Semi-professional | 193 ± 8 | 86.6 ± 11.7 | 10.8 ± 4.2 |
| Freitas et al. [71] | All | Spanish Liga EBA | Semi-professional | 194.5 ± 11.4 | 90.9 ± 14.8 | 12.3 ± 2.0 |
| Gerodimos et al. [186] | All | Greek National Team | Representative | 196.9 ± 10.4 | 99.5 ± 11.9 | 13.5 ± 3 |
| Gillam [119] | All | NCAA D2 | Collegiate | 189.2 ± 7.0 | 86 ± 8.7 | 13.5 ± 2.8 |
| Gomes et al. [72] | All | PSBC | Professional | 195.4 ± 11.3 | 101.5 ± 22.0 | 13.8 ± 3.6 |
| Gonzalez et al. [123] | All | NBA | Professional | 200.9 ± 9.4 | 104.7 ± 13.9 | 7.2 ± 1.9 |
| Gryko et al. [52] | All | Poland Professional League | Professional | 193.4 ± 8.1 | 90.2 ± 10.5 | 14.0 ± 3.1 |
| Heishman et al. [74] | All | NCAA D1 | Collegiate | 188.0 ± 7.9 | 100.8 ± 9.2 | 10.3 ± 2.2 |
| Heishman et al. [73] | All | NCAA D1 | Collegiate | 188.0 ± 7.9 | 100.8 ± 9.2 | 10.3 ± 2.2 |
| Kariyawasam et al. [56] | All | Sri Lanka National Level | Professional | 183.3 ± 8.4 | 79.3 ± 12.9 | 11.6 ± 5.5 |
| Köklü et al. [79] | All | Turkish D1 and D2 | Professional | 196.8 ± 7.7 | 96.5 ± 13.4 | 11.4 ± 4.4 |
|  | All | Turkish D1 | Professional | 197.9 ± 8.0 | 98.4 ± 12.3 | 10.9 ± 5.2 |
|  | All | Turkish D2 | Professional | 195.7 ± 7.40 | 94.7 ± 14.4 | 12.0 ± 3.5 |
| Korkmaz & Karahan [107] | All | Turkish D1 | Professional | 197 ± 7 | 95.3 ± 12.5 | 14.6 ± 3.7 |
|  | All | Turkish D2 | Professional | 194 ± 0.5 | 87.6 ± 7.3 | 12.5 ± 3.0 |
|  | All | Turkish D3 | Semi-professional | 195 ± 5 | 91.0 ± 8.6 | 12.1 ± 3.5 |
| Maggioni et al. [58] | All | Volunteer Players | Semi-professional | 182 ± 7 | 74 ± 10 | 8.9 ± 3.9 |
| Mandic et al. [82] | All | Serbian National League | Professional | 192.9 ± 6.0 | 86.2 ± 7.1 | 8.5 ± 4.2 |
| Masanovic et al. [187] | All | Serbian Premier League | Professional | 199.5 ± 7.4 | 99.6 ± 11.6 | 11.5 ± 2 |
|  | All | Serbian Fifth League | Amateur | 192.5 ± 4.6 | 90.6 ± 14.5 | 16.4 ± 6.8 |
| Mathur et al. [188] | All | Elite Nigerian Athletes | Representative | 182.5 ± 6.8 | 76.4 ± 4.9 | 7.6 ± 1.8 |
| Metaxas et al. [135] | All | Greek National League D1 | Professional | 193 ± 8 | 95.8 ± 11.5 | 11.0 ± 1.6 |
|  | All | Greek National League D2 | Professional | 191 ± 10 | 92.0 ± 15.1 | 11.9 ± 2.3 |
|  | All | Greek National League D3 | Semi-professional | 191 ± 6 | 91.4 ± 12.8 | 12.7 ± 2.0 |
|  | All | Greek National League D4 | Amateur | 190 ± 6 | 94.6 ± 11.5 | 14.3 ± 3.4 |
| Muratovic et al. [189] | All | Serbian Premier League | Professional | 199.5 ± 7.4 | 99.6 ± 11.6 | 11.5 ± 2 |
| Narazaki et al. [57] | All | NCAA D2 | Collegiate | 192.4 ± 11.7 | 91.9 ± 17.5 | 9.7 ± 5.9 |
| Nikolaidis et al. [109] | All | Italian First League | Professional | 195.7 ± 9.1 | 95.3 ± 11.7 | 18.8 ± 3.9 |
| Omorczyk et al. [122] | All | Polish Korona Sports Club | Amateur | 188.8 ± 5.8 | 84.4 ± 7.7 | 12.5 ± 1.7 |
| Ostojic et al. [84] | All | First National League Serbia | Professional | 199.5 ± 8.2 | 96.5 ± 11.2 | 11.5 ± 4.6 |
| Papanikolaou et al. [178] | All | Greek Club Level | Amateur | 186 ± 5 | 82.3 ± 6.6 | 19.8 ± 4.8 |
| Pehar et al. [48] | All | Bosnia and Herzegovina D1 | Professional | 197.4 ± 7.7 | 92.6 ± 10.8 | 7.8 ± 2.7 |
|  | All | Bosnia and Herzegovina D2 | Semi-professional | 191.6 ± 7.5 | 85.3 ± 9.8 | 10.5 ± 3.6 |
| Pehar et al. [85] | All | Bosnia and Herzegovina D1 | Professional | 194.6 ± 8.1 | 89.1 ± 10.8 | 8.9 ± 4.1 |
| Ponce-Gonzalez et al. [90] | All | Spanish Liga ACB | Professional | 196.4 ± 10.1 | 91.8 ± 10.6 | 10.3 ± 1.0 |
| Poole et al. [112] | All | Australian State Level | Semi-professional | 187.4 ± 7.6 | 86.2 ± 12.1 | 11.6 ± 2.1 |
| Popovic et al. [190] | All | Serbian Premier League | Professional | 199.5 ± 7.4 | 99.6 ± 11.6 | 11.5 ± 2 |
| Sallet et al. [126] | All | French League D1 and D2 | Professional | 196.4 ± 8.9 | 93.1 ± 13.2 | 12.6 ± 3.1 |
|  | All | French League D1 | Professional | 197.0 ± 8.5 | 93.9 ± 13.0 | 12.7 ± 2.7 |
|  | All | French League D2 | Professional | 195.7 ± 9.6 | 92.1 ± 13.6 | 12.4 ± 3.7 |
| Sanfilippo et al. [191] | All | NCAA D1 | Collegiate | 192.9 ± 8.9 | 92.1 ± 11.9 | 12.2 ± 2.0 |
| Scanlan et al. [137] | All | Australian State Level | Semi-professional | 189.6 ± 9.5 | 86.5 ± 18.7 | 14.7 ± 3.5 |
|  | All | Australian Recreational | Amateur | 185.9 ± 7.9 | 92.6 ± 8.4 | 23.8 ± 6.3 |
| Scanlan et al. [21] | All | Australian State Level | Semi-professional | 188.9 ± 7.9 | 97.4 ± 16.1 | 15.5 ± 5.0 |
| Scanlan et al. [60] | All | Australian State Level | Semi-professional | 188.9 ± 7.9 | 97.4 ± 16.1 | 15.5 ± 5.0 |
|  | Starters | Australian State Level | Semi-professional | 192.1 ± 7.7 | 100.5 ± 15.0 | 14.4 ± 5.5 |
|  | Non-starters | Australian State Level | Semi-professional | 185.7 ± 7.4 | 94.4 ± 17.9 | 16.3 ± 5.1 |
| Schiltz et al. [92] | All | European Cup D1 | Professional | 200.0 ± 2.9 | 94.9 ± 6.3 | 17.1 ± 2.9 |
| Sekulic et al. [49] | All | Bosnia and Herzegovina D1 and D2 | Semi-pro - Pro | 194.9 ± 8.1 | 89.3 ± 10.9 | 9 ± 3.4 |
|  | All | Bosnia and Herzegovina D1 | Professional | 197.4 ± 7.7 | 92.6 ± 10.8 | 7.8 ± 2.7 |
|  | All | Bosnia and Herzegovina D2 | Semi-professional | 191.6 ± 7.5 | 85.3 ± 9.8 | 10.5 ± 3.6 |
| Tomkinson et al. [145] | All | Australian NBL | Professional | 200 ± 8 | 96.9 ± 10.4 | 10.7 ± 2.4 |
|  | All | Australian State Level | Semi-professional | 189 ± 5 | 84.4 ± 10.9 | 12 ± 4 |
| Ahmed et al. [31] | All | Volunteer Players from Egypt | Amateur | 179.9 ± 1.7 | 67.6 ± 1.3 | NR |
| Annino et al. [62] | All | Italian National Federal League Level 2 | Professional | 194.7 ± 7.8 | 91 ± 8.17 | NR |
| Asadi [97] | All | Italian Provincial D1 | Amateur | 182.1 ± 9.2 | 78.5 ± 5.5 | NR |
| Aoki et al. [63] | All | State Basketball Championship Brazil | Professional | 199.1 ± 8.3 | 101.3 ± 12.1 | NR |
| Balsalobre-Fernandez et al. [100] | All | Spanish Liga ACB | Professional | 200 ± 10.4 | 98.4 ± 8.7 | NR |
| Balsalobre-Fernandez et al. [99] | All | Spanish Liga ACB | Professional | 200.2 ± 10.9 | 98.5 ± 8.6 | NR |
| Chen et al. [68] | All | Taiwan D1 College | Collegiate | 183.6 ± 9 | 82.4 ± 14.7 | NR |
| Ciacci & Bartolomei [96] | All | Italian National Level | Professional | 190.9 ± 7.3 | 83.8 ± 9.9 | NR |
|  | All | Italian National Level | Professional | 186.3 ± 4.9 | 79.7 ± 7.5 | NR |
| Dawes & Spiteri [51] | All | NCAA D2 | Collegiate | 196.3 ± 10.4 | 90.2 ± 9.7 | NR |
| de Araujo et al. [129] | All | Brazilian National Team and Elite National League | Professional | 195 ± 7 | 96.6 ± 14.8 | NR |
|  | Guard | Brazilian National Team and Elite National League | Professional | 187 ± 3 | 83.3 ± 5.2 | NR |
|  | Forward | Brazilian National Team and Elite National League | Professional | 197 ± 5 | 94.5 ± 4.8 | NR |
|  | Centre | Brazilian National Team and Elite National League | Professional | 201 ± 4 | 109.4 ± 19.1 | NR |
| Erkmen et al. [17] | All | Gazi University | Collegiate | 192.7 ± 7.6 | 88.1 ± 8.4 | NR |
| Ferioli et al. [136] | All | Italian Serie A and A2 | Professional | 198 ± 10 | 95.5 ± 13 | NR |
|  | All | Italian Serie B | Semi-professional | 190 ± 9 | 82.2 ± 11.6 | NR |
| Ferioli et al. [161] | All | Italian Serie A | Professional | 198.4 ± 9.5 | 95.9 ± 12.2 | NR |
|  | All | Italian Serie A2 | Professional | 197.4 ± 7.4 | 93.3 ± 11.4 | NR |
|  | All | Italian Serie B | Semi-professional | 192.8 ± 7.9 | 89 ± 11.6 | NR |
|  | All | Italian Serie D | Amateur | 187.1 ± 8.3 | 78 ± 9.8 | NR |
| Freitas et al. [70] | All | Spanish EBA D4 | Semi-professional | 197.7 ± 8.4 | 93.2 ± 14.5 | NR |
| Gerodimos et al. [142] | All | Greek Volunteer | Amateur | 195.1 ± 8.3 | 92.5 ± 9.4 | NR |
| Gocentas et al. [18] | PG, SG & SF | European Cups for Clubs | Professional | 191.5 ± 6.3 | 88.4 ± 8.2 | NR |
|  | PF & C | European Cups for Clubs | Professional | 206.1 ± 3.8 | 107 ± 7.51 | NR |
| Gocentas et al. [19] | All | Elite Lithuanian Competition | Professional | 197.6 ± 9.7 | 96.17 ± 12.31 | NR |
| Harbili [128] | All | Turkish National League D3 | Professional | 193.7 ± 7.5 | 92.7 ± 14.9 | NR |
| Hoffman et al. [103] | All | NCAA | Collegiate | 196.4 ± 11.9 | 89 ± 11.7 | NR |
| Ivanovic & Dopsaj [169] | All | Serbian First League | Professional | 200.1 ± 9.8 | 96.7 ± 12.7 | NR |
| Jallai et al. [77] | All | Estonian First League | Professional | 200.7 ± 7 | 97.4 ± 11.3 | NR |
| Jeffries et al. [118] | All | Australian State Level | Semi-professional | 184 ± 9 | 86 ± 11.9 | NR |
| Khlifa et al. [78] | All | Tunisian D1 | Professional | 192.6 ± 0.9 | 82.61 ± 0.8 | NR |
| Kipp et al. [106] | All | NCAA | Collegiate | 193.3 ± 10.2 | 80.5 ± 10.5 | NR |
| Dello Iacono et al. [69] | All | Professional Club U.K | Professional | 189.3 ± 3.2 | 88.2 ± 6.5 | NR |
| Laplaud et al. [80] | All | French Professional Championship | Professional | 198 ± 8 | 96 ± 10 | NR |
| Lehnert et al. [108] | All | Czech First League | Professional | 196.2 ± 9.6 | 92.9 ± 13.9 | NR |
| Lockie et al. [113] | All | Australian State Level | Semi-professional | 188 ± 10 | 88.5 ± 10.7 | NR |
|  | All | Australian Recreational | Amateur | 181 ± 8 | 83.5 ± 13.0 | NR |
| Lockie et al. [3] | All | NCAA D1 | Collegiate | 193 ± 8 | 93.2 ± 15.1 | NR |
|  | Backcourt | NCAA D1 | Collegiate | 188 ± 6 | 83.3 ± 8.1 | NR |
|  | Frontcourt | NCAA D1 | Collegiate | 201 ± 6 | 108.1 ± 9.9 | NR |
| Maffiuletti et al. [81] | All | French Basketball Federation D2 | Professional | 193.9 ± 6.9 | 87.7 ± 8.9 | NR |
| Mangine et al. [26] | All | NBA | Professional | 201.5 ± 9.4 | 102.6 ± 13 | NR |
| Markwick et al. [154] | All | Australian NBL | Professional | 196 ± 7 | 94.8 ± 8.2 | NR |
| McInnes et al. [134] | All | Australian NBL | Professional | 182.5 ± 6.8 | 76.4 ± 4.9 | NR |
| Miura et al. [83] | All | Japanese National Collegiate Tournament | Collegiate | 176.9 ± 7 | 68.9 ± 7.3 | NR |
| Montgomery et al. [28] | All | Australian State Level | Semi-professional | 184 ± 34 | 88.5 ± 14.7 | NR |
| Myles et al. [116] | All | U.S Volunteer | Amateur | 183 ± 10 | 81.9 ± 11.2 | NR |
| Pliauga et al. [86] | All | Lithuanian National Basketball League | Collegiate | 192.5 ± 5.4 | 83.5 ± 8.9 | NR |
| Pliauga et al. [39] | All | Lithuanian National Basketball League | Collegiate | 192.5 ± 5.4 | 83.5 ± 8.9 | NR |
| Popadic Gacesa et al. [127] | All | Elite Serbian Competition | Professional | 197.9 ± 6.7 | 94.3 ± 9.69 | NR |
| Puente et al. [14] | All | Spanish National Level Basketball Federation | Professional | 187.5 ± 8.5 | 83.8 ± 9.3 | NR |
| Ramirez-Campillo et al. [29] | All | South Chilean College System Basketball League | Collegiate | 184.4 ± 5.4 | 83.4 ± 7.1 | NR |
| Rodriguez-Rosell et al. [91] | All | Spanish D4 | Semi-professional | 188 ± 8 | 85.4 ± 11.1 | NR |
| Scanlan et al. [138] | All | Australian State Level | Semi-professional | 187.8 ± 7.7 | 88.8 ± 10.5 | NR |
| Scanlan et al. [30] | All | Australian State Level | Semi-professional | 190.5 ± 9.7 | 87.3 ± 12.4 | NR |
| Shalfawi et al. [93] | All | Professional Level Norway | Professional | 192 ± 8.2 | 89.8 ± 11.1 | NR |
| Spenst et al. [192] | All | University of Saskatchewan | Collegiate | 192.5 ± 8.7 | 89.8 ± 12.5 | NR |
| Stojanovic et al. [94] | All | Serbian Professional League | Professional | 197.1 ± 6.2 | 95.7 ± 8.8 | NR |
| Theoharopoulos et al. [170] | All | Greek A2 National League | Professional | 191.3 ± 11.2 | 87.9 ± 15.3 | NR |
| Townsend et al. [111] | All | NCAA D1 | Collegiate | 190 ± 12 | 87 ± 13.9 | NR |
| Vaquera et al. [179] | All | Spanish ACB and LEB | Professional | 195.8 ± 9.6 | 94.1 ± 11.4 | NR |
| Xie et al. [95] | All All | NCAA  NCAA | Collegiate Collegiate | 199 ± 7 190 ± 8 | 100.6 ± 7.7 85.5 ± 11.2 | NR NR |
| *Note*: Data are presented as mean ± standard deviation; Liga ACB = Liga Endesa Asociación de Clubs de Baloncesto; NR = data not reported in study; Spanish LEB = Liga Española de Baloncesto; Liga EBA = Liga Española de Baloncesto Aficionado; D1 = Division one competition; BUSA = British Universities Sports Association; D2 = Division two competition; NCAA = National Collegiate Athletic Association; PSBC = Paulista State Basketball Championships; NBA = National Basketball Association; D3 = Division three competition; D4 = Division four competition; PG = Point guard; SG = Shooting guard; SF = Small forward; PF = Power forward; C = Centre; Backcourt = Guards; Frontcourt = Forwards and Centres; NBL = Australian National Basketball League. | | | | | | |

| **Supplementary Table 3.** Summary of body composition tests and outcome variables across articles included in this review. | | |
| --- | --- | --- |
| **Test** | **Outcome measure** | **Studies** |
| Bioelectrical Impedance | BF% | Angyan et al. [43], Barrera-Domínguez et al. [64], Delextrat & Cohen [114], Mandic et al. [82], Pojskić et al. [87], Pojskić et al. [88], Pojskić et al. [89], Schiltz et al. [92] |
|  | Fat free mass (%) Lean mass (kg) | Pojskić et al. [87], Pojskić et al. [88], Pojskić et al. [89] Barrera-Domínguez et al. [64] |
| Electronic Scales | BF% | Alemdaroğlu [61], Köklü et al. [79], Omorczyk et al. [122], Soslu et al. [117] |
|  | Lean body mass (kg) | Omorczyk et al. [122] |
|  | Total body water (%) | Omorczyk et al. [122] |
| DEXA | BF% | Papanikolaou et al. [178], Sanfilippo et al. [191] |
|  | Bone mineral content | Sanfilippo et al. [191] |
|  | Lean body mass (kg) | Sanfilippo et al. [191] |
| Sum of 3 Skinfolds | Thickness (mm) | Omorczyk et al. [122] |
|  | BF% | Caterisano et al. [53], de Sousa Fortes et al. [59], Ferioli et al. [54], Ferioli et al. [16], Ferioli et al. [55], Gillam [119], Kariyawasam et al. [56], Maggioni et al. [58], Narazaki et al. [57], Scanlan et al. [4], Scanlan et al. [21], Scanlan et al. [60] |
| Sum of 4 Skinfolds | Thickness (mm) | Ben Abdelkrim et al. [65], Bolonchuk et al. [130] |
|  | BF% | Ben Abdelkrim et al. [65], Bolonchuk et al. [130], Bradic et al. [23], Buśko et al. [66], Chaouachi et al. [67], Cormery et al. [15], Metaxas et al. [135], Pehar et al. [48], Pehar et al. [85], Sallet et al. [126] |
| Sum of 5 Skinfolds | BF% | Gaurav et al. [185], Mathur et al. [188] |
|  | Thickness (mm) | Gaurav et al. [185] |
|  | Fat free mass (kg) | Gaurav et al. [185] |
| Sum of 6 Skinfolds | BF% | Balabinis et al. [98], Castagna et al. [155], Gonzalez et al. [123], Masanovic et al. [187], Muratovic et al. [189], Popovic et al. [190] |
| Sum of 7 Skinfolds | BF% | Chatzinikolaou et al. [132], Fatouros et al. [125], Gillam [119], Gomes et al. [72] , Gryko et al. [52], Ostojic et al. [84], Sekulic et al. [49] |
|  | Thickness (mm) | Montgomery et al. [28], Parr et al. [121] |
|  | Lean body mass (kg) | Gillam [119], Gomes et al. [72] |
| Sum of 8 Skinfolds | BF% | Freitas et al. [71] |
|  | Thickness (mm) | Freitas et al. [71], Hoffman et al. [103] |
| Sum of 9 Skinfolds | Thickness (mm) | Spenst et al. [192], Tomkinson et al. [145] |
|  | BF% | Scanlan et al [137], Tomkinson et al. [145] |
|  | Muscle mass % and kg | Spenst et al. [192] |
| Sum of 10 Skinfolds | BF% | Boone & Bourgois [27], Boone et al. [131], Nikolaidis et al. [109] |
| Hydrostatic Weighing | BF% | Hunter & Hilyer [120], Hunter et al. [105], Parr et al. [121] |
| O-Scale System | BF% | Ponce-González et al. [90] |
| Air displacement plethysmography | BF% Fat mass (kg) Fat free mass (kg)  Fat mass index Fat free mass index | Fields et al. [50], Heishman et al. [74], Heishman et al. [73] Fields et al. [50] Fields et al. [50] Fields et al. [50] Fields et al. [50] |
| *Note*: BF% = body fat percentage; DEXA = dual energy x-ray absorptiometry. | | |

| **Supplementary Table 4.** Summary of jump tests and outcome variables across articles included in this review. | | |
| --- | --- | --- |
| **Test** | **Outcome variable** | **Studies** |
| Countermovement Jump | Height (cm) | Alemdaroglu [61], Annino et al. [62], Aoki et al. [63], Barrera-Domínguez et al. [64], Ben Abdelkrim et al. [65], Boone & Bourgois [27], Buśko et al. [66], Chaouachi et al. [67], Chen et al. [68], Ciacci & Bartolomei [96], de Sousa Fortes et al. [59], Dello Iacono et al. [69], Ferioli et al. [16], Ferioli et al. [54], Ferioli et al. [55], Freitas et al. [70], Freitas et al. [71], Gerodimos et al. [142], Gomes et al. [72], Heishman et al. [74], Heishman et al. [73], Jallai et al. [77], Khlifa et al. [78], Köklü et al. [79], Laplaud et al. [80], Maffiuletti et al. [81], Maggioni et al. [58], Mandic et al. [82], Markwick et al. [154], Mikolajec et al. [160], Mitić et al. [115], Miura et al. [83], Ostojic et al. [84], Pehar et al. [48], Pehar et al. [85], Pliauga et al. [86], Pojskić et al. [87], Pojskić et al. [88], Pojskić et al. [89], Ponce-González et al. [90], Puente et al. [14], Rodriguez-Rosell et al. [91], Schiltz et al. [92], Shalfawi et al. [93], Soslu et al. [117], Stojanovic et al. [94], Xie et al. [95] |
|  | Peak power (W) | Ben Abdelkrim et al. [65], Boone & Bourgois [27], Buśko et al. [66], Ferioli et al. [16], Ferioli et al. [54], Ferioli et al. [55], Freitas et al. [70], Freitas et al. [71], Heishman et al. [74], Heishman et al. [73], Pojskić et al. [88], Ponce-González et al. [90], Shalfawi et al. [93], Soslu et al. [117] |
|  | Relative power (w/kg^-0.67^) Relative peak power (W/kg^-1^) | Ben Abdelkrim et al. [65] Ferioli et al. [16], Ferioli et al. [54], Ferioli et al. [55] |
|  | Peak force (N) Relative peak force (N/kg^-1^) | Ferioli et al. [16], Ferioli et al. [54], Ferioli et al. [55] Ferioli et al. [16], Ferioli et al. [54], Ferioli et al. [55] |
|  | Rate of force development (N⋅s^-1^) | Ponce-González et al. [90] |
|  | Initial velocity (m/sec) | Ponce-González et al. [90], Mikolajec et al. [160] |
|  | Mean power (W) Positive mechanical impulse (kgf⋅s^-1^) Maximal instantaneous power (W) Maximal instantaneous vertical velocity (m/s) Index of elasticity (%) Eccentric utilisation ratio | Mangine et al. [26], Ponce-González et al. [90] Ponce-González et al. [90] Ponce-González et al. [90] Ponce-González et al. [90] Ponce-González et al. [90] Xie et al. [95] |
| Hexagonal Bar CMJ | Vertical force-velocity profile Theoretical maximal unloaded velocity (V0) Theoretical maximal force (F0) | Barrera-Domínguez et al. [64] Barrera-Domínguez et al. [64] Barrera-Domínguez et al. [64] |
| Unilateral CMJ | Height (cm) Peak power (W) Mean power (W) | Kariyawasam et al. [56], Mitić et al. [115] Mangine et al. [26]  Mangine et al. [26] |
| Vertical Jump / CMJ (arms) | Height (cm) | Ahmed [31], Angyan et al. [43], Annino et al. [62], Balabinis et al. [98], Balsalobre-Fernandez et al. [99], Balsalobre-Fernandez et al. [100], Buśko et al. [66], Ciacci & Bartolomei [96], Dawes & Spiteri [51], de Sousa Fortes et al. [59], Gerodimos et al. [142], Hoffman et al. [102], Hoffman et al. [103], Hoffman et al. [104], Hunter et al. [105], Kariyawasam et al. [56], Kipp et al. [106], Korkmaz & Karahan [107], Lehnert et al. [108], Lockie et al. [3], Mandic et al. [82], Montgomery et al. [28], Myles et al. [116], Nikolaidis et al. [109], Pliauga et al. [39], Ponce-González et al. [90], Puente et al. [14], Rauch et al. [110], Rodriguez-Rosell et al. [91], Schiltz et al. [92], Townsend et al. [111] |
|  | Peak power (W) | Buśko et al. [66], Korkmaz & Karahan [107], Ponce-González et al. [90] |
|  | Rate of force development (N⋅s^-1^) | Ponce-González et al. [90] |
|  | Initial velocity (m/sec) | Ponce-González et al. [90] |
|  | Mean power (W) Positive mechanical impulse (kgf⋅s^-1^) Maximum instantaneous power (W) Maximum instantaneous vertical velocity (m/s) | Ponce-González et al. [90] Ponce-González et al. [90] Ponce-González et al. [90] Ponce-González et al. [90] |
|  | Power (kg^.^m/sec) Vertical jump loss (%) Use of arm swing (IUA) Mean absolute vertical jump power (W) Mean relative vertical jump power (W/kg) Plantar flexion velocity (m⋅s^-1^) Knee extension velocity (m⋅s^-1^)  Hip extension velocity (m⋅s^-1^) Relative concentric force (Fz⋅kg^-1^) Relative breaking force (Fz⋅kg^-1^) Net relative impulse (N⋅s⋅kg^-1^) Total movement time (s) | Gillam [119] Balsalobre-Fernandez et al. [99] Ponce-González et al. [90] Korkmaz & Karahan [107] Korkmaz & Karahan [107] Rauch et al. [110] Rauch et al. [110] Rauch et al. [110] Rauch et al. [110] Rauch et al. [110] Rauch et al. [110] Rauch et al. [110] |
| Vertical Jump (1 or 2 steps) | Height (cm) | Ciacci & Bartolomei [96], Delextrat & Cohen [114], Hunter & Hilyer [120], Pehar et al. [48] |
| Running Vertical Jump | Height (cm) | Lockie et al. [3], Miura et al. [83], Pehar et al. [48], Rodriguez-Rosell et al. [91] |
| Squat Jump | Height (cm) | Alemdaroglu [61], Aoki et al. [63], Boone & Bourgois [27], Chaouachi et al. [67], Ciacci & Bartolomei [96], Gerodimos et al. [142], Gomes et al. [72], Ilie & Cristian, [168], Jallai et al. [77], Khlifa et al. [78], Köklü et al. [79], Maffiuletti et al. [81], Maggioni et al. [58], Mitić et al. [115], Pojskić et al. [89], Ponce-González et al. [90], Rodriguez-Rosell et al. [91], Shalfawi et al. [93], Soslu et al. [117], Xie et al. [95] |
|  | Peak power (W) | Boone & Bourgois [27], Ponce-González et al. [90], Shalfawi et al. [93], Soslu et al. [117] |
|  | Rate of force development (N⋅s^-1^) | Ponce-González et al. [90] |
|  | Initial velocity (m/sec) | Ponce-González et al. [90] |
|  | Mean power (W)  Positive mechanical impulse (kgf⋅s^-1^) Maximum instantaneous power (W) Maximum instantaneous vertical velocity (m/s) Flight time (ms) Eccentric Utilisation Ratio | Ponce-González et al. [90] Ponce-González et al. [90] Ponce-González et al. [90] Ponce-González et al. [90] Ilie & Cristian [168] Xie et al. [95] |
| Repeated Jump Tests | Height (cm)  Mean power (W) Mean height (cm) Take off velocity Peak force (N) Flight time (ms) Contact time (ms) Mean contact time (ms) Reactive strength index Power of jumps RB index | Fatouros et al. [125], Ilie & Cristian [168], Miura et al. [83], Petway et al. [44], Schiltz et al. [92], Xie et al. [95]  Fatouros et al. [125], Gonzalez et al. [123] Ciacci & Bartolomei [96] Gonzalez et al. [123] Petway et al. [44] Castagna et al. [155], Ciacci & Bartolomei [96], Ilie & Cristian [168] Castagna et al. [155], Ilie & Cristian [168], Petway et al. [44] Ciacci & Bartolomei [96], Castagna et al. [155], Ciacci & Bartolomei [96], Petway et al. [44] Ilie & Cristian [168] Ilie & Cristian [168] Ciacci & Bartolomei [96] |
| Unilateral Repeated Jump Test | Height (cm) Contact time (ms) Jump height / Contact time | Miura et al. [83] Miura et al. [83] Miura et al. [83] |
| Spike Jump | Height (cm) Peak power (W) | Buśko et al. [66]  Buśko et al. [66] |
| Drop Jump | Height (cm) | Jallai et al. [77], Mitić et al. [115], Pehar et al. [48], Schiltz et al. [92] |
|  | Reactive strength index | Markwick et al. [154] |
|  | Contact time (ms) | Markwick et al. [154] |
|  | Flight time (ms) | Markwick et al. [154] |
| Unilateral Drop Jump | Height (cm) Reactive strength index | Barrera-Domínguez et al. [64], Schiltz et al. [92] Barrera-Domínguez et al. [64] |
| Depth Jump | Reactive strength index | Pehar et al. [48] |
| 18-inch Repetitive Bench Jump | Jumps in 30-seconds (*n*) | Hunter & Hilyer [120] |
| Visual Reaction Time to Jump | Time (s) | Pehar et al. [85] |
| Triple Hop Test (unilateral) | Distance (m) | Barrera-Domínguez et al. [64] |
| Broad Jump | Horizontal displacement (m) | Freitas et al. [70], Freitas et al. [71], Omorczyk et al. [122], Pehar et al. [48], Pehar et al. [85], Xie et al. [95] |
| 5-Continuous Broad Jumps | Horizontal displacement (m) | Ben Abdelkrim et al. [65], Chaouachi et al. [67], Khlifa et al. [78] |
| *Note*: RSI-Mod = reactive strength index modified; FT = flight time method of calculation; Imp-Mom = Impulse-momentum method of calculation); RPD = rate of power development; CMJ = Countermovement jump without arm swing; CMJ (arms) = countermovement jump with arm swing, otherwise known as the Sergeant Jump; IUA = index of use of arm swing; RB index = best height of stiffness test / best height CMJ*100. | | |

| **Supplementary Table 5.** Summary of linear sprint tests and outcome variables across articles included in this review. | | |
| --- | --- | --- |
| **Test** | **Outcome variable** | **Studies** |
| 5-m Sprint | Time (s)  Speed (km/h) Acceleration (m/s^-2^) | Barrera-Domínguez et al. [64], Ben Abdelkrim et al. [65], Boone & Bourgois [27], Chaouachi et al. [67], Dawes & Spiteri [51], Maggioni et al. [58], Mikolajec et al. [160], Poole et al. [112], Scanlan et al. [4], Scanlan et al. [21] Barrera-Domínguez et al. [64], Poole et al. [112]  Poole et al. [112] |
| 5-10-m Sprint | Time (s)  Speed (km/h)  Acceleration (m/s^-2^) | Poole et al. [112]  Poole et al. [112]  Poole et al. [112] |
| 10-m Sprint | Time (s)   Speed (km/h) Acceleration (m/s^-2^) Theoretical maximal velocity (V0) Horizontal force (F0) Horizontal power (Pmax) Force velocity profile Velocity (m/s) | Alemdaroglu [61], Barrera-Domínguez et al. [64], Ben Abdelkrim et al. [65], Boone & Bourgois [27], Chaouachi et al. [67], Freitas et al. [71], Haugen et al. [159], Köklü et al. [79], Lockie et al. [113], Maggioni et al. [58], Pliauga et al. [86], Poole et al. [112], Rodriguez-Rosell et al. [91], Scanlan et al. [4], Scanlan et al. [21], Shalfawi et al. [93], Soslu et al. [117] Barrera-Domínguez et al. [64], Poole et al. [112]  Poole et al. [112] Haugen et al. [159] Haugen et al. [159] Haugen et al. [159] Haugen et al. [159] Xie et al. [95] |
|  | Rate of force (N⋅s^-1^) | Barrera-Domínguez et al. [64] |
| 20-m Sprint | Time (s)    Time (1080 sprint)  Velocity (m/s) Stride frequency (Hz) Stride length (m) | Angyan et al. [43], Dawes & Spiteri [51], de Sousa Fortes et al. [59], Delextrat & Cohen [114], Gomes et al. [72], Korkmaz & Karahan [107], Maggioni et al. [58], Mikolajec et al. [160], Montgomery et al. [28], Pliauga et al. [39], Pojskić et al. [87], Pojskić et al. [89], Ramirez-Campillo et al. [29], Rodriguez-Rosell et al. [91], Scanlan et al. [4], Scanlan et al. [21], Scanlan et al. [30], Shalfawi et al. [93] Townsend et al. [111] Fujii et al. [158], Mikolajec et al. [160] Fujii et al. [158]  Fujii et al. [158] |
| 10-20-m Sprint | Velocity (m/s) Peak speed (m/s) | Xie et al. [95] Scanlan et al. [21], Scanlan et al. [4] |
| 27-m Sprint | Time (s) | Hoffman et al. [102], Hoffman et al. [103], Hoffman et al. [104] |
| 30-m Sprint | Time (s) | Alemdaroglu [61], Barrera-Domínguez et al. [64], Ben Abdelkrim et al. [65], Chaouachi et al. [67], Köklü et al. [79], Soslu et al. [117] |
|  | Force velocity profile | Barrera-Domínguez et al. [64] |
| 35-m Sprint | Time (s) Sprint power (W) | Balsalobre-Fernandez et al. [100] Balsalobre-Fernandez et al. [100] |
| 40-m Sprint | Time (s) | Haugen et al. [159], Kariyawasam et al. [56], Shalfawi et al. [93] |
|  | Theoretical maximal velocity (V0) Horizontal force (F0) Horizontal power (Pmax) Force velocity profile | Haugen et al. [159] Haugen et al. [159] Haugen et al. [159] Haugen et al. [159] |
| ¾ Basketball Court Sprint | Time (s) | Lockie et al. [3] |
| 15-yard Sprint | Time (s) | Gillam [119] |
| 50-yard Sprint | Time (s) | Gillam [119] |
| *Note*: 1080 = Time recorded using 1080 sprint which applies 1-kg resistance | | |

| **Supplementary Table 6.** Summary of change-of-direction tests with outcome variables reported in articles included in this review. | | | |
| --- | --- | --- | --- |
| **Test** | **Outcome variable** | **Studies** | |
| Agility T-Test | Time (s) | Alemdaroglu [61], Asadi et al. [97], Ben Abdelkrim et al. [65], Chaouachi et al. [67], Delextrat & Cohen [114], Freitas et al. [70], Freitas et al. [71], Gomes et al. [72], Hoffman et al. [102], Hoffman et al. [103], Hoffman et al. [104], Köklü et al. [79], Maggioni et al. [58], Mitić et al. [115], Myles et al. [116], Pojskić et al. [89], Pojskić et al. [87], Poole et al. [112], Sekulic et al. [49], Soslu et al. [117] | |
| Modified Agility T-Test | Time (s) | Barrera-Domínguez et al. [64] | |
| Right Boomerang Run | Time (s) | Gillam [119] | |
| 20m (10+10m) Shuttle Test | Time (s) | Maggioni et al. [58] |  |
| Lateral Shuffle 0-5m | Time (s) left and right foot start | Poole et al. [112] | |
| Lateral Shuffle 5-10m | Time (s) left and right foot start | Poole et al. [112] | |
| Lateral Shuffle 0-10m | Time (s) left and right foot start | Poole et al. [112] | |
| 5+5 Change of Direction Test | 45° Time (s) 90° Time (s)  180° Time (s) | Barrera-Domínguez et al. [64] Barrera-Domínguez et al. [64] Barrera-Domínguez et al. [64] | |
| 5-10-5 Pro Agility Test | Time (s) | Kariyawasam et al. [56], Townsend et al. [111] | |
| Hexagon Agility Test | Time (s) | Mitić et al. [115] | |
| Illinois Agility Test | Time (s) | Asadi et al. [97], Mitić et al. [115] | |
| Lane Agility Test | Time (s) | Dawes & Spiteri [51], Lockie et al. [3], Mitić et al. [115], Townsend et al. [111] | |
| COD Speed Test | Time (s) | Scanlan et al. [21], Scanlan et al. [4], Scanlan et al. [60] | |
| Y-Shaped Agility Test | Time (s) Time (s) dominant and non-dominant leg start | Jeffries et al. [118], Lockie et al. [113], Pehar et al. [85]  Sekulic et al. [49] | |
| Basketball Specific Agility Test | Time (s) | Montgomery et al. [28] | |
| Ulatowski Defence Test | Time (s) | Omorczyk et al. [122] | |
| Multi-stage Changes-of-Direction Exercise Test | Instantaneous metabolic power (w⋅kg^-1^) Instantaneous running speed (m⋅s^-1^) Peak torque (Nm) Fatigue index (%) | Ferioli et al. [161]  Ferioli et al. [161]  Ferioli et al. [161]  Ferioli et al. [161] | |
| COD Course | Time (s) | Ramirez-Campillo et al. [29], Scanlan et al. [30] | |
| 5-0-5 Test | Time (s) | Van Gelder & Bartz [162] | |
| *Note*: COD = Change-of-direction; COD Course = Change-of-direction course; Y-shaped agility test = pre-planned change of direction. | | | |
|  | | | |

| **Supplementary Table 7.** Summary of agility tests with outcome variables reported in articles included in this review. | | |
| --- | --- | --- |
| **Test** | **Outcome variable** | **Studies** |
| Reactive Agility Test | Time (s) Response time (ms) Decision making time (ms) | Scanlan et al. [21], Scanlan et al. [4], Scanlan et al. [60] Scanlan et al. [21], Scanlan et al. [4], Scanlan et al. [60] Scanlan et al. [21], Scanlan et al. [4], Scanlan et al. [60] |
| Reactive Change-of-Direction Test | Time (s) left and right leg start | Jeffries et al. [118], Lockie et al. [113] |
| Reactive Y-Change-of-Direction Test | Time (s) Time (s) dominant and non-dominant leg start | Pehar et al. [85] Sekulic et al. [49] |
|  | | |

| **Supplementary Table 8.** Summary of strength tests and outcome variables reported in articles included in this review. | | |
| --- | --- | --- |
| **Test** | **Outcome variable** | **Studies** |
| Bench Press 1RM | 1RM (kg) | Balabinis [98], Ben Abdelkrim et al. [65], Caterisano et al. [53], Chaouachi et al. [67], Delextrat & Cohen [114], Gillam [119], Gomes et al. [72], Hoffman et al. [102], Hoffman et al. [103], Hoffman et al. [104], Hunter & Hilyer [120], Hunter et al. [105], Kariyawasam et al. [56] |
|  | Relative 1RM | Ben Abdelkrim et al. [65] |
| Bench Press 3RM | 3RM (kg) Calculated 1RM (kg) | Dawes & Spiteri [51]  Dawes & Spiteri [51] |
| Bench Press 4-6RM | Estimated 1RM (kg) MPV (m/s) | Freitas et al. [71]  Freitas et al. [71] |
| Bench Press 6RM | Estimated 1RM (kg) Peak power (W) | Freitas et al. [70]  Freitas et al. [70] |
| Bench Press Loads 30-75%1RM | Power-load profile | Freitas et al. [71] |
| Bench Press at Body Weight | Repetitions (*n*) | Omorczyk et al. [122] |
| Bench Press Incremental Load 47.5-57.5-67.5-77.5kg | Propulsive peak power (W) Propulsive peak force (N) Absolute propulsive peak power load 1RM (kg) | Balsalobre-Fernandez et al. [99]  Balsalobre-Fernandez et al. [99]  Balsalobre-Fernandez et al. [99] Balsalobre-Fernandez et al. [99] |
| Bench Press 185 lb | Repetitions (*n*) | Lockie et al. [3] |
| Hydraulic Isokinetic Bench Press | 1RM (kg) Power (lb/sec) Repetitions to 50% maximum power (*n*) | Parr et al. [121] Parr et al. [121] Parr et al. [121] |
| Front Squat 1RM | 1RM (kg) | Rodriguez-Rosell et al. [91], Townsend et al. [111] |
| Back Squat 1RM | 1RM (kg) | Ben Abdelkrim et al. [65], Cabarkapa et al. [124], de Sousa Fortes et al. [59], Gillam [119], Hoffman et al. [103], Hoffman et al. [102], Hoffman et al. [104] |
|  | Relative 1RM | Ben Abdelkrim et al. [65] |
| Back Squat 3RM | 3RM (kg) Estimated 1RM (kg) | Dawes & Spiteri [51]  Dawes & Spiteri [51] |
| ½ ROM Back Squat | 1RM (kg) | Balabinis et al. [98], Chaouachi et al. [67], Freitas et al. [70], Freitas et al. [71] |
|  | MPV (m/s) | Freitas et al. [71] |
| Back Squat at 80%1RM | Mean power (W) | Gonzalez et al. [123] |
| Back Squat at 30-75%1RM Back Squat 30-80%1RM | Power-load profile Mean power (W) Power-load curve | Freitas et al. [71] Cabarkapa et al. [124] Cabarkapa et al. [124] |
| Back Squat at Body Weight | Repetitions (*n*) | Omorczyk et al. [122] |
| Hang Clean 1RM | 1RM (kg) | Townsend et al. [111] |
| Isometric Mid-Thigh Pull | Peak force (N) Rate of force development (N⋅s^-1^) | Townsend et al. [111]  Townsend et al. [111] |
| Standing Press 1RM | 1RM (kg) | Parr et al. [121] |
| Maximum Push Ups | Repetitions (*n*) | Gillam [119] |
| Maximum Push Ups 60 s | Repetitions (*n*) | Pojskić et al. [87], Pojskić et al. [89] |
| Maximum Sit Ups 30 s | Repetitions (*n*) | Omorczyk et al. [122] |
| Maximum Sit Ups 60 s | Repetitions (*n*) | Ahmed [31], Hunter & Hilyer [120], Kariyawasam et al. [56], Pojskić et al. [87], Pojskić et al. [89] |
| Maximum Squat Thrusts | Repetitions (*n*) | Gillam [119] |
| Lateral Pull Down 1RM | 1RM (kg) | Balabinis et al. [98] |
| 30-75%1RM Hip Thrust | Power-load profile | Freitas et al. [71] |
| Hip Thrust 1RM | 1RM (kg) MPV (m/s) | Freitas et al. [71]  Freitas et al. [71] |
| Leg Press 1RM | 1RM (kg) | Balabinis et al. [98], Caterisano et al. [53], Kariyawasam et al. [56] |
| Bent Arm Hang | Time (s) | Angyan et al. [43], Omorczyk et al. [122] |
| Core Endurance Test Series | Time (s) | Chen et al. [68] |
| Grip Strength Dynamometer | Maximum force (kg) | Ahmed [31], Angyan et al. [43], Kariyawasam et al. [56], Omorczyk et al. [122] |
| Isokinetic Upper-limb Strength | Flexion (Nm) Extension (Nm) | Buśko [24]  Buśko [24] |
| Isokinetic Leg Strength | Flexion torque (Nm)  Extension torque (Nm) | Alemdaroglu [61], Bradic et al. [23], Buśko [24], Ferioli et al. [161], Hadzic et al. [25], Harbili [128], Köklü et al. [79], Metaxas et al. [135], Schiltz et al. [92], Soslu et al. [117], Theoharopoulos et al. [170]  Alemdaroglu [61], Bradic et al. [23], Boone & Bourgois [27], Buśko [24], Delextrat & Cohen [114], Hadzic et al. [25], Köklü et al. [79], Maffiuletti et al. [81], Metaxas et al. [135], Schiltz et al. [92], Soslu et al. [117], Theoharopoulos et al. [170] |
| Isokinetic Ankle Strength | Plantar flexion torque (Nm) Dorsiflexion torque (Nm) | Bradic et al. [23], Buśko [24]  Bradic et al. [23], Buśko [24] |
| Seated Basketball Throw | Throw speed (km/hr) | Chen et al. [68] |
| Seated Medicine Ball Throw | Distance (m) | Kariyawasam et al. [56], Pojskić et al. [87], Pojskić et al. [89] |
| *Note*: 1RM = one repetition maximum; 3RM = three repetition maximum; 4-6RM = four to six repetition maximum; MPV = Mean propulsive velocity; ROM = range of motion. | | |

| **Supplementary Table 9.** Summary of anaerobic capacity tests and outcome variables in article included in this review. | | |
| --- | --- | --- |
| **Test** | **Outcome variable** | **Studies** |
| *Running tests* |  |  |
| 10 x 5-m Shuttle Sprint Test | Time (s) | Omorczyk et al. [122] |
| 4 x 9-m Shuttle Sprint Test | Time (s) | Asadi et al. [97] |
| 5 x 10-m Shuttle Sprint Test | Time (s) | Boone & Bourgois [27] |
| 10 x 15-m Shuttle Sprint Test | Total time (s) Best time (s) Decrement (%) | Freitas et al. [70], Stojanovic et al. [94]  Freitas et al. [70], Stojanovic et al. [94]  Freitas et al. [70], Stojanovic et al. [94] |
| 12 x 20-m Repeat Sprint Test | Best time (s) Mean time (s) | Aoki et al. [63]  Aoki et al. [63] |
| 10 x 28-m Repeat Sprint Test | Time (s) | Scanlan et al. [138] |
| Full Court Shuttle Run | Time (s) Fatigue index ^3^ (%) | Dawes & Spiteri [51], Fatouros et al. [125], Maggioni et al. [58], Montgomery et al. [28], Myles et al. [116] Fatouros et al. [125] |
| 6 x 4-s Repeat Sprint Test | Time (s) Horizontal force (N) Treadmill speed (km/h) Vertical force (N) Peak power (W) Mean power (W) Fatigue index (%) Stride kinematics (see note) | Delextrat et al. [177] Delextrat et al. [177] Delextrat et al. [177] Delextrat et al. [177] Delextrat et al. [177] Delextrat et al. [177] Delextrat et al. [177] Delextrat et al. [177] |
| 10 x 10-s Repeat Sprint Test | Blood lactate concentration (mmol/L) Blood bicarbonate concentration (mmol/L) Blood hydrogen ion concentration (mmol/L) | Ferioli et al. [136], Ferioli et al. [16], Ferioli et al. [55]  Ferioli et al. [136], Ferioli et al. [16], Ferioli et al. [55]  Ferioli et al. [136], Ferioli et al. [16], Ferioli et al. [55] |
| RAST | Peak power (W)  Relative maximum power (W/kg) Mean power (W)  Relative mean power (W/kg) Relative minimum power (W) Minimum power (W) Fatigue index (%)  Relative fatigue index (%) | Balsalobre-Fernandez et al. [99], Balsalobre-Fernandez et al. [100], de Araujo et al. [129], Pojskić et al. [87], Pojskić et al. [88], Pojskić et al. [89],  de Araujo et al. [129], Pojskić et al. [87], Pojskić et al. [88], Pojskić et al. [89]  Balsalobre-Fernandez et al. [99], Balsalobre-Fernandez et al. [100], de Araujo et al. [129], Pojskić et al. [87], Pojskić et al. [88], Pojskić et al. [89] de Araujo et al. [129], Pojskić et al. [88], Pojskić et al. [89]  de Araujo et al. [129], Pojskić et al. [88]  de Araujo et al. [129], Pojskić et al. [87], Pojskić et al. [88], Pojskić et al. [89] Balsalobre-Fernandez et al. [99], Balsalobre-Fernandez et al. [100], de Araujo et al. [129], Pojskić et al. [87], Pojskić et al. [88], Pojskić et al. [89] de Araujo et al. [129], Pojskić et al. [87], Pojskić et al. [89] |
| Incremental 20m Sprint Test | Peak blood lactate concentration (mmol/L) | de Araujo et al. [129] |
| *Cycle tests* |  |  |
| Wingate Anaerobic Test | Peak power (W) | Alemdaroğlu [61], Balabinis et al. [98], Harbili [128], Nikolaidis et al. [109], Popadic Gacesa et al. [127], Sallet et al. [126], Soslu et al. [117] |
|  | Mean power (W) | Alemdaroğlu [61], Delextrat & Cohen [114], Harbili [128], Fatouros et al. [125], Nikolaidis et al. [109], Popadic Gacesa et al. [127], Soslu et al. [117] |
|  | Fatigue index (%) Time to peak power (ms) Blood lactate concentration (mmol/L) | Alemdaroğlu [61], Delextrat & Cohen [114], Harbili [128], Nikolaidis et al. [109], Sallet et al. [126] Popadic Gacesa et al. [127] Fatouros et al. [125] |
| PWC170 Cycle Test | Heart rate (bpm) Watts at heart rate of 170 bpm (W/kg)  Watts per kg at heart rate of 170 bpm (W/kg) | Nikolaidis et al. [109] Nikolaidis et al. [109] Nikolaidis et al. [109] |
| 4 x 7-s Resisted Cycle Sprints | Peak power (W) Theoretical maximal velocity (V0)  Theoretical maximal force (N0) | Nikolaidis et al. [109] Nikolaidis et al. [109] Nikolaidis et al. [109] |
| 2 x 10-s Resisted Cycle Sprint 15-s and 60-s Resisted Cycle Sprints | Peak power (W) Peak power (W) Blood lactate concentration (mmol/L) PaCO_2_ (mmHg) Minimum blood pH | Buśko et al. [66] Lysenko [180] Lysenko [180] Lysenko [180] Lysenko [180] |
| *Additional tests* | | |
| Power Step Test | Peak power (W) | Tavino et al. [181] |
| Wingate Arm Crank Test | Peak power (W) Mean power (W) Fatigue index (%) | Chen et al. [68] Chen et al. [68] Chen et al. [68] |
| *Note*: RAST = Running-based Anaerobic Sprint Test; AT = anaerobic threshold; VE = Pulmonary ventilation; HR = Heart rate; Fatigue index ^3^ = fatigue index calculated over 3 trials each separated by 2 min of passive rest; Stride kinematics measured include contact time (ms), flight time (ms), stride duration (ms), swing time (ms), stride frequency (Hz), and stride length (m); PWC170 = Physical work capacity at heart rate of 170 bpm; V0 = theoretical maximal velocity; F0 = theoretical maximal force; PaCO_2_ = partial pressure of carbon dioxide. | | |

| **Supplementary Table 10.** Summary of aerobic capacity tests and outcome variables reported in articles included in this review. | | |
| --- | --- | --- |
| **Test** | **Outcome variable** | **Studies** |
| Yo-Yo Intermittent Recovery Test Level 1 | Distance (m) | Aoki et al. [63], Ben Abdelkrim et al. [65], Chaouachi et al. [67], Ferioli et al. [136], Ferioli et al. [16], Ferioli et al. [55], Gomes et al. [72], Maggioni et al. [58], Montgomery et al. [28], Scanlan et al. [137], Scanlan et al. [138] |
|  | Estimated VO_2max_ (ml/kg/min)  Peak speed (km/h) | Ben Abdelkrim et al. [65], Chaouachi et al. [67], Gomes et al. [72], Scanlan et al. [21], Scanlan et al. [60], Scanlan et al. [138] Ben Abdelkrim et al. [65] |
| Yo-Yo Endurance Test Level 1 | Estimated VO_2max_ (ml/kg/min) | Castagna et al. [155] |
|  | Distance (m) | Castagna et al. [155] |
| Yo-Yo Endurance Test Level 2 | Distance (m) Mean speed (km/h)  Maximum speed (km/h) Accelerations ^5^ (*n*) Decelerations ^5^ (*n*) Body load (arb. unit) | Papanikolaou et al. [178] Papanikolaou et al. [178] Papanikolaou et al. [178] Papanikolaou et al. [178] Papanikolaou et al. [178] Papanikolaou et al. [178] |
| Multi-Stage Fitness Test | Estimated VO_2max_ (ml/kg/min) | Alemdaroğlu [61], Dawes & Spiteri [51], Kariyawasam et al. [56], Köklü et al. [79], Korkmaz & Karahan [107], Ostojic et al. [84], Pojskić et al. [87], Pojskić et al. [88], Pojskić et al. [89], Soslu et al. [117] |
|  | Shuttles (*n*) | Dawes & Spiteri [51], Pojskić et al. [88] |
| Incremental Treadmill Test | Estimated VO_2max_ (ml/kg/min) | Bolonchuk et al. [130], Caterisano et al. [53], Chatzinikolaou et al. [132], de Sousa Fortes et al. [59], Dragonea et al. [133], Fatouros et al. [125], Hunter & Hilyer [120], Hunter et al. [105], Lysenko [180], Maggioni et al. [58], Metaxas et al. [135], Milanović et al. [45], Narazaki et al. [57], Parr et al. [121], Ponce-González et al. [90], Sallet et al. [126], Stojanovic et al. [94], Tavino et al. [181] |
|  | Estimated VO_2peak_ (ml/kg/min) Heart rate at anaerobic threshold (bpm) Speed at anaerobic threshold (km/hr) VO_2_ at anaerobic threshold (ml/kg/min) Anaerobic threshold ^6^ (km/hr) Running economy (ml/kg/min) Velocity maximum (m/s) Vital capacity (L) Forced vital capacity (L) Forced expiratory volume (L) Respiratory exchange ratio VE_max_ (L/min) Blood lactate (mmol/L) VCO_2_ (ml/min) Heart rate (bpm) VE/VO_2_  VE/VCO_2_ Distance covered AZ (m) Time in AZ (s) VO_2max_/Heart rate | Boone & Bourgois [27], Boone et al. [131], McInnes et al.[134] Erkmen et al. [17], Metaxas et al. [135] Boone et al. [131]. Boone & Bourgois [27], Milanović et al. [45] Boone et al. [131] Boone & Bourgois [27], Maggioni et al. [58] Boone et al. [131] Boone et al. [131], Maggioni et al. [58], Milanović et al. [45] Metaxas et al. [135] Metaxas et al. [135] Metaxas et al. [135] Bolonchuk et al. [130], Metaxas et al. [135] Lysenko [180], Metaxas et al. [135] Boone & Bourgois [27], Maggioni et al. [58], Metaxas et al. [135] Bolonchuk et al. [130] Bolonchuk et al. [130], Maggioni et al. [58] Bolonchuk et al. [130] Bolonchuk et al. [130] Milanović et al. [45] Milanović et al. [45] Lysenko [180] |
| PACER | Laps (*n*) | Chen et al. [68] |
| 30-Minute Steady State Treadmill Test | MLSS ^7^ (mmol/L) Speed at MLSS (km/hr) Lactate turning point ^8^ | Dragonea et al. [133]  Dragonea et al. [133] Dragonea et al. [133] |
| 5-Minute Submaximal Run | Heart rate (bpm) Heart rate reserve (bpm) | Aoki et al. [63] Aoki et al. [63] |
| 1.5-Mile Run | Time (mins) | Hoffman et al. [103], Hoffman et al. [104] |
| Mognoni’s 6-Minute Run | Blood lactate concentration (mmol/L) Blood bicarbonate concentration (mmol/L) Heart rate (bpm) | Ferioli et al. [16], Ferioli et al. [136], Ferioli et al. [55] Ferioli et al. [16] Ferioli et al. [55] |
| Cooper’s 12-Minute run | Estimated VO_2max_ (ml/kg/min) Distance (m) | Omorczyk et al. [122], Gillam [119]  Omorczyk et al. [122] |
| 3-Minute Step Test | Peak heart rate (bpm) | Nikolaidis et al. [109] |
| 1-Mile Walk | Distance (m)  Estimated VO_2max_ (ml/kg/min) | Balabinis et al. [98]  Balabinis et al. [98] |
| Incremental Cycle Test | Estimated VO_2max_ (ml/kg/min) Estimated VO_2peak_ (ml/kg/min) Peak heart rate (bpm)  Respiratory exchange ratio Ventilatory threshold (ml/min/kg) Ventilation (L/min) Vital capacity (L) Relative peak power (W/kg) Power at anaerobic threshold (W) VO_2_ at anaerobic threshold (ml/kg/min) Heart rate at anaerobic threshold (bpm) Ventilation at anaerobic threshold (L/min) Ventilation peak (L/min) Peak power (W) VCO_2_ (L/min) VE/O_2_  V/CO_2_  Respiratory compensation point (ml/kg/min) Heart rate at respiratory compensation point (bpm) Heart rate at ventilatory threshold (bpm) W at ventilatory threshold (W/kg) W at respiratory compensation point (W/kg) Hypocapnic hyperventilation (ml/kg/min) Relative buffering capacity (ml/kg/min) End tidal partial pressure O_2_ (mmHg) End tidal partial pressure CO_2_ (mmHg) Isocapnic buffering (ml/kg/min) | Cormery et al. [15], Gocentas et al. [18], Laplaud et al. [80] Gocentas et al. [19] Gocentas et al. [19], Gocentas et al. [18] Cormery et al. [15], Gocentas et al. [19], Gocentas et al. [18], Laplaud et al. [80] Cormery et al. [15], Laplaud et al. [80] Cormery et al. [15], Laplaud et al. [80] Angyan et al. [43] Gocentas et al. [19], Gocentas et al. [18] Gocentas et al. [19], Gocentas et al. [18] Gocentas et al. [19], Gocentas et al. [18] Gocentas et al. [19], Gocentas et al. [18] Gocentas et al. [19], Gocentas et al. [18] Gocentas et al. [19], Gocentas et al. [18] Gocentas et al. [19], Gocentas et al. [18], Laplaud et al. [80] Cormery et al. [15] Cormery et al. [15] Cormery et al. [15] Cormery et al. [15] Cormery et al. [15] Cormery et al. [15] Cormery et al. [15], Laplaud et al. [80] Cormery et al. [15] Cormery et al. [15], Laplaud et al. [80] Cormery et al. [15], Laplaud et al. [80] Laplaud et al. [80] Laplaud et al. [80] Laplaud et al. [80] |
| *Note*: VO_2max_ = maximum oxygen uptake; ^5^ = reported as mean values of covered distances at 0-2m/s^2^, 2-3m/s^2^, and >3m/s^2^; arb. unit = arbitrary units;6 = anaerobic threshold determined at a lactate concentrate of 4 mmol/L; VEmax = maximal pulmonary ventilation; VCO_2_ = volume of carbon dioxide; VE = ventilation; AZ = anaerobic zone; PACER = Progressive Aerobic Cardiovascular Endurance Run; MLSS = Maximal lactate steady state; ^7^ = maximal blood lactate concentration that can be maintained for sustained period of time, without a significant rise in blood lactate; ^8^ = Lactate turning point was the second increase in blood lactate above the aerobic threshold and below VO_2max_; AT = Anaerobic threshold; VO_2_peak = highest recorded VO_2_ if criteria for VO_2_max is not met during maximal effort aerobic test; O_2_ = oxygen, CO_2_ = carbon dioxide; W at = corresponding power output. | | |

| **Supplementary Table 11.** Summary of reliability statistics reported for commonly used tests | | |
| --- | --- | --- |
| Test | Range | Reference |
| Countermovement Jump Vertical Jump Squat Jump | ICC: 0.778-0.995; CV: 1.9-8.1%  ICC: 0.566-0.994; CV: 1.6-16.2%  ICC: 0.89-0.98; CV: <5% | [16, 39, 48, 54, 55, 62, 63, 65, 67-71, 74, 75, 78, 82, 83, 85, 87, 88, 91, 93, 96]  [39, 59, 74, 82, 91, 96, 99, 100]  [63, 78, 89, 93, 96] |
| Linear Sprint Tests | ICC: 0.76-0.99; CV: 1.07-3.4% | [29, 30, 39, 59, 65, 67, 71, 86, 88, 89, 91, 93, 112, 114] |
| Agility T-Test  Modified Agility T-Test  Lane Agility Test  COD Y-COD  Y-CODST | ICC: 0.871-0.98; CV: 1.2-4.1%  NR  ICC: 0.99; CV: 8.71%  “previously reliable”  ICC: 0.80 NR | [49, 65, 67, 68, 70-72, 89, 112]  [51]  [113]  [85] |
| Reactive COD  Reactive Agility Test  Reactive Y-COD | “previously reliable”  ICC: 0.89-0.99; CV: 1.9-2%  ICC: 0.75-0.88; CV: 5-5.6% | [113, 118]  [4, 60]  [49, 85] |
| Back Squat  Bench Press | ICC: 0.93; CV: 2.4-2.7%  ICC: 0.93; CV: 2.4-2.5% | [65, 67, 71]  [65, 67, 71, 98, 100] |
| Full Court Shuttle Run  RAST  Wingate Anaerobic Test | ICC: 0.79; CV: 1.21%  ICC: 0.88  ICC: 0.94 | [125]  [88, 89, 99]  [98, 114, 127] |
| Incremental Treadmill Test  Yo-Yo IRL1  Multi-Stage Fitness Test | “previously reliable”  ICC: 0.93; CV: 4.9- ~5%  ICC: 0.95 | [57]  [16, 54, 63, 65, 67, 137]  [89] |
| *Notes:* ICC = intraclass correlation coefficient; CV = coefficient of variation; NR = not reported; COD = Change-of-Direction; Y-COD = Y-Shaped Change-of-Direction; Y-CODST = Y-Shaped Change-of-Direction speed test; RAST = Running-based Anaerobic Sprint Test; Yo-Yo IRL1 = Yo-Yo Intermittent Recovery Test Level 1. | | |
